# Supplementary material for: Cross-sectional evaluation of an asynchronous multiple mini-interview (MMI) in selection to health professions training programmes with 10 principles for fairness built-in
Source: BMJ Open. 2023 Oct 31;13(10):e074440. doi: 10.1136/bmjopen-2023-074440 (PMC10618971; doi:10.1136/bmjopen-2023-074440)

Appendix 2. Estimated hierarchical confirmatory factor analytic (CFA) model for the SAMMI responses, with a general factor (g) and seven specific factors (f1 to f7), the latter representing the seven questions in the interview. Standardised factor loading are shown.

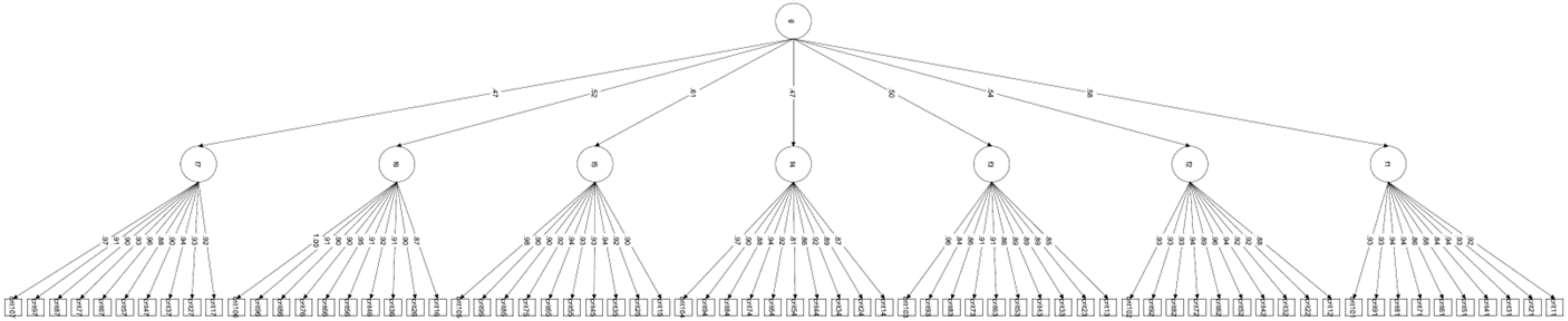

Supplement: Supplementary data [file bmjopen-2023-074440supp002.pdf]
