# Supplementary material for: Cross-sectional evaluation of an asynchronous multiple mini-interview (MMI) in selection to health professions training programmes with 10 principles for fairness built-in
Source: BMJ Open. 2023 Oct 31;13(10):e074440. doi: 10.1136/bmjopen-2023-074440 (PMC10618971; doi:10.1136/bmjopen-2023-074440)
Supplement: Supplementary data [file bmjopen-2023-074440supp003.pdf]

Appendix 3. Results of a parallel analysis using Pearson correlations on the response data to the SAMMI MMI.

Number of postulated factors	Mean of real data % of variance	Mean of random % of variance	95 percentile of random % of variance
1	29.2*	2.9	3.1
2	11.1*	2.8	3.0
3	9.5*	2.8	2.9
4	8.3*	2.7	2.8
5	7.6*	2.6	2.7
6	7.4*	2.6	2.7
7	6.2*	2.5	2.6
8	1.1	2.5	2.5
9	1.0	2.4	2.5

*Percentage of variance in the responses explained by the number of factors in the real data exceeded that for the random data.
