# Supplementary material for: Cross-sectional evaluation of an asynchronous multiple mini-interview (MMI) in selection to health professions training programmes with 10 principles for fairness built-in
Source: BMJ Open. 2023 Oct 31;13(10):e074440. doi: 10.1136/bmjopen-2023-074440 (PMC10618971; doi:10.1136/bmjopen-2023-074440)
Supplement: Supplementary data [file bmjopen-2023-074440supp005.pdf]

Supplementary Table 1: Applicant and interviewer top positives and negatives

Applicant				
Positives				
Theme	Sub theme detail	N=158 (%)	% of total comments	Illustrative quote
Ease	Access, intuitive, convenient, flexible, simple	63 (40)	21	*"Quick, not time consuming, simple"*<br>*"Flexible, you can do it at your own time. It reduces stage fright. It is convenient, you can do it in your own home…"*
Reduced stress	More relaxed in own home, take my time, breaks available, start when wish	45 (28)	15	*"Its more relaxing to be in your own home instead of a new environment which for me is much less intimidating resulting in perhaps me performing better in the interview".*<br>*"… there is no external party criticising you in the moment, something which personally takes away a fair amount of nerves and stress from the process…"*
Fairer	Reduced costs (travel), reduced time away from other responsibilities	39 (25)	13	*"Its much easier in regard to travel for those who live far away, don't have the funds or time or who have other commitments".*

	(caring), the practice portal and availability of question text helped neurodiverse applicants			*"Reduces the possibility of bias during the interview process".* *"More inclusive".* *"Another great thing about this was I could do a practice question to get used to the layout of the interview, so I wasn't going into the interview completely blind".*
'Meet' staff	Able to see more of the university's community	9 (7)	3	*"I liked that there were 7 different people asking the questions…"*
Covid safe	Travel not required	2 (1)	1	*"Considering the situation with the pandemic, the online interview has helped to continue the process of admissions in a positive way…"*
Negatives				
Theme	Sub theme detail	N=140 (%)	% of total comments	Illustrative quote
Limited direct communication	Less personal, no conversation	48 (34)	16	*"… Impersonal, I feel like I cant make a connection or read the interviewers body language in the interview which I feel doesn't allow me to act how I usually do, and it felt unnatural".*

MMI	Pressure felt due to the timed methodology, presence of the countdown timer, lack of question face validity.	45 (32)	15	*"4 minutes per question was quite pressuring to fill"* *"Ticking down time was off-putting".* *"Could have asked more personal questions such as why this university and tell us about yourself, there was too many scenario questions which quite put us on the spot".*
Anticipated technical issues	Wi-Fi cut out	27 (19)	9	*"Technical issue (my screen froze) potential upload failure, potential loss of internet connection".*
Don't get vibe of staff	Not directly meeting staff	2 (1)	1	*"Lack of interaction between the student-tutor, the dialogue"*
Cannot ask questions		18 (13)	6	*"Not personalised, not able to ask questions"*
Interviewer				
Positives				
Theme	Sub theme detail	N=132 (%)	% of total comments	Illustrative quote
Convenient	Can prioritise workload. Flexible Quicker	82 (62%)	37	*"Efficient, less time consuming and able to prioritise your workload accordingly and complete interviews in chunks as opposed to one long stint"*

	Less stressful			*Don't have the issue of trying to find someone to cover like when another meeting comes up at the same time…"*
Fairer	Less unconscious bias<br>Reduced travel [costs]	18 (14%)	7	*"Reduced bias from different people asking the questions".*<br>*"Equity... no student gets help with prompts more than others"*
East to navigate	Simple	12 (9%)	5	*"Easy to use with clear instructions"*
Benefits applicants	Less stressed in own environment	12 (9%)	6	*"Separating the two events [interview/applicant day] will help manage anxiety and stress"*
Applicant assessment	Can get a [better] sense of the applicant	4 (3%)	2	*"I feel I can get a sense of the applicant through this process".*
Less worried about technical issues	Not one time/date dependent	3 (2%)	2	*"Much less stressful as I don't have to worry about the internet connection".*
Ability to rewatch	If cause for concern'	1 (1%)	1	*"Ability to rewatch for clarification".*
Negatives				
Theme	Sub theme detail	N=90 (%)	% of total comments	Illustrative quote
Less personal	Don't get feel for applicant	27 (30%)	12	*"I think it's better for applicants to speak to someone in person"*

				"I question whether it produces the same quality and depth of response".
Critique of MMI methodology	Timed circuit/countdown timer	22 (24%)	10	*"Encourages to me to make a snap decision"*<br>*"Cant ask follow-on questions…permits no probing".*
Communication assessment and build rapport difficult	Less able to assess non-verbal communication, social skills, spontaneous cognitive ability.	21 (23%)	9	*"Little in the way of holism".*<br>*"Disconnect perhaps for applicants".*<br>*"Does not allow markers to assess a candidates ability to respond and adapt to others".*
Limited support for nervous/stressed applicant	Can not help if upset or stressed	8 (9%)	4	*"No opportunity to provide any support if the candidate appears upset.. if they are struggling mentally or emotionally"..*
Tech related process concerns	Tech issues<br>Stress of Zoom/Teams online mode<br>Repetitive watching videos<br>Disadvantages those not familiar with tech	4 (5%)	2	*"Robotic process"*<br>*"Repetitive and boring watching multiple videos"*<br>*"Some people find Teams/Zoom very stressful and therefore we dont see the best of the candidate".*<br>*"Technical issues for some student may give them a disadvantage".*
Did not have any	Stated 'none'	5 (6%)	2	*"Don't have any"*

				"None to mention".
University marketing	Does not show university as warm and welcoming	3 (3%)	1	*"We are very nice and they do not see that"* *"Current students often state they chose Surrey due to the friendliness of the staff when first meeting them, my concern is if this is the first impression".*
