# Supplementary material for: Cross-sectional evaluation of an asynchronous multiple mini-interview (MMI) in selection to health professions training programmes with 10 principles for fairness built-in
Source: BMJ Open. 2023 Oct 31;13(10):e074440. doi: 10.1136/bmjopen-2023-074440 (PMC10618971; doi:10.1136/bmjopen-2023-074440)
Supplement: Supplementary data [file bmjopen-2023-074440supp004.pdf]

Appendix 4: Cost Evaluation

These data represent an indicative example from this University based on 500 candidates over 7 days doing a seven station, four-minute MMI circuit.

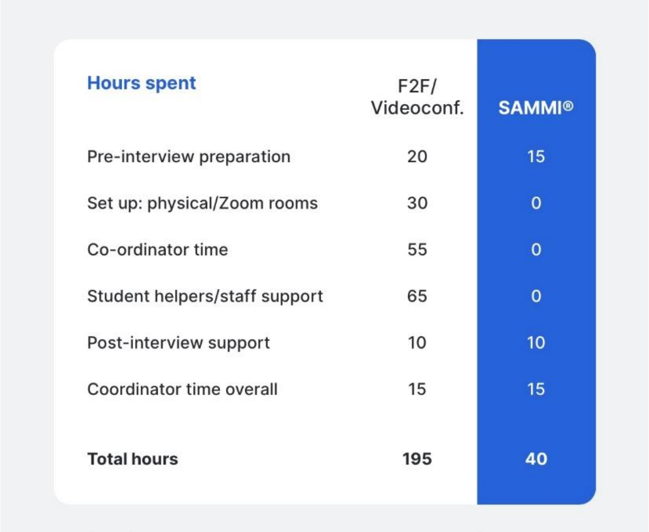

Hours spent	F2F/ Videoconf.	SAMMI®
Pre-interview preparation	20	15
Set up: physical/Zoom rooms	30	0
Co-ordinator time	55	0
Student helpers/staff support	65	0
Post-interview support	10	10
Coordinator time overall	15	15
**Total hours**	**195**	**40**
