# Supplementary material for: Cross-sectional evaluation of an asynchronous multiple mini-interview (MMI) in selection to health professions training programmes with 10 principles for fairness built-in
Source: BMJ Open. 2023 Oct 31;13(10):e074440. doi: 10.1136/bmjopen-2023-074440 (PMC10618971; doi:10.1136/bmjopen-2023-074440)
Supplement: Author's manuscript [file bmjopen-2023-074440.draft_revisions.pdf]

BMJ Open is committed to open peer review. As part of this commitment we make the peer review history of every article we publish publicly available.

When an article is published we post the peer reviewers' comments and the authors' responses online. We also post the versions of the paper that were used during peer review. These are the versions that the peer review comments apply to.

The versions of the paper that follow are the versions that were submitted during the peer review process. They are not the versions of record or the final published versions. They should not be cited or distributed as the published version of this manuscript.

BMJ Open is an open access journal and the full, final, typeset and author-corrected version of record of the manuscript is available on our site with no access controls, subscription charges or pay-per-view fees (http://bmjopen.bmj.com).

If you have any questions on BMJ Open's open peer review process please email info.bmjopen@bmj.com

BMJ Open

Cross-sectional evaluation of an asynchronous Multiple Mini Interview (MMI) in selection to health professions training programmes with ten principles for fairness built-in

Journal:	*BMJ Open*
Manuscript ID	bmjopen-2023-074440
Article Type:	Original research
Date Submitted by the Author:	06-Apr-2023

Complete List of Authors:	Callwood, Alison; University of Surrey, FHMS<br>Harris, Jenny; University of Surrey,<br>Gillam, Lee; University of Surrey,<br>Roberts, Sarah; University of Surrey Faculty of Health and Medical Sciences<br>Kubacki, Angela; St George's University of London, IMBE<br>Christidis, Angelos; University of Surrey Faculty of Engineering and Physical Sciences<br>Tiffin, Paul; University of York, Health Sciences

Keywords:	MEDICAL EDUCATION & TRAINING, Nursing Care, Health Education

SCHOLARONE™
Manuscripts

BMJ

I, the Submitting Author has the right to grant and does grant on behalf of all authors of the Work (as defined in the below author licence), an exclusive licence and/or a non-exclusive licence for contributions from authors who are: i) UK Crown employees; ii) where BMJ has agreed a CC-BY licence shall apply, and/or iii) in accordance with the terms applicable for US Federal Government officers or employees acting as part of their official duties; on a worldwide, perpetual, irrevocable, royalty-free basis to BMJ Publishing Group Ltd ("BMJ") its licensees and where the relevant Journal is co-owned by BMJ to the co-owners of the Journal, to publish the Work in this journal and any other BMJ products and to exploit all rights, as set out in our licence.

The Submitting Author accepts and understands that any supply made under these terms is made by BMJ to the Submitting Author unless you are acting as an employee on behalf of your employer or a postgraduate student of an affiliated institution which is paying any applicable article publishing charge ("APC") for Open Access articles. Where the Submitting Author wishes to make the Work available on an Open Access basis (and intends to pay the relevant APC), the terms of reuse of such Open Access shall be governed by a Creative Commons licence – details of these licences and which Creative Commons licence will apply to this Work are set out in our licence referred to above.

Other than as permitted in any relevant BMJ Author's Self Archiving Policies, I confirm this Work has not been accepted for publication elsewhere, is not being considered for publication elsewhere and does not duplicate material already published. I confirm all authors consent to publication of this Work and authorise the granting of this licence.

For peer review only

Cross-sectional evaluation of an asynchronous Multiple Mini Interview (MMI) in selection to health professions training programmes with ten principles for fairness built-in

Running head: evaluation of an asynchronous MMI

Corresponding author: Dr Alison Callwood Faculty of Health and Medical Sciences[1], University of Surrey, 30 Priestley Road, Guildford, Surrey, GU2 7YH, UK a.callwood@surrey.ac.uk. +44 7796956143

Dr Jenny Harris[1] jen.harris@surrey.ac.uk

Dr Lee Gillam[2] Faculty of Engineering and Physical Sciences, University of Surrey, Guildford, GU2 7XH, UK l.gillam@surrey.ac.uk

Mrs Sarah Roberts[1] sarah.roberts@surrey.ac.uk

Angela Kubacki[3] St George's University of London, London, UK akubacki@sgul.ac.uk

Angelos Christidis[2] Angelos.Christidis@surrey.ac.uk

P Tiffin[4] University of York, Your, UK paul.tiffin@york.ac.uk

Key Words

Multiple Mini Interview, equity, diversity and inclusion, fairness, asynchronous, online interviews

Word count 4826

For peer review only - http://bmjopen.bmj.com/site/about/guidelines.xhtml

Article summary

Study strengths and limitations of this study

- The theoretical and reliable approach aligned with an iterative process necessary to design a new technology to reduce bias.
- The large sample enabled us to assess psychometric properties with sub-group analysis for the first time in this context.
- The study provides perspectives from one large site; a necessary step to inform a planned international multi-site evaluation.
- The multi-method design provided insights necessary to embed fairness into online selection approaches in the absence of best practice guidance.

For peer review only

Introduction

Ensuring equity, inclusivity, and diversity in selection to health professions training programmes is recognised internationally as an ethical and practical imperative[1,2]. Globalisation and increased workforce pressures amplify this challenge[3]. Fulfilling our responsibility to ensure fair selection is complex due to unintended biases that are intrinsic to human assessment compounded by recent unprecedented change to online interviews in the absence of published evidence[4,5,6].

We understand that the adoption of new technologies in recruitment can be associated with substantial risk, as well as opportunity[7]. Historically, health professions' selection has been mainly face-to-face using unstructured or structured approaches including panel interviews, group interviews, assessment centres and multiple mini-interviews (MMIs)[8]. MMIs are a series of short, focused interactions with a number of different interviewers. The multi-question format featuring structured scoring proforma with interviewers who have no prior knowledge of applicants, is designed to mitigate the potential impact of interviewer bias[9]. MMIs have been shown to be a feasible, acceptable, valid, and reliable candidate selection approach across health professions. None-the-less, as a face-to-face method, MMIs can be costly, resource intensive and influenced by unintended bias[10].

The modality of interviews has changed dramatically in recent years. Pre-pandemic online interviews were a relatively uncommon occurrence in selection to health professions with limited evidence supporting or refuting their effectiveness. Approaches included Skype-based MMIs, asynchronous MMIs and asynchronous panel interviews [11]. Research outside the field of healthcare has shown asynchronous video interviews to be faster, cheaper, and require less employee time, easing scheduling burden and allowing for more applicants to be screened[12]. This can potentially increase the number of applicants who would have otherwise not had the opportunity to be interviewed.

For peer review only - http://bmjopen.bmj.com/site/about/guidelines.xhtml

During the pandemic, it was vital to ensure the continuance of recruitment to health professions. This resulted in rapid adaption to using online interviews facilitated by videoconference technology[13,14]. Recent research suggests online interviews like MMIs are feasible and acceptable provided reliable high-speed internet connection is available. However, access to reliable Wi-Fi is not always possible[11]. In an asynchronous approach, applicants record their interviews at a convenient time and place, alleviating the stress of potential technical issues.

In live synchronous interviews, nuanced inconsistencies in, for example, tone and intonation, can arise in the way interviewers ask questions. Consistency of questioning across applicants is assured in asynchronous interviews through the use of pre-recorded interview questions. Fairness is further ensured with the avoidance of nonadherence to set questions which can occur in live interviews when applicants and interviewers serendipitously find something in common and deviate to discuss this[12,15]. Moreover, evidence from recruitment to the police force demonstrates that fairness can also be optimised through incorporating language that supports the affirmation of values with increased probability of minority applicants passing an assessment by 50%.[16,17].

'Fairness' in this article is conceptualised as the quality of treating people equally or in a way that is right or reasonable. That encapsulates perceived fairness by participants as well as that borne out in the data. Consensus on the design of online interviews to optimise applicant accessibility and usability and mitigate potential unfairness issues for people across demographics, abilities, and disabilities is not readily available[15]. To address this, we successfully applied to Innovate UK, the United Kingdom's innovation agency (2020-2021) to build and evaluate what we believe was, the first (proof-of-concept, PoC) asynchronous videoconference facilitated interview and assessment system uniquely grounded in the MMI method (Figure 1).

For peer review only

Development summary

The asynchronous MMI is an on-demand videoconference interview where applicants log onto an online portal to complete their interview. The interview is a remote experience, where the system records applicants' short video responses to MMI questions in a timed process emulating the face-to-face MMI method. There is no synchronous, bidirectional communication, and no interviewer to interact with; instead, questions are pre-recorded by interviewers. Applicants' recorded responses are scored by an interview assessor at a convenient later date.

Once the PoC system was built, our vision was to ensure equitable access. To inform the design, we undertook a rapid review of literature published between 2011-2021, guided by a five-stage process[18] to help us understand how video-based interviews compare with face-to-face in terms of implementation and fairness including what makes for an optimised experience.

The databases searched included: Medline, PsycInfo, Embase, ProQuest (BNI) and Google Scholar/Google (grey literature). We included original articles published in English that reported studies involving candidates interviewed using video with/without face-to-face methods in health professions selection. Articles focusing on psychometric tests and situational judgement tests were excluded. For quality appraisal we used the Mixed Methods Appraisal Tool (MMAT)[19] combined with independent reviewer screening.

After review and removal of irrelevant and duplicates, 49 articles were included, 27 published pre-Covid (before Jan 2020) and 22 between Jan 2020 – Oct 2021 (Appendix 1). Of these, >90% originated in the US, the rest from Australia, Korea, and the UK. The majority were conducted in admissions to Medicine with two from Pharmacy and one Veterinary Medicine. Study methodologies included single

For peer review only - http://bmjopen.bmj.com/site/about/guidelines.xhtml

site cross-sectional/cohort or quasi-experimental studies. Meta-analysis/meta-synthesis were not possible due to study heterogeneity. Each article was read by up to two of the research team looking for insights and recommendations to optimise accessibility and fairness in online interviews. Indicative themes were elicited and assimilated into ten key principles. These included:

- Recognise potential issues with stereotype threats and belonging uncertainty that may impact on candidates' performance and use language that supports the affirmation of values e.g., "well done for getting this far".
- Incorporate encouraging words/phrases into the interview dialogue, as well as any communications circulated to applicants (e.g., "good luck").
- Soften the language of technical instructions e.g., "when you are ready …" or "when you have familiarised yourself with…".
- Reduce the verbal load of interview content particularly for neurodiverse applicants.
- Accommodate access and engagement for neurodiverse applicants with extra time, adjusted fonts and a tailored user-interface (UI) including background colours.
- Provide opportunities for candidates to familiarise themselves with the UI and format prior to their interview though a practice portal.
- Recommend generic, blank backgrounds for video or videoconference facilitated interviews or, if not possible, advise blurred backdrops.
- Ensure diversity of interviewers for pre-recorded videos and those assessing them including gender, age and ethnicity, experts-by-experience, and other stakeholders.
- Avoid culturally sensitive subject areas, language, age and ability bias in interview content.
- Ensure the use of inclusive, gender-neutral language with appropriate pronouns e.g., 'they/them/their'.

For peer review only - http://bmjopen.bmj.com/site/about/guidelines.xhtml

In Autumn 2021, the online asynchronous MMI system was re-configured to include these ten key principles. This summary of the development process of the asynchronous MMI system has been included for completeness and transparency.

The focus of the article is to detail the study undertaken to evaluate the psychometric properties of the asynchronous MMI. We aimed to explore reliability (internal consistency) for all users with sub-group analysis by key characteristics (age, gender, nationality, disability/additional support needs), construct validity, dimensionality, acceptability, and usability.

In this context disability refers to a person who self-identifies with physical or mental impairment, and the impairment has a substantial and long-term adverse effect on the person's ability to carry out normal day-to-day activities. Not all neurodivergent people consider themselves 'disabled' but instead neurological conditions are viewed a result of normal variations in the human genome but where additional support might be required. We therefore use the term 'support need' to include neurodiverse applicants.

Methods

The MMI questions were developed and tested by the university's community emulating our previously established in-person processes. A diverse range of individuals including academic staff, service users and practice partners were individually videorecorded asking MMI questions. The video recordings were uploaded onto the system, which was configured in a seven question, four-minute MMI with one minute between questions.

The asynchronous online MMI system was adopted for selection to Health Professions undergraduate programmes (Adult, Child, Mental Health Nursing, Midwifery, and Paramedic Science) at one UK university during 2021/22 recruitment cycle. All applicants to these programmes were invited to register for a one-week slot that was convenient for them. At the start of their selected slot, applicants

For peer review only - http://bmjopen.bmj.com/site/about/guidelines.xhtml

were emailed a link to the system. Here they could access the practice portal to check their Wi-Fi speed, familiarise themselves with the user interface/key functionality, become conversant with the process through a detailed instructional video, and practice a question. When they felt prepared, they could start their MMI. To emulate our previous in-person process, once they had begun, applicants could not stop their MMI and start again. Any technical issues including those without the necessary equipment and/or Wi-Fi connectivity were supported to enable their successful completion on an individual basis.

All individuals at the university who would usually interview applicants as part of their role took part in this new process. These included academic staff, service users and practice partners. They were invited to self-allocate to one-week interview assessment slots. Thereafter they received a link and code to access the system to review and assess applicant interviews against uploaded scoring rubrics live on the system. Each interviewer was allocated a number of applicant's video responses to a single question thereby aligning with the principles of an MMI. Also, emulating MMI methodology, a 'red flag' option could be ticked, and details populated in a text box if a cause for concern was raised in applicants' answers. Applicant's performance scores were downloaded at the end of the process to inform offer/reject decisions by university Admissions Officers.

University Admissions Officers conducted an internal resource evaluation to explore time and cost spent on the asynchronous MMI compared with face-to-face and Zoom-facilitated MMIs.

Design

This dual paradigmatic cross-sectional study was theoretically underpinned by Olsen and Eoyang's theory of 'Complex Adaptive Systems'[20]. This theory is characterised by an adaptive and iterative working style. This approach was appropriate when developing and optimising the system for fairness and reliability in the absence of any known precedent. We also grounded the system design in

For peer review only - http://bmjopen.bmj.com/site/about/guidelines.xhtml

Gilliland's[21] justice-based model. According to their theory, a selection system's adherence to procedural and distributive justice rules promotes applicants' perceptions of fairness. Procedural justice rules relate to the approach used to derive decisions, in this case an asynchronous MMIs. It includes the formal process (opportunity to perform and administration), explanation (feedback, information, and transparency), and interpersonal communication throughout the selection process. Distributive justice rules encompass adherence to equity when determining selection outcomes[21].

Participant recruitment

All applicants (n=712 at the data collection point) to the UK university and all interviewers (n=96) who assessed applicants were invited to evaluate the system.

Patient and Public Involvement

The university's service user group were supportive of the move to online asynchronous interviews. One service user acted as an interviewer by videorecording an MMI question. A further three were interview assessors, thereby continuing an established model at this university of service user involvement in recruitment. The service user group were also involved in the MMI question writing by reviewing draft questions and providing feedback. In the seven-question circuit, one practice partner video recorded an MMI question and eight assessed applicants' videos.

Data Collection

Applicant interview performance data, routinely collected to inform offer/reject decisions, were used in the reliability analyses. Applicant interviews were scored against 10 question-specific criteria on a seven-point Likert-type scale with descriptive anchors for the seven questions. Criteria details are withheld for test security. Applicant interview scores were summed for each applicant.

To assess usability, applicants were invited to complete an online evaluation questionnaire hosted on Qualtrics[22] once they had received their interview outcome decision.

For peer review only - http://bmjopen.bmj.com/site/about/guidelines.xhtml

To assess acceptability, interviewers were invited to evaluate the process at the end of the recruitment cycle through an online questionnaire also hosted on Qualtrics[22].

Analysis

We used applicant scores across questions to explore reliability (internal consistency) for all users and a randomly selected sub-sample self-reporting key characteristic including gender, age (<20 or 20+), nationality (UK/non-UK) and disability/support needs (absence/presence) using Cronbach's alpha (Stata, version 16.1, StataCorp LLC[23]) and scale dimensionality/construct validity using parallel analysis and confirmatory factor analysis (CFA) [24,25]. The parallel analysis and reliability values were derived using the software package FACTOR[26]. The ordinal factor analyses were conducted in Mplus[27] version 8.8. Usability and acceptability were explored with descriptive statistics (closed questions) and conventional content analysis[28] (open questions).

Results

Sample Characteristics

Data were available from 712 applicants to Nursing, Midwifery and Paramedic Science programmes at the data collection point on 1st May 2022 and for key characteristics for the sub-sample (n= 284) (Table 1). Disabilities self-reported by interviewers were reduced hearing and visual acuity. Applicants reported neurodiverse challenges across the spectrum of dyslexia, dyspraxia, and attention deficit hyperactivity disorder (ADHD).

Table 1: Participant self-identified characteristics

Interview Assessors n= 65					Applicant Sub-sample n=284		
		N	%			N	%
Age (years)	25 and under	0	0		<20	193	68

For peer review only - http://bmjopen.bmj.com/site/about/guidelines.xhtml

	26-35	8	13			
	36-45	18	28	>20	91	32
	45+	35	53			
	Prefer not to say	4	6			
Gender	Female	55	84	Female	230	81
	Male	7	10	Male	54	19
	Other	0	0	Other	0	0
	Prefer not to say	3	6	Prefer not to say	0	0
Disability/additional support needs	No	60	93	No	228	80
	Yes	5	7	Yes (64% neurodiverse, 34% mental health, 2% hearing challenges)	58	20
	Prefer not to say	0	0	Prefer not to say	0	0
Ethnicity	White (English/Welsh/Scottish/Northern Irish, Irish, Gypsy, Irish Traveller, Other White background)	61	94	UK/Ireland	229	81
	Mixed/multiple Ethnic Groups (White and Black Caribbean, White and Black African, Any other Mixed Background)	2	3	Non-UK/Ireland	55	19
	Asian/Asian British (Indian, Pakistani, Bangladeshi, Chinese, Any other Asian background)	0	0	N/A	N/A	
	Other (Arab, any other ethnic group)	0	0			
	Prefer not to say	2	3			
Role	University Health Sciences Staff	46	71	N/A	N/A	
	Practice Partner	10	16			

For peer review only

	Service user	9	13			
	Prefer not to say	0	0			

Reliability and assessment of fairness

Reliability

Applicant data were shown to be normally distributed (Kurtosis 0.000, p 0.005) and symmetrical with skewness 1.0.

Internal consistency was good-excellent across questions (for n=712 applicants) within each scenario (mean Cronbach's α 0.72 (range 0.64-0.89). Sub-group analyses showed similarly positive results with mean α Female/Male: 0.74/0.87 (range 0.70-0.89); age: <20years/ >21 years 0.76/0.83 (range 0.72-0.86), disability/additional challenges/non-disability: 0.78/0.88 (range 0.74-0.89) and UK/non-UK 0.78/0.0.77 (range 0.72-0.83) (Table 2).

Dimensionality/construct validity

The results of the parallel analysis suggested a maximum of seven dimensions underlay the response pattern. A Schmid-Leiman[29] transformation can be used to understand if a factor analytic model is best understood as a hierarchical in nature. This partitions observed variance into that explained by one or more general ('G') factors underlying three or more specific factors (a hierarchical model will be mathematically 'just identified' by three factors)[29]. The results of the Schmid-Leiman transformed factor analysis indicated a seven-factor solution, relating to the MMI scenarios, with an underlying general factor which substantially loaded on all the former seven factors. An ordinal Confirmatory Factor Analysis (CFA) to test this seven-factor hierarchical model showed an excellent fit to the data (Confirmatory Fit Index (CFI)=0.99, Tucker Lewis Index (TLI)=0.99, RMSE=0.034). In contrast, a one factor model showed a poor fit to the response data (CFI=0.70, TLI=0.69, RMSEA=0.19) (Appendix 2)

For peer review only

The results of the parallel analysis suggested a maximum number of seven plausible factors. The variance explained by additional postulated factors did not exceed that observed for the random data generated. (Appendix 3).

Table 2 MMI question reliability (internal consistency) N=712

Question (total mean)	Obs	Mean	St Dev	Min	Max	Item-test correlation	Item-rest correlation	Cronbach's alpha
1	765	48.6732	10.3966	10	70	0.6541	0.4677	0.68
2	795	51.57107	9.70229	10	70	0.6434	0.4532	0.67
3	790	51.08101	9.10597	10	70	0.599	0.421	0.69
4	809	50.23486	8.97655	10	70	0.5826	0.4001	0.70
5	762	50.27428	9.88047	10	70	0.6624	0.4941	0.67
6	765	51.79869	8.7486	20	70	0.6132	0.4484	0.69
7	712	50.10499	11.0865	14	70	0.6076	0.3871	0.70
Scale reliability coefficient								0.7216

Sub-group reliability n=284				
Question (total score)	Cronbach's alpha			
	Age	Self-identified gender	Self-declared disability	Country of birth

For peer review only

For peer review only - http://bmjopen.bmj.com/site/about/guidelines.xhtml

	< 20 years	> 20 years	Female	Male	Yes	Non	UK/Ireland	Non-UK/Ireland
1	0.74	0.83	0.73	0.87	0.88	0.76	0.78	0.74
2	0.74	0.83	0.72	0.87	0.88	0.76	0.77	0.75
3	0.75	0.83	0.74	0.87	0.87	0.77	0.78	0.77
4	0.77	0.85	0.76	0.89	0.88	0.79	0.79	0.79
5	0.73	0.82	0.71	0.86	0.87	0.75	0.76	0.73
6	0.77	0.84	0.76	0.87	0.87	0.79	0.79	0.79
7	0.79	0.87	0.78	0.88	0.89	0.80	0.80	0.86
Mean	0.78	0.85	0.77	0.88	0.89	0.80	0.8	0.80

Usability (applicants)

The online evaluation was completed by 210 applicants (29% response rate). The majority were under 20 years of age, self-identified as white female and with representation from across programmes (Table 3).

Table 3 Applicant Characteristics and usability evaluation

N=210			
Demographics		N	%
Age	<20	120	57
	20+	90	43

For peer review only - http://bmjopen.bmj.com/site/about/guidelines.xhtml

Self-identified gender	Female	172	82
	Male	36	17
	Other/non-binary	2	1
Nationality	UK/Ireland	134	64
	Non-UK/ Ireland	68	32
	Prefer not to say	8	4
Programme representation			
Nursing	Adult	42	20
	Child	34	16
	Mental Health	52	25
Midwifery		32	15
Paramedic Science		50	24
Closed question responses summary data			
Had not taken an asynchronous MMI like this before			94%
Found the instructions helpful/very helpful			90%
Did not experience technical issues			79%
Said the one minute between questions was ‘about right’			75%
Found the probe questions helpful/very helpful			66%
Four minutes was about the right amount of time			65%

Open question responses				
Question	Response %			
How do you feel about videorecording and uploading your MMI responses as part of a 'new look' interview process triggered by Covid-19 social distancing restrictions?	Happy/very happy	67	Not very happy	14

For peer review only

Please tell us about your views on online interviews generally	Accepting/very accepting	66	Not at all accepting	2
Do you see a future for online interviews like the one you have used with us?	Yes/definitely yes	68	Definitely not	9

The majority of applicants had not undertaken an asynchronous MMI previously. Overall applicants found the instructions either helpful or very helpful and did not experience any technical issues. From a setup perspective, four minutes was considered the right amount of time by over half with one-third stating it was too short. We asked additional overarching open questions regarding applicants' views on online interviews (Table 3) as well as their overall 'top' positives and 'top' negatives of system (Table 4 summary, Supplementary Table 1).

We received 158 separate positive comments and 140 negative comments in relation to the questions asking for 'top' positives and 'top' negatives, Notably, 93% of the positive comments centred around three themes: Ease (40%), reduced stress (28%) and fair (25%). We received fewer negative comments overall and these were split into two main themes: limited direct communication (34%) and critique of the MMI process itself (32%). Nineteen percent of respondents raised technical issues as a potential negative issue. However, it appears that as around 80% did not experience these in reality, this was more an anticipated concern rather than an experienced issue. The twelve respondents that did experience technical issues cited: buffering (3), frozen screen (2), crash (2), video skipping (2), screen scaling (1), microphone (1) and upload (1). They all subsequently successfully completed their interview at a second attempt.

For peer review only - http://bmjopen.bmj.com/site/about/guidelines.xhtml

Table 4 Summary Applicant and interviewer top positives and negatives

Applicant				
**Positives**				
Theme	Sub theme detail	N=158 (%)	% of total comments	Illustrative quote
Ease	Access, intuitive, convenient, flexible, simple	63 (40)	21	*"Quick, not time consuming, simple"*
Reduced stress	More relaxed in own home, take my time, breaks available, start when wish	45 (28)	15	*"It's more relaxing to be in your own home instead of a new environment which for me is much less intimidating resulting in perhaps me performing better in the interview".*
Fairer	Reduced costs (travel), reduced time away from other responsibilities (caring), the practice portal and availability of question text helped neurodiverse applicants	39 (25)	13	*"It's much easier in regard to travel for those who live far away, don't have the funds or time or who have other commitments".* *"Reduces the possibility of bias during the interview process".* *"…I could do a practice question to get used to the layout of the interview, so I wasn't going into the interview completely blind".*
'Meet' staff	Able to see more of the university's community	9 (7)	3	*"I liked that there were 7 different people asking the questions…"*
Covid safe	Travel not required	2 (1)	1	*"…Has helped to continue the process of admissions in a positive way…"*
**Negatives**				
Limited direct communication	Less personal, no conversation	48 (34)	16	*"… Impersonal, I feel like I can't make a connection or read the interviewers body language in the interview…"*
MMI	Pressure felt due to the timed methodology, presence of the countdown timer	45 (32)	15	*"4 minutes per question was quite pressuring to fill".* *"Ticking down time was off-putting".*
Technical issues	Wi-Fi cut out	27 (19)	9	"My *screen froze, potential upload failure, loss of connection".*
Don't get staff vibe	Not directly meeting staff	2 (1)	1	*"Lack of interaction between the student-tutor, the dialogue"*
Cannot ask questions		18 (13)	6	*"Not personalised, not able to ask questions"*

Interviewer				
Positives				
Convenient	Can prioritise workload. Flexible. Quicker<br>Less stressful	82 (62%)	37	*"Efficient, less time consuming and able to prioritise your workload and complete interviews in chunks as opposed to one long stint"*
Fairer	Less unconscious bias<br>Reduced travel [costs]	18 (14%)	7	*"Reduced bias from different people asking the questions".*<br>*"Equity... no student gets help with prompts more than others"*
East to navigate	Simple	12 (9%)	5	*"Easy to use with clear instructions"*
Benefits applicants	Less stressed in own environment	12 (9%)	6	*"Separating the two events [interview/applicant day] will help manage anxiety and stress"*
Applicant assessment	Can get a [better] sense of the applicant	4 (3%)	2	*"I feel I can get a sense of the applicant through this process".*
Less worried about technical issues	Not one time/date dependent	3 (2%)	2	*"Much less stressful as I don't have to worry about the internet connection".*
Ability to rewatch	If cause for concern'	1 (1%)	1	*"Ability to rewatch for clarification".*
Negatives				
Less personal	Don't get feel for applicant	27 (30%)	12	*"I think it's better for applicants to speak to someone in person".*<br>*"I question whether it produces the same quality of response".*
Critique of MMI methodology	Timed circuit/countdown timer	22 (24%)	10	*"Encourages to me to make a snap decision".*<br>*"Can't ask follow-on questions...permits no probing".*
Communication/building rapport difficult	Less able to assess non-verbal communication, social skills, spontaneous cognitive ability.	21 (23%)	9	*"Little in the way of holism".*<br>*"Disconnect perhaps for applicants".*
Limited support for nervous applicants	Cannot help if upset or stressed	8 (9%)	4	*"No opportunity to provide any support if the candidate appears upset.. if they are struggling mentally or emotionally"..*
Tech related process concerns	Tech issues<br>Stress of Zoom/Teams online mode	4 (5%)	2	*"Robotic process"*<br>*"Repetitive and boring watching multiple videos"*<br>*"Technical issues for some student may give them a disadvantage".*
Did not have any	Stated 'none'	5 (6%)	2	*"Don't have any"*

For peer review only - http://bmjopen.bmj.com/site/about/guidelines.xhtml

Acceptability (Interviewers)

Sixty-five interviewers took part in the online evaluation representing a 71% response rate. The majority were white British female university staff, over the age of 45 years with no declared disability (Table 1). This is representative of the University Faculty staff profile which is located in the Southeast of the UK.

Ninety-six percent (n=62) of interviewers found the system intuitive, easy to use and reported a perceived reduction in their stress. They primarily attributed this to increased convenience and flexibility. A 70%-time reduction was independently reported by our Admissions Officers. They estimated this based on the time spent on other interview approaches (face-to-face MMIs, Zoom-facilitated MMIs compared with our asynchronous MMIs; categorised into pre-interview communications, set up, staff recruitment (including covering sick time), interview facilitation and post-interview communications. The majority of the time saving was ascribed directly to the asynchronous modality which removed the need for staff to either facilitate face-to-face or online live interviews. Additionally, the asynchronous approach alleviated the pressures of last-minute non-availability of interviewers particularly practice partners as they were not tied to one scheduled day/time but had a time period (1 week) within which they could assess the interview recordings.

None of the interviewer assessors stated that they had used an asynchronous online MMI previously. Almost all (96%) found it easy to use and the user interface intuitive (92%). Less than 10% reported technical issues other than download issues which were resolved. Five percent were 'not accepting' of using the asynchronous MMI in the future.

We were interested to better understand whether interviewers felt communication could be assessed in an asynchronous modality. Thirty-three percent said 'yes', 54% 'somewhat' with 13% of respondents (n=6) responded 'no'. To generate more in depth insights we asked interviewers their top positives and negatives of the system. These are presented in Table 4. We received 132 positive and 90 negative comments. The majority of positive comments (94%) related to perceived

For peer review only - http://bmjopen.bmj.com/site/about/guidelines.xhtml

convenience (62%), fairness (14%) ease of navigation (9%) and benefits for the applicant (9%). Negative comments were split more evenly into perceptions of their being less personal (30%), critique of MMI methodology (24%), limited communication assessment and ability to build rapport (23%), and 5% had technology-related process concerns. Six percent cited 'none'.

Discussion

With ten principles for fairness designed-in these findings suggest the online interview is reliable, fair, time-efficient, and acceptable. The results of the factor analyses infer that there are scenario-level effects but that that these all relate to an underlying general factor indicative that the process is assessing different dimensions/constructs relevant to health care. These could be method effects or alternatively conceptualised as representing different aspects of the interpersonal procedural knowledge required to perform well on the MMI.

This platform is the only known custom built asynchronous online interview emulating the MMI methodology. Cognisant of Gilliland's[21] procedural and distributive justice rules, our aim was to optimise applicant accessibility through building principles for fairness into the MMI design and system set up. The reliability results and usability and acceptability evaluation signal this was largely achieved. More broadly however, organisations should carefully consider how even the act of inviting an applicant to complete an asynchronous interview may impact justice perceptions and attitudes. As with any other selection approach, applicants may question whether the method is a fair way to inform selection decisions[30]. These data suggest the configuration of our asynchronous MMI resulted in an equitable process particularly with the familiarisation enabled through the practice portal[15].
Rice[31] (p452) suggests that social presence or the "degree to which a medium is perceived as conveying the presence of the communicating participants" impacts on applicant acceptability. Social presence plays a central role in trust, enjoyment, and the perceived usefulness of the technological

For peer review only - http://bmjopen.bmj.com/site/about/guidelines.xhtml

For peer review only

medium[30]. There was by definition an absence of actual social presence in the asynchronous modality. However, we sought to mitigate this through softening the intersection between human and technology though design. The majority said they found the system intuitive/very intuitive and simple to use. This infers that the user interface design and inclusive language may have contributed towards a positive experience.

Applicants in this study (>66%) were either accepting or very accepting of the online asynchronous MMI with around one third (37%) agreeing we should 'definitely return' to face-to-face interviews. Notably, only 2% (n= 2) of applicants said they were 'not at all accepting' of the asynchronous MMI. The majority of applicants were 20 years or under. It could be suggested that a younger demographic are more familiar with and accepting of online technology and see it as part of their day-to-day lives. However, these data signal that there was no difference in reliability for those under 20 years compared to over 20 years old and that applicant performance was not impacted by their age.

Applicants' experiences, particularly perceptions of fairness, are of paramount concern for universities. The implications of fairness can extend to post-interview outcomes including offer/acceptance rates. It has been suggested that applicants who perceive that recruitment and selection processes are fair are more attracted to organisations[32,33,34]. Concurring with Brenner et al[12] applicants reported 'perceived fairness' as one of their top three positives of the asynchronous interview system. Their reasons include reduced travel costs and time away from caring responsibilities, as well as enhanced familiarisation of the process through the practice portal. MMI interviews were pre-recorded using inclusive language by diverse staff, representative of the University community. Additional time and an intuitive system user interface appeared to help meet the needs of neurodiverse applicants.

Incorporating the ten fairness principles were not difficult as many were low-cost design features that appeared to be impactful. We suggest these should become a default approach for online interviews

For peer review only - http://bmjopen.bmj.com/site/about/guidelines.xhtml

used in health professional selection to enable applicant performance optimisation. In view of the paucity of published evidence[15] these novel insights are informative as we inevitably move toward a technology-augmented future where asynchronous video interviews are considered a modality that is here to stay[15].

We received a 60:40 ratio of positive to negative comments from interview assessors. The largest contributor (62%) to the overall feedback related to positivity around convenience including ability to prioritise workload, flexibility, speed, and reduced stress. This was followed by ease of navigation and reduced bias. These findings corroborate evidence garnered outside the field of healthcare where asynchronous interviews have been found to be faster, cheaper, require less employee time and open the applicant funnel to allow more people to be interviewed than would otherwise have had the opportunity[15]. Nevertheless, communication skills are central to the role of a health professional and are assessed as a generic skill/attribute in each MMI question at this university. One sixth of interview assessors said they did not feel communication skills could be assessed while over a third stated they could in the asynchronous modality. Further research is warranted to better understand the intersection between humans and technology including barriers and enablers to effective communication and communication assessment.

The largest contributing negative comment (30%) focused on a perception that the process was less personal. We might have anticipated this to be higher. Steps being considered to enhance personal connection include live chats and Q&A sessions as well as increasing the number of 'offer holder-days' provided by the university where applicants are invited onto campus to engage with staff without the stress of an interview clouding the experience. Further research evaluating the effectiveness of this strategy is suggested.

Strengths and limitations of this study

For peer review only - http://bmjopen.bmj.com/site/about/guidelines.xhtml

Invitations to review the system were sent out after applicants received notification of their interview outcome. We understand that the interview outcome may have impacted on applicants' perception of the process. In this study, we were required to adhere to the university's preference. This will be reviewed in the design of any future study to minimise sampling and response bias.

The study was a theoretically driven mixed-methods approach with multi-programme perspectives offering important insights. However, we acknowledge the generalisability limitations of a single site design, but this was an essential step ahead of a planned large multi-site international evaluation.

While the sample size is large, the low response rate from applicants is a potential limitation and may infer selection bias. Users' views of such a technology can be impacted by many factors outside the scope of this research for example past experiences for which we were unable to account for.

It was not possible to conduct a comparison study 'pre/post system optimisation'. Covid necessitated a move to online interviews in unprecedented times. Data were not collected on applicant or interviewer views at the time given the burdens they were already facing. During that time however, we explored how fairness could be optimised through a review of published literature with findings embedded in our system (Appendix 1). In a high stakes admission process, it would be ethically wrong to conduct a prospective study now to compare with and without the 10 principles for fairness given the apparent benefits. Data is not available retrospectively due to the circumstances around Covid.

Collapsing applicant data into UK/Ireland and Non-UK Ireland was a necessary pragmatic decision based on lack of consistent reporting of ethnicity between the university (who did not routinely retain applicant ethnicity data until enrolment) and the UK University Central Admissions System (UCAS).

Rigour

In spite of reassurances in all communications, we were mindful that applicants might be concerned that their review of the interview process might impact on their interview outcome. We therefore sent out the invitation to evaluation once offer/reject decisions had been communicated to applicants.

For peer review only - http://bmjopen.bmj.com/site/about/guidelines.xhtml

All data in this evaluation were independently analysed and peer reviewed by multiple authors (AC, JH, SR, PT). In the qualitative content analysis, two authors (AC, SR) undertook the analysis independently. A <5% difference was noted between authors' findings. A compromise was mutually agreed in instances where this occurred.

Conclusion

With ten principles for fairness designed in, these findings suggest the asynchronous online interview is reliable, equitable, time-efficient, and acceptable. It is a moral imperative that healthcare workforces represent the societies which they serve, however unintended bias can influence selection decisions. In the absence of generically available consensus guidance on how fairness can be optimised in online interviews, these novel insights are applicable internationally across selection to health professions. Embedding fairness into the design of online interviews is relatively straightforward and low cost to implement. These data advance our understanding which is vital as we inevitably more towards a technology augmented future in the context of global workforce pressures.

.

.

For peer review only

For peer review only - http://bmjopen.bmj.com/site/about/guidelines.xhtml

References

1. Council of Deans of Health Equality, Diversity, and Inclusion: https://www.councilofdeans.org.uk/category/policy/equality-diversity-and-inclusion/. Accessed 06.03.23.
2. United Nations Department of Economic and Social Affairs 17 Sustainable Development Goals: https://sdgs.un.org/goals. Accessed 06.03.23.
3. https://www.oecd.org/publications/the-post-covid-19-rise-in-labour-shortages-e60c2d1c-en.htm
4. Sabesan V, Kapur N, Zemanek K, Levitt D, Vu T, Erp A. Implementation and evaluation of virtual multiple mini-interviews as a selection tool for entry into paediatric postgraduate training: A Queensland experience. Medical Teacher Published online: 30 Aug 2021. https://doi.org/10.1080/0142159X.2021.1967906
5. Cleland J, Chu J, Lim S, Low J, Lowe-Beer N, Kwek T. COVID-19: designing and conducting an on-line mini-multiple interview (MMI) in a dynamic landscape. Medical Teacher. 2020. May: 1-5. https://doi.org/10.1080/0142159X.2020.1762851.
6. Kok K, Chen L, Idris F, Mumin N, Ghani H, Zulkipli. Conducting multiple mini-interviews in the midst of COVID-19 pandemic. Korean J Med Educ. 2020 Dec; 32(4): 281–289. Published online 2020 Oct 28. doi: 10.3946/kjme.2020.175.
7. Fuller, R., Goddard, V., Nadarajah, V., Treasure-Jones, T. Yeates, P., Scott, K., Webb, A., Valter, K., Pyorala, E. 2022. Technology enhanced assessment: Ottawa consensus statement and Recommendations. Medical Teacher Pages 836-850 https://doi.org/10.1080/0142159X.2022.2083489
8. Patterson, F, Roberts, C, Hanson, M, Hampe, W, Eva, K, Ponnamperuma, G, Magzoub, M, Tekianhand, A, Cleland, J. Ottawa consensus statement: Selection and recruitment to the

For peer review only - http://bmjopen.bmj.com/site/about/guidelines.xhtml

healthcare professions. Medical Teacher. 2018;40(11):1-11. 10.1080/0142159X.2018.1498589

9. Eva K, Rosenfeld J, Reiter H, Norman G. An admissions OSCE: the multiple mini-interview. *Medical Education*. 2004; 38: 314-326.
10. Yusoff J. Multiple Mini Interview as an admission tool in higher education: Insights from a systematic review. J Taibah Univ Med Sci. 2019 Jun; 14(3): 203–240. doi: 10.1016/j.jtumed.2019.03.006
11. Selvam R, Hu R, Musselman R, Raiche I, Mclsaac, D, Moloo H (2021) Video-based interviewing in Medicine: A Scoping Review. Doi: https://doi.org/10.21203/rs.3.rs-468538/v1
12. Brenner, F. S., Ortner, T. M., & Fay, D. (2016). Asynchronous video interviewing as a new technology in personnel selection: The applicant's point of view. *Frontiers in Psychology, 7*, 863.
13. Huppert L A, Hsiao E C, Cho K C *et al.* Virtual interviews at graduate medical education training programs determining evidence-based best practices. *Acad Med* 2020. doi: 10.1097/ACM.0000000000003868.
14. Vining CC, Eng OS, Hogg ME, et al. Virtual Surgical Fellowship Recruitment During COVID-19 and Its Implications for Resident/Fellow Recruitment in the Future. Ann Surg Oncol. 2020;27(Suppl 3):911- 915. doi:10.1245/s10434-020-08623-2
15. Lukacik, E., Bourdage, J., Roulin, N. 2022. Into the void: A conceptual model and research agenda for the design and use of asynchronous video interviews. Human Resources Management Review 32 , 100789
16. Linos E, Reinhard J, Ruda S. Levelling the playing field in police recruitment: Evidence from a field experiment on test performance. *Public Administration*. 2017;95(4):943-956.
17. https://www.linkedin.com/business/talent/blog/future-of-recruiting

For peer review only

18. Bambra, C., Joyce, K., Bellis, M., Greatley, A., Greengross, S., Hughes, S., Lincoln, P. Lobstein, T., Naylor, C., Salay, R. Wiseman, M., Maryon-Davis, A. 2010 Reducing health inequalities in priority public health conditions: using rapid review to develop proposals for evidence-based policy. *Journal of Public Health*, Volume 32, Issue 4, December 2010, Pages 496–505, https://doi.org/10.1093/pubmed/fdq028

19. http://mixedmethodsappraisaltoolpublic.pbworks.com/w/file/fetch/127916259/MMAT_2018_criteria-manual_2018-08-01_ENG.pdf

20. Olsen E, Eoyang G. Facilitating organisational change: lessons from complexity science. San Francisco: Jossey-Bass; 2001.

21. Gilliland, S. 1983 The perceived fairness of selection systems: an organisational justice perspctive. Academy of Management Review Vol 18, No 4, pp 694-734

22. Qualtrics accesible at: https://www.qualtrics.com/uk/

23. Stata accessible at: https://www.stata.com/

24. Horn, J. L. (1965). A Rationale and Test for the Number of Factors in Factor Analysis. Psychometrika, 30, 179-185. https://doi.org/10.1007/BF02289447

25. Timmerman, M. E., & Lorenzo-Seva, U. (2011). Dimensionality assessment of ordered polytomous items with parallel analysis. *Psychological Methods, 16*(2), 209–220. https://doi.org/10.1037/a0023353

26. Lorenzo-Seva, U., Ferrando, P.J., 2006. FACTOR: a computer program to fit the exploratory factor analysis model. Behavior Research Methods 38, 88–91.

27. MPlus version 8. Los Angeles, CA: Muthén & Muthén, 2022

For peer review only - http://bmjopen.bmj.com/site/about/guidelines.xhtml

28. Hsieh H, Shannon S. Three Approaches to Qualitative Content Analysis. Qualitative Health Research. November.2005. http:// doi.org/10.1177/1049732305276687

29. Schmid, J., & Leiman, J. M. (1957). The development of hierarchical factor solutions. *Psychometrika*, *22*(1), 53–61.

30. Schinkel, S., van Vianen, A., & Van Dierendonck, D. (2013). Selection fairness and outcomes: A field study of interactive effects on applicant reactions. *International Journal of Selection and Assessment, 21*(1), 22–31.

31. Rice, R. E. (1993). Media appropriateness: Using social presence theory to compare traditional and new organizational media. *Human Communication Research, 19*(4), 451–484.

32. Oh, C. S., Bailenson, J. N., & Welch, G. F. (2018). A systematic review of social presence: Definition, antecedents, and implications. *Frontiers in Robotics and AI, 5*, 114.

33. Chapman, D. S., Uggerslev, K. L., Carroll, S. A., Piasentin, K. A., & Jones, D. A. (2005). Applicant attraction to organizations and job choice: A meta-analytic review of the correlates of recruiting outcomes. *Journal of Applied Psychology, 90*(5), 928–944. Chapman, D. S., Uggerslev, K. L., & Webster, J. (2003). Applicant reactions to face-to-face and technology

34. Truxillo, D. M., Bauer, T. N., Campion, M. A., & Paronto, M. E. (2002). Selection fairness information and applicant reactions: A longitudinal field study. *Journal of Applied Psychology, 87*(6), 1020–1031.

For peer review only

For peer review only

For peer review only - http://bmjopen.bmj.com/site/about/guidelines.xhtml

Figure 1: Asynchronous MMI infographic.

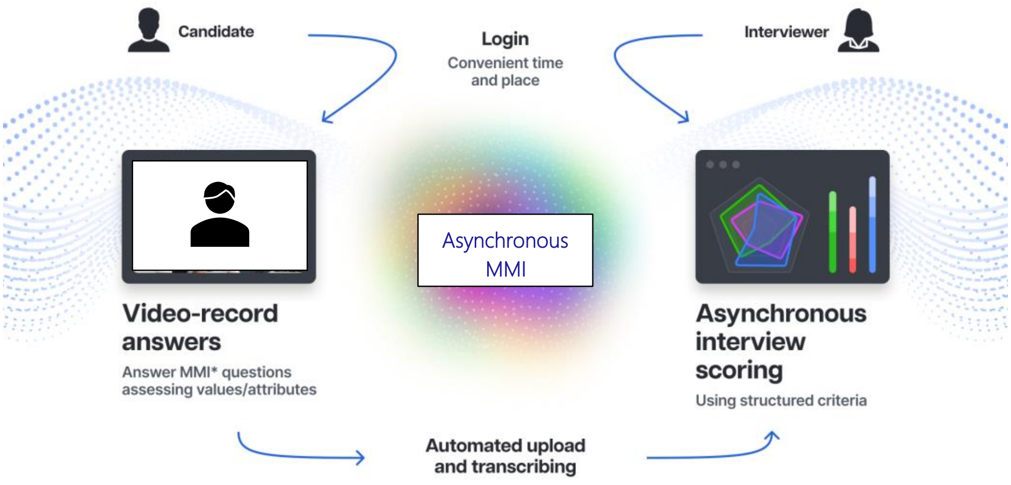

Appendix 1 Rapid Review Key References in Health Professions Selection

Further details available on reasonable request

35. Ballejos, M. P., Oglesbee, S., Hettema, J., Sapien, R. An equivalence study of interview platform: Does videoconference technology impact medical school acceptance rates of different groups?. Advances in Health Sciences Education: Theory and Practice. 2018;23(3):601–610. https://doi.org/http://dx.doi.org/10.1007/s10459-018-9817-2 Page 16/21

36. Bird, SB, Hern, G, Blomkalns, A, Deiorio NM, Haywood Y, Hiller KM, et al. Innovation in Residency Selection: The AAMC Standardized Video Interview. Academic Medicine: Journal of the Association of American Medical Colleges. 2019;94(10):1489–1497. https://doi.org/https://dx.doi.org/10.1097/ACM.0000000000002705

37. Bowers, K., Comp, G., Kalnow, A., Casey, J., Fraser, W., Lloyd, C., Little, A. Are Standardized Video Interview Scores Predictive of Interview Performance? Western Journal of Emergency Medicine. 2018;S18

38. Breitkopf, DM, Green, IC, Hopkins, MR, Torbenson, VE, Camp, CL, Turner, NS. Use of Asynchronous Video Interviews for Selecting Obstetrics and Gynaecology Residents. Obstetrics and Gynecology. 2019;134(4):9S-15S. https://doi.org/https://dx.doi.org/10.1097/AOG.0000000000003432

39. Chandler, N. M., Litz, C. N., Chang, H. L., Danielson, P. D., N.M., C., C.N., L., & H.L., C. Efficacy of Videoconference Interviews in the Paediatric Surgery Match. Journal of Surgical Education. 2019;76(2):420–426. https://doi.org/http://dx.doi.org/10.1016/j.jsurg.2018.08.010

40. Chung AS, Shah KH, Bond M, Ardolic B, Husain A, Li I, Cygan L, Caputo W, Shoenberger J, van Dermark J, Bronner J, Weizberg M. How Well Does the Standardized Video Interview Score Correlate with Traditional Interview Performance?. The Western Journal of Emergency Medicine. 2019;20(5):726–730. https://doi.org/https://dx.doi.org/10.5811/westjem.2019.7.42731

41. Cleland J, Chu J, Lim S, Low J, Lowe-Beer N, Kwek T. COVID-19: designing and conducting an on-line mini-multiple interview (MMI) in a dynamic landscape. Medical Teacher. 2020. May: 1-5. https://doi.org/10.1080/0142159X.2020.1762851

42. Daram, S. R., Wu, R., Tang, S.-J. J., S.R., D., R., W., Daram, S. R., … Tang, S.-J. J. Interview from anywhere: Feasibility and utility of web-based videoconference interviews in the gastroenterology fellowship selection process. American Journal of Gastroenterology. 2014;109(2):155–159. https://doi.org/10.1038/ajg.2013.278

43. Davis M, Haas M, Gottlieb M *et al.* Zooming In Versus Flying Out: Virtual Residency Interviews in the Era of COVID-19. *AEM Educ Train* 2020; 4: 443-446.

44. Egan, D. J., Husain, A., Bond, M. C., Caputo, W., Cygan, L., Van Dermark, J., et al. Standardized video interviews do not correlate to United States medical licensing examination step 1 and step 2 scores. Page 18/21 The Western Journal of Emergency Medicine. 2019;20(1):87–91. https://doi.org/http://dx.doi.org/10.5811/westjem.2018.11.39730

45. Edje, L., Miller, C., Kiefer, J., & Oram, D. Using Skype as an Alternative for Residency Selection Interviews. Journal of Graduate Medical Education. 2013;5(3):503–505. https://doi.org/10.4300/jgme-d-12-00152.1

46. Gallahue, F. E., Hiller, K. M., Bird, S. B., Calderone Haas, M. R., Deiorio, N. M., Hern, H. G., et al. The AAMC Standardized Video Interview: Reactions and Use by Residency Programs During the 2018 Application Cycle. Academic Medicine: Journal of the Association of American Medical Colleges. 2019;94(10):1506–1512. https://doi.org/https://dx.doi.org/10.1097/ACM.0000000000002714

47. Grova MM, Donohue SJ, Meyers MO, Kim HJ, Ollila DW. Direct Comparison of In-Person Versus Virtual Interviews for Complex General Surgical Oncology Fellowship in the COVID-19 Era. Ann Surg Oncol. 2021;28(4):1908-1915. doi:10.1245/s10434-020-09398-2

48. Hakes, E., Schnapp, B., Ritter, D., Kraut, A., Fallon, S., Brown, K., Westergaard, M. Communication and Professionalism: Comparing Standardized Video Interview Scores to Faculty Gestalt. Society for Academic Emergency Medicine. 2018;S276.

49. Hall, MM, Lewis, JJ, Joseph, JW, Ketterer, AR, Rosen, CL, Dubosh, NM. Standardized Video Interview Scores Correlate Poorly with Faculty and Patient Ratings. The Western Journal of Emergency Medicine. 2019;21(1):145–148. https://doi.org/https://dx.doi.org/10.5811/westjem.2019.11.44054

50. Healy, W. L., Bedair, H., & W.L., H. Videoconference Interviews for an Adult Reconstruction Fellowship: Lessons Learned. The Journal of Bone and Joint Surgery. American Volume. 2017;99(21):e114. https://doi.org/https://dx.doi.org/10.2106/JBJS.17.00322

51. Hopson, L. R., Dorfsman, M. L., Branzetti, J., Gisondi, M. A., Hart, D., Jordan, J., et al. Comparison of the Standardized Video Interview and Interview Assessments of Professionalism and Interpersonal Communication Skills in Emergency Medicine. AEM Education and Training. 2019;3(3):259–268. https://doi.org/https://dx.doi.org/10.1002/aet2.10346

52. Humbert, A., Pettit, K., Mugele, J., Turner, J., Morgan, Z., & Palmer, M. Correlation of the Standard Video Interview Score With an Established Application Review Process. Society for Academic Emergency Medicine. 2018;S98.

53. Huppert L A, Hsiao E C, Cho K C *et al.* Virtual interviews at graduate medical education training programs determining evidence-based best practices. *Acad Med* 2020. doi: 10.1097/ACM.0000000000003868.

---

54. Husain, A., Li, I., Ardolic, B., Bond, M. C., Shoenberger, J., Shah, K. H., et al. The Standardized Video Interview: How Does It Affect the Likelihood to Invite for a Residency Interview?. AEM Education and Training. 2019;3(3):226–232. https://doi.org/https://dx.doi.org/10.1002/aet2.10331

Inzana, K, Vanderstichel, R, Newman, S. Virtual Multiple Mini-Interviews for Veterinary Admissions. https://doi.org/10.3138/jvme-2020-0107 J Of Veterinary Medical Education

For peer review only

56. Joshi, A., Bloom, D. A., Spencer, A., Gaetke-Udager, K., & Cohan, R. H. Video Interviewing: A Review and Recommendations for Implementation in the Era of COVID-19 and Beyond. Academic radiology. 2020;27(9):1316–1322. https://doi.org/10.1016/j.acra.2020.05.020

57. Kok K, Chen L, Idris F, Mumin N, Ghani H, Zulkipli. Conducting multiple mini-interviews in the midst of COVID-19 pandemic. Korean J Med Educ. 2020 Dec; 32(4): 281–289. Published online 2020 Oct 28. doi: 10.3946/kjme.2020.175.

58. Krauss, W., Egan, D., Bond, M., Husain, A., White, M., Taylor, T., et al. Correlation Between Emergency Medicine Residency Applicant's Standardized Video Interview Scores and United States Medical Licensing Examination Results. Society for Academic Emergency Medicine. 2018;S83

Kyong-Jee Kim, Nam Young Lee & Bum Sun Kwon. Benefits and Feasibility of Using Videos to Assess Medical School Applicants' Empathetic Abilities in Multiple Mini Interviews. *Medical Science Educator* volume 31, pages175–181 (2021)C

60. Lewis, J., Hall, M., Joseph, J., Dubosh, N., Ullman, E. Standardized Video Interview Scores Do Not Correlate With Attending Evaluations. Society for Academic Emergency Medicine, 2018;S229.

61. M., S.R., E., L.M., B., M.M., A., F.M., D., J.C., E., … H., F. Initial Experience with a Virtual Platform for Advanced Gastrointestinal Minimally Invasive Surgery Fellowship Interviews. Journal of the American College of Surgeons. 2020;231(6):670–678. https://doi.org/http://dx.doi.org/10.1016/j.jamcollsurg.2020.08.768

62. McAteer, R., Sundaram, S., Harkisoon, S., & Miller, J. Videoconference Interviews: A Timely Primary Care Residency Selection Approach. Journal of Graduate Medical Education. 2020;12(6):737–744. https://doi.org/https://dx.doi.org/10.4300/JGME-D-20-00248.1

63. McHugh, M., Kulstad, C., Van Dermark, J., Bischof, J. Do Standardized or Traditional Interview Questions Correlate With the Standardized Video Interview? Society for Academic Emergency Medicine. 2019;S216

64. Molina, G., Mehtsun, W. T., Qadan, M., Hause, K. C., Raut, C. P., & Fairweather, M. Virtual Interviews for the Complex General Surgical Oncology Fellowship: The Dana-Farber/Partners Experience. Annals of Surgical Oncology. 2020;27(9):3103–3106. https://doi.org/https://dx.doi.org/10.1245/s10434-020- 08778-y

65. Nutter, A., La Rosa, M., Olson, G. Perception of Candidates and Faculty on Maternal Fetal Medicine Fellowship Videoconference Interviewing. Obstetrics and Gynecology. 2020;S75.

Nwora C, M.D.[a] Allred, D, M.D.[b] Verduzco-Gutierrez, M, M.D.[c] Mitigating Bias in Virtual Interviews for Applicants Who are Underrepresented in Medicine. Journal of the National Medical Association Volume 113, Issue 1, February 2021, Pages 74-76

67. Patel T, Bedi H, Deitte L, Lewis P *et al.* Brave New World: Challenges and Opportunities in the COVID-19 virtual interview season. *Acad Radiol* 2020; 27: 1456-1460.

68. Proost, K., Germeys, F., & Vanderstukken, A. Applicants' pre-test reactions towards video interviews: the role of expected chances to demonstrate potential and to use nonverbal cues.

For peer review only

European Journal of Work and Organizational Psychology. 2021;30(2):265-273. DOI: 10.1080/1359432X.2020.1817975

69. Sabesan V, Kapur N, Zemanek K, Levitt D, Vu T, Erp A. Implementation and evaluation of virtual multiple mini-interviews as a selection tool for entry into paediatric postgraduate training: A Queensland experience. Medical Teacher Published online: 30 Aug 2021.

https://doi.org/10.1080/0142159X.2021.1967906

70. Schnapp, B. H., Ritter, D., Kraut, A. S., Fallon, S., Westergaard, M. C. Assessing residency applicants' communication and professionalism: Standardized video interview scores compared to faculty gestalt. Western Journal of Emergency Medicine. 2019;20(1):132–137. https://doi.org/http://dx.di.org/10.5811/westjem.2018.10.39709

71. Shah SK, Arora, S, Skipper B, Kalishman S, Timm TC, Smith AY. Randomized evaluation of a web based interview process for urology resident selection. Journal of Urology. 2012;187(4):1380–1384. https://doi.org/http://dx.doi.org/10.1016/j.juro.2011.11.108

72. Singh, N D, DeMesa, DO, MPH, Pritzlaff, S, MD, Jung, M MD, MBA, Green, C MA, LMFT. Implementation of Virtual Multiple Mini-Interviews for Fellowship Recruitment . *Pain Medicine*, Volume 22, Issue 8, August 2021, Pages 1717–1721, https://doi.org/10.1093/pm/pnab141

73. Sripad, A. Videoconference Interviews for Female Pelvic Medicine and Reconstructive Surgery Fellowship During a Pandemic: The Candidate Experience. Female Pelvic Medicine & Reconstructive Surgery. 2020;S181.

74. Staicu, M., Hamby, C., Wychowski, M., Reiss, B. Facetime faceoff: evaluation of video conferencing as a novel pre-interview screen for a PGY-1 pharmacy residency. 2015;E182.

75. Temple, M., Lagzdins, M. Streamlining the residency interview process using Web-based teleconferencing. American Journal of Health-System Pharmacy. 2014;71:697-701. Doi: 10.2145/ajhp130406

76. Tiller D, O'Mara D, Rothnie I, Dunn S, Lee L, Roberts C. Internet-based multiple mini-interviews for candidate selection for graduate entry programmes. Med Educ. 2013;47(8):801-810. doi:10.1111/medu.12224

77. Turpin C, Steele K, Matuk-Villazon O, Rowland, K, Dayton, Horn, K. Rapid Transition to a Virtual Multiple Mini-Interview Admissions Process: A New Medical School's Experience During the COVID-19 Pandemic. Academic Medicine: August 2021 - Volume 96 - Issue 8 - p 1152-1155. doi: 10.1097/ACM.0000000000004179.

78. Ungtrakul T, Lamlertthon W, Boonchoo B, ,Auewarakul C. Virtual Multiple Mini-Interview during the COVID-19 Pandemic. Medical Education Adaptation. **https://doi.org/10.1111/medu.14207**

79. Vadi, M. G., Malkin, M. R., Lenart, J., Stier, G. R., Gatling, J. W., & Applegate, R. L. Comparison of web-based and face-to-face interviews for application to an anaesthesiology training program: a

For peer review only

pilot study. International Journal of Medical Education. 2016;7:102–108. https://doi.org/10.5116/ijme.56e5.491a

80. Vining CC, Eng OS, Hogg ME, et al. Virtual Surgical Fellowship Recruitment During COVID-19 and Its Implications for Resident/Fellow Recruitment in the Future. Ann Surg Oncol. 2020;27(Suppl 3):911- 915. doi:10.1245/s10434-020-08623-2

81. Willis, J., Surles, T., Silverberg, M., Kendall, S., LoCascio, H., Gernsheimer, J., Schechter, J., Regan, A., Smith, T. Are Standardized Video Interview Scores Predictive of Interview Performance? Western Journal of Emergency Medicine. 2018;S5-S6.

82. Winfield-Dial, A., Chhabra, N., Schindlbeck, M., & Bowman, S. Demographic Differences Between High and Low Scorers on the Standardized Video Interview. Western Journal of Emergency Medicine. 2018;S48. https://escholarship.org/uc/item/68c0x4pf

83. Winfield-Dial, A., Chhabra, N., Schindlbeck, M., & Bowman, S. Applicant Attitudes Towards the Standardized Video Interview - An Interim Analysis. Western Journal of Emergency Medicine. 2018;S4. https://escholarship.org/uc/item/5xp7f587 Page 17/21

84. Yolanda,S, Wisnu, W, Wahjudi, J and Findyartini A. Adaptation of internet-based multiple mini-interviews in a limited-resource medical school during the coronavirus disease 2019 pandemic. Korean J Med Educ. 2020 Dec; 32(4): 281–289. doi: 10.3946/kjme.2020.175

For peer review only

Appendix 2. Estimated hierarchical confirmatory factor analytic (CFA) model for the SAMMI responses, with a general factor (g) and seven specific factors (f1 to f7), the latter representing the seven questions in the interview. Standardised factor loading are shown.

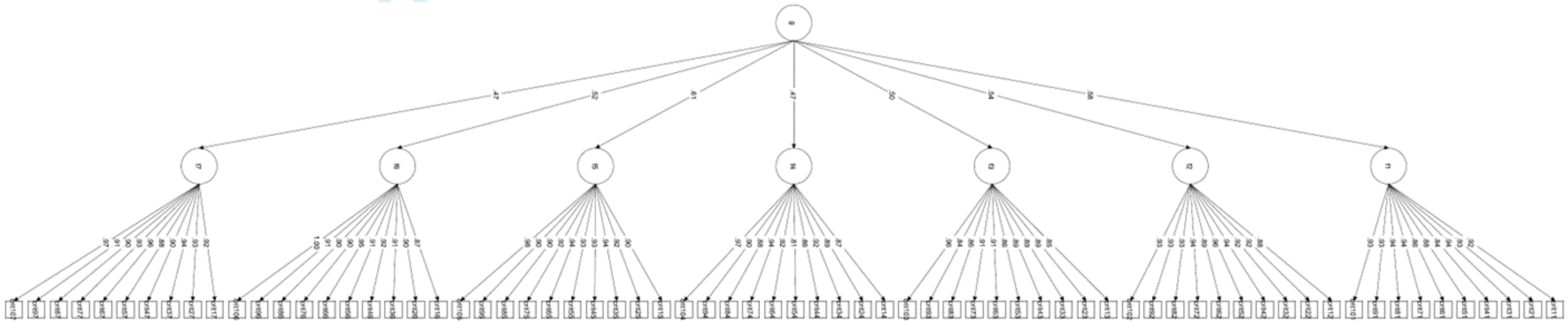

For peer review only - http://bmjopen.bmj.com/site/about/guidelines.xhtml

Appendix 3. Results of a parallel analysis using Pearson correlations on the response data to the SAMMI MMI.

Number of postulated factors	Mean of real data % of variance	Mean of random % of variance	95 percentile of random % of variance
1	29.2*	2.9	3.1
2	11.1*	2.8	3.0
3	9.5*	2.8	2.9
4	8.3*	2.7	2.8
5	7.6*	2.6	2.7
6	7.4*	2.6	2.7
7	6.2*	2.5	2.6
8	1.1	2.5	2.5
9	1.0	2.4	2.5

*Percentage of variance in the responses explained by the number of factors in the real data exceeded that for the random data.

For peer review only

Supplementary Table 1: Applicant and interviewer top positives and negatives

Applicant				
Positives				
Theme	Sub theme detail	N=158 (%)	% of total comments	Illustrative quote
Ease	Access, intuitive, convenient, flexible, simple	63 (40)	21	*"Quick, not time consuming, simple"* *"Flexible, you can do it at your own time. It reduces stage fright. It is convenient, you can do it in your own home…"*
Reduced stress	More relaxed in own home, take my time, breaks available, start when wish	45 (28)	15	*"Its more relaxing to be in your own home instead of a new environment which for me is much less intimidating resulting in perhaps me performing better in the interview".* *"… there is no external party criticising you in the moment, something which personally takes away a fair amount of nerves and stress from the process…"*
Fairer	Reduced costs (travel), reduced time away from other responsibilities	39 (25)	13	*"Its much easier in regard to travel for those who live far away, don't have the funds or time or who have other commitments".*

For peer review only

	(caring), the practice portal and availability of question text helped neurodiverse applicants			*"Reduces the possibility of bias during the interview process".* *"More inclusive".* *"Another great thing about this was I could do a practice question to get used to the layout of the interview, so I wasn't going into the interview completely blind".*
'Meet' staff	Able to see more of the university's community	9 (7)	3	*"I liked that there were 7 different people asking the questions…"*
Covid safe	Travel not required	2 (1)	1	*"Considering the situation with the pandemic, the online interview has helped to continue the process of admissions in a positive way…"*
Negatives				
Theme	Sub theme detail	N=140 (%)	% of total comments	Illustrative quote
Limited direct communication	Less personal, no conversation	48 (34)	16	*"… Impersonal, I feel like I cant make a connection or read the interviewers body language in the interview which I feel doesn't allow me to act how I usually do, and it felt unnatural".*

For peer review only

MMI	Pressure felt due to the timed methodology, presence of the countdown timer, lack of question face validity.	45 (32)	15	*"4 minutes per question was quite pressuring to fill"* *"Ticking down time was off-putting".* *"Could have asked more personal questions such as why this university and tell us about yourself, there was too many scenario questions which quite put us on the spot".*
Anticipated technical issues	Wi-Fi cut out	27 (19)	9	*"Technical issue (my screen froze) potential upload failure, potential loss of internet connection".*
Don't get vibe of staff	Not directly meeting staff	2 (1)	1	*"Lack of interaction between the student-tutor, the dialogue"*
Cannot ask questions		18 (13)	6	*"Not personalised, not able to ask questions"*
Interviewer				
Positives				
Theme	Sub theme detail	N=132 (%)	% of total comments	Illustrative quote
Convenient	Can prioritise workload. Flexible Quicker	82 (62%)	37	*"Efficient, less time consuming and able to prioritise your workload accordingly and complete interviews in chunks as opposed to one long stint"*

For peer review only

	Less stressful			*Don't have the issue of trying to find someone to cover like when another meeting comes up at the same time…"*
Fairer	Less unconscious bias<br>Reduced travel [costs]	18 (14%)	7	*"Reduced bias from different people asking the questions".*<br>*"Equity... no student gets help with prompts more than others"*
East to navigate	Simple	12 (9%)	5	*"Easy to use with clear instructions"*
Benefits applicants	Less stressed in own environment	12 (9%)	6	*"Separating the two events [interview/applicant day] will help manage anxiety and stress"*
Applicant assessment	Can get a [better] sense of the applicant	4 (3%)	2	*"I feel I can get a sense of the applicant through this process".*
Less worried about technical issues	Not one time/date dependent	3 (2%)	2	*"Much less stressful as I don't have to worry about the internet connection".*
Ability to rewatch	If cause for concern'	1 (1%)	1	*"Ability to rewatch for clarification".*
Negatives				
Theme	Sub theme detail	N=90 (%)	% of total comments	Illustrative quote
Less personal	Don't get feel for applicant	27 (30%)	12	*"I think it's better for applicants to speak to someone in person"*

For peer review only

				"I question whether it produces the same quality and depth of response".
Critique of MMI methodology	Timed circuit/countdown timer	22 (24%)	10	*"Encourages to me to make a snap decision"*<br>*"Cant ask follow-on questions…permits no probing".*
Communication assessment and build rapport difficult	Less able to assess non-verbal communication, social skills, spontaneous cognitive ability.	21 (23%)	9	*"Little in the way of holism".*<br>*"Disconnect perhaps for applicants".*<br>*"Does not allow markers to assess a candidates ability to respond and adapt to others".*
Limited support for nervous/stressed applicant	Can not help if upset or stressed	8 (9%)	4	*"No opportunity to provide any support if the candidate appears upset.. if they are struggling mentally or emotionally"..*
Tech related process concerns	Tech issues<br>Stress of Zoom/Teams online mode<br>Repetitive watching videos<br>Disadvantages those not familiar with tech	4 (5%)	2	*"Robotic process"*<br>*"Repetitive and boring watching multiple videos"*<br>*"Some people find Teams/Zoom very stressful and therefore we dont see the best of the candidate".*<br>*"Technical issues for some student may give them a disadvantage".*
Did not have any	Stated 'none'	5 (6%)	2	*"Don't have any"*

For peer review only

				"None to mention".
University marketing	Does not show university as warm and welcoming	3 (3%)	1	*"We are very nice and they do not see that"* *"Current students often state they chose Surrey due to the friendliness of the staff when first meeting them, my concern is if this is the first impression".*

For peer review only

STROBE Statement—checklist of items that should be included in reports of observational studies

	Item No	Recommendation	Page No
**Title and abstract**	1	(*a*) Indicate the study's design with a commonly used term in the title or the abstract	1
		(*b*) Provide in the abstract an informative and balanced summary of what was done and what was found	3-4
**Introduction**			
Background/rationale	2	Explain the scientific background and rationale for the investigation being reported	5-9
Objectives	3	State specific objectives, including any prespecified hypotheses	9
**Methods**			
Study design	4	Present key elements of study design early in the paper	9-10
Setting	5	Describe the setting, locations, and relevant dates, including periods of recruitment, exposure, follow-up, and data collection	10
Participants	6	(*a*) *Cohort study*—Give the eligibility criteria, and the sources and methods of selection of participants. Describe methods of follow-up<br>*Case-control study*—Give the eligibility criteria, and the sources and methods of case ascertainment and control selection. Give the rationale for the choice of cases and controls<br>*Cross-sectional study*—Give the eligibility criteria, and the sources and methods of selection of participants	11
		(*b*) *Cohort study*—For matched studies, give matching criteria and number of exposed and unexposed<br>*Case-control study*—For matched studies, give matching criteria and the number of controls per case	
Variables	7	Clearly define all outcomes, exposures, predictors, potential confounders, and effect modifiers. Give diagnostic criteria, if applicable	12
Data sources/ measurement	8*	For each variable of interest, give sources of data and details of methods of assessment (measurement). Describe comparability of assessment methods if there is more than one group	*12*
Bias	9	Describe any efforts to address potential sources of bias	30
Study size	10	Explain how the study size was arrived at	11
Quantitative variables	11	Explain how quantitative variables were handled in the analyses. If applicable, describe which groupings were chosen and why	12
Statistical methods	12	(*a*) Describe all statistical methods, including those used to control for confounding	12
		(*b*) Describe any methods used to examine subgroups and interactions	12
		(*c*) Explain how missing data were addressed	12
		(*d*) *Cohort study*—If applicable, explain how loss to follow-up was addressed<br>*Case-control study*—If applicable, explain how matching of cases and controls was addressed<br>*Cross-sectional study*—If applicable, describe analytical methods taking account of sampling strategy	12
		(*e*) Describe any sensitivity analyses	

For peer review only - http://bmjopen.bmj.com/site/about/guidelines.xhtml

**Results**			
Participants	13*	(a) Report numbers of individuals at each stage of study—eg numbers potentially eligible, examined for eligibility, confirmed eligible, included in the study, completing follow-up, and analysed	12<br>16-17
		(b) Give reasons for non-participation at each stage	
		(c) Consider use of a flow diagram	
Descriptive data	14*	(a) Give characteristics of study participants (eg demographic, clinical, social) and information on exposures and potential confounders	13
		(b) Indicate number of participants with missing data for each variable of interest	
		(c) *Cohort study*—Summarise follow-up time (eg, average and total amount)	
Outcome data	15*	*Cohort study*—Report numbers of outcome events or summary measures over time	
		Case-control study—Report numbers in each exposure category, or summary measures of exposure	
		Cross-sectional study—Report numbers of outcome events or summary measures	*13-14*
Main results	16	(*a*) Give unadjusted estimates and, if applicable, confounder-adjusted estimates and their precision (eg, 95% confidence interval). Make clear which confounders were adjusted for and why they were included	14-27
		(*b*) Report category boundaries when continuous variables were categorized	
		(*c*) If relevant, consider translating estimates of relative risk into absolute risk for a meaningful time period	
Other analyses	17	Report other analyses done—eg analyses of subgroups and interactions, and sensitivity analyses	15-16
**Discussion**			
Key results	18	Summarise key results with reference to study objectives	27
Limitations	19	Discuss limitations of the study, taking into account sources of potential bias or imprecision. Discuss both direction and magnitude of any potential bias	30
Interpretation	20	Give a cautious overall interpretation of results considering objectives, limitations, multiplicity of analyses, results from similar studies, and other relevant evidence	28
Generalisability	21	Discuss the generalisability (external validity) of the study results	30
**Other information**			
Funding	22	Give the source of funding and the role of the funders for the present study and, if applicable, for the original study on which the present article is based	2

*Give information separately for cases and controls in case-control studies and, if applicable, for exposed and unexposed groups in cohort and cross-sectional studies.

For peer review only

BMJ Open

Cross-sectional evaluation of an asynchronous Multiple Mini Interview (MMI) in selection to health professions training programmes with ten principles for fairness built-in

Journal:	*BMJ Open*
Manuscript ID	bmjopen-2023-074440.R1
Article Type:	Original research
Date Submitted by the Author:	21-Jul-2023
Complete List of Authors:	Callwood, Alison; University of Surrey Faculty of Health and Medical Sciences, FHMS<br>Harris, Jenny; University of Surrey Faculty of Health and Medical Sciences<br>Gillam, Lee; University of Surrey,<br>Roberts, Sarah; University of Surrey Faculty of Health and Medical Sciences<br>Kubacki, Angela; St George's University of London, IMBE<br>Christidis, Angelos; University of Surrey Faculty of Engineering and Physical Sciences<br>Tiffin, Paul; University of York, Department of Health Sciences
**Primary Subject Heading**:	Medical education and training
Secondary Subject Heading:	Nursing
Keywords:	MEDICAL EDUCATION & TRAINING, Nursing Care, Health Education

SCHOLARONE™ Manuscripts

I, the Submitting Author has the right to grant and does grant on behalf of all authors of the Work (as defined in the below author licence), an exclusive licence and/or a non-exclusive licence for contributions from authors who are: i) UK Crown employees; ii) where BMJ has agreed a CC-BY licence shall apply, and/or iii) in accordance with the terms applicable for US Federal Government officers or employees acting as part of their official duties; on a worldwide, perpetual, irrevocable, royalty-free basis to BMJ Publishing Group Ltd ("BMJ") its licensees and where the relevant Journal is co-owned by BMJ to the co-owners of the Journal, to publish the Work in this journal and any other BMJ products and to exploit all rights, as set out in our licence.

The Submitting Author accepts and understands that any supply made under these terms is made by BMJ to the Submitting Author unless you are acting as an employee on behalf of your employer or a postgraduate student of an affiliated institution which is paying any applicable article publishing charge ("APC") for Open Access articles. Where the Submitting Author wishes to make the Work available on an Open Access basis (and intends to pay the relevant APC), the terms of reuse of such Open Access shall be governed by a Creative Commons licence – details of these licences and which Creative Commons licence will apply to this Work are set out in our licence referred to above.

Other than as permitted in any relevant BMJ Author's Self Archiving Policies, I confirm this Work has not been accepted for publication elsewhere, is not being considered for publication elsewhere and does not duplicate material already published. I confirm all authors consent to publication of this Work and authorise the granting of this licence.

For peer review only

Cross-sectional evaluation of an asynchronous Multiple Mini Interview (MMI) in selection to health professions training programmes with ten principles for fairness built-in

Running head: evaluation of an asynchronous MMI

Corresponding author: Dr Alison Callwood Faculty of Health and Medical Sciences[1], University of Surrey, 30 Priestley Road, Guildford, Surrey, GU2 7YH, UK a.callwood@surrey.ac.uk. +44 7796956143

Dr Jenny Harris[1] jen.harris@surrey.ac.uk

Dr Lee Gillam[2] Faculty of Engineering and Physical Sciences, University of Surrey, Guildford, GU2 7XH, UK l.gillam@surrey.ac.uk

Mrs Sarah Roberts[1] sarah.roberts@surrey.ac.uk

Angela Kubacki[3] St George's University of London, London, UK akubacki@sgul.ac.uk

Angelos Christidis[2] Angelos.Christidis@surrey.ac.uk

Paul A Tiffin[4] University of York, York, UK paul.tiffin@york.ac.uk

Key Words

Multiple Mini Interview, equity, diversity and inclusion, fairness, asynchronous, online interviews

Word count 4371

For peer review only

Abstract

Objectives

We aimed to explore psychometric properties of the first known online asynchronous Multiple Mini-Interview (MMI) designed for fairness with sub-group analysis by key characteristics; usability, and acceptability.

Design: Cross-discipline multi-method evaluation

Setting: One United Kingdom University

Participants: Applicants to Nursing, Midwifery and Paramedic Science under-graduate programmes during 2021-2022.

Primary, secondary outcome measures

Psychometric properties (internal consistency, construct validity, dimensionality) were assessed using Cronbach's alpha (α), parallel analysis (PA), Schmid-Leiman transformation and ordinal confirmatory factor analysis (CFA). Usability and acceptability were evaluated using descriptive statistics and conventional content analysis.

Methods

The system was configured in a seven question four-minute MMI. Applicants' video-recorded their answers which were later assessed by interviewers and scores summed. Applicants and interviewers completed online evaluation questionnaires.

Results

Performance data from 712 applicants determined good-excellent reliability for the asynchronous MMI (mean α 0.72) with similar results across sub-groups (gender, age, disability/support needs, UK/non-UK). Parallel analysis and factor analysis results suggested there were seven factors relating to the MMI questions with an underlying general factor that explained the variance in observed candidate responses. A confirmatory factor analysis testing a seven-factor hierarchical model showed an excellent fit to the data (Confirmatory Fit Index =0.99), Tucker Lewis Index =0.99, RMSE=0.034).

For peer review only

Applicants (n=210) viewed the flexibility, relaxed environment, and cost savings advantageous. Interviewers (n=65) reported the system to be intuitive, flexible with >70% time saved compared to face-to-face interviews. Reduced personal communication was cited as the principle disadvantage.

Conclusions

We found that the asynchronous MMI was reliable, time-efficient, fair, and acceptable. These novel, low-cost insights are applicable across health professions selection internationally informing the future configuration of online interviews to ensure workforces represent the societies they serve.

Study strengths and limitations of this study

- The underpinning theoretical approach aligned with an iterative process necessary to design a new technology to reduce bias.
- The large sample enabled us to assess psychometric properties with sub-group analysis for the first time in this context.
- The study provides perspectives from one large site; a necessary step to inform a planned international multi-site evaluation.
- The multi-method design provided insights necessary to embed fairness into online selection approaches in the absence of best practice guidance.

For peer review only

Introduction

Ensuring equity, inclusivity, and diversity in selection to health professions training programmes is recognised internationally as an ethical and practical imperative [1,2]. Globalisation and increased workforce pressures amplify this need [3]. Fulfilling our responsibility to ensure fair selection is complex due to unintended biases that are intrinsic to human assessment compounded by recent unprecedented change to online interviews in the absence of published evidence [4,5,6,7]. Historically, health professions' selection has been mainly face-to-face using unstructured or structured approaches including panel interviews, group interviews, assessment centres and multiple mini-interviews (MMIs) [8]. MMIs are a series of short, focused interactions with a number of different interviewers. The multi-question format featuring structured scoring proforma with interviewers who have no prior knowledge of applicants, is designed to mitigate the potential impact of interviewer bias [9]. MMIs have been shown to be a feasible, acceptable, valid, and reliable candidate selection approach across health professions. None-the-less, as a face-to-face method, MMIs can be costly, resource intensive and influenced by unintended bias [10].

Online interviews were a relatively uncommon occurrence in selection to health professions before the pandemic. Approaches included Skype-based MMIs, asynchronous MMIs and asynchronous panel interviews [11]. Research outside the field of healthcare has shown asynchronous video interviews to be faster, cheaper, and require less employee time, easing scheduling burden and allowing for more applicants to be screened [12]. This can potentially increase the number of applicants who would have otherwise not had the opportunity to be interviewed.

During the pandemic, it was vital to ensure the continuance of recruitment to health professions. This resulted in rapid adaption to using online interviews facilitated by videoconference technology [13,14]. Recent research suggests online interviews like MMIs are feasible and acceptable provided reliable high-speed internet connection is available. However, access to reliable Wi-Fi is not always

For peer review only - http://bmjopen.bmj.com/site/about/guidelines.xhtml

possible [11]. In an asynchronous approach, applicants record their interviews at a convenient time and place, alleviating the stress of potential technical issues.

In live synchronous interviews, nuanced inconsistencies in, for example, tone and intonation, can arise in the way interviewers ask questions. Consistency of questioning across applicants is assured in asynchronous interviews through the use of pre-recorded interview questions. Fairness is further ensured with the avoidance of nonadherence to set questions which can occur in live interviews when applicants and interviewers serendipitously find something in common and deviate to discuss this [12,15]. Moreover, evidence from recruitment to the police force demonstrates that fairness can also be optimised through incorporating language that supports the affirmation of values with increased probability of minority applicants passing an assessment by 50% [16,17].

'Fairness' in this article is conceptualised as the quality of treating people equally or in a way that is right or reasonable. That encapsulates perceived fairness by participants as well as that borne out in the data. Consensus on the design of online interviews to optimise applicant accessibility and usability and mitigate potential unfairness issues for people across demographics, abilities, and disabilities is not readily available [15]. To address this, we successfully applied to Innovate UK, the United Kingdom's innovation agency (2020-2021) to build and evaluate what we believe was, the first (proof-of-concept, PoC) asynchronous videoconference facilitated interview and assessment system uniquely grounded in the MMI method (Figure 1).

Asynchronous MMI Development Summary

The asynchronous MMI is an on-demand videoconference interview where applicants log onto an online portal to complete their interview. The interview is a remote experience, where the system records applicants' short video responses to MMI questions in a timed process emulating the face-

For peer review only

to-face MMI method. There is no synchronous, bidirectional communication, and no interviewer to interact with; instead, questions are pre-recorded by interviewers. Applicants' recorded responses are scored by an interview assessor at a convenient later date.

To inform the design, we undertook a rapid review of literature published between 2011-2021, guided by a five-stage process [18, 19] to help us understand how video-based interviews compare with face-to-face in terms of implementation and fairness including what makes for an optimising experience (details provided in Appendix 1).

Indicative themes were elicited and assimilated into the following ten key principles and the system was re-configured accordingly:

- Recognise potential issues with stereotype threats and belonging uncertainty that may impact on candidates' performance and use language that supports the affirmation of values e.g., "well done for getting this far".
- Incorporate encouraging words/phrases into the interview dialogue, as well as any communications circulated to applicants (e.g., "good luck").
- Soften the language of technical instructions e.g., "when you are ready …" or "when you have familiarised yourself with…".
- Reduce the verbal load of interview content particularly for neurodivergent applicants.
- Accommodate access and engagement for neurodivergent applicants with extra time, adjusted fonts and a tailored user-interface (UI) including background colours.
- Provide opportunities for candidates to familiarise themselves with the UI and format prior to their interview though a practice portal.
- Recommend generic, blank backgrounds for video or videoconference facilitated interviews or, if not possible, advise blurred backdrops.

For peer review only - http://bmjopen.bmj.com/site/about/guidelines.xhtml

- Ensure diversity of interviewers for pre-recorded videos and those assessing them including gender, age and ethnicity, experts-by-experience, and other stakeholders.
- Avoid culturally sensitive subject areas, language, age and ability bias in interview content.
- Ensure the use of inclusive, gender-neutral language with appropriate pronouns e.g., 'they/them/their'.

The focus of the article is to detail the study undertaken to evaluate the psychometric properties of the asynchronous MMI. We aimed to explore reliability (internal consistency) for all users with sub-group analysis by key characteristics (age, gender, nationality, disability/additional support needs), construct validity, dimensionality, acceptability, and usability.

In this context disability refers to a person who self-identifies with physical or mental impairment, and the impairment has a substantial and long-term adverse effect on the person's ability to carry out normal day-to-day activities. Not all neurodivergent people consider themselves 'disabled' but instead neurological conditions are viewed a result of normal variations in the human genome but where additional support might be required. We therefore use the term 'support need' to include neurodiverse applicants.

Methods

The MMI questions were developed and tested by the university's community emulating our previously established in-person processes. A diverse range of individuals including academic staff, service users and practice partners were individually videorecorded asking MMI questions. The video recordings were uploaded onto the system, which was configured in a seven question, four-minute MMI with one minute between questions.

The asynchronous online MMI system was adopted for selection to Health Professions undergraduate programmes (Adult, Child, Mental Health Nursing, Midwifery, and Paramedic Science) at one UK

For peer review only - http://bmjopen.bmj.com/site/about/guidelines.xhtml

university during 2021/22 recruitment cycle. All applicants to these programmes were invited to register for a one-week slot that was convenient for them. At the start of their selected slot, applicants were emailed a link to the system. Here they could access the practice portal to check their Wi-Fi speed, familiarise themselves with the user interface/key functionality, become conversant with the process through a detailed instructional video, and practice a question. When they felt prepared, they could start their MMI. To mirror our previous in-person process, once they had begun, applicants could not stop their MMI and start again. Any technical issues including those without the necessary equipment and/or Wi-Fi connectivity were supported to enable their successful completion on an individual basis.

All individuals at the university who would usually interview applicants as part of their role took part in this new process. These included academic staff, service users and practice partners. They were invited to self-allocate to one-week interview assessment slots. Thereafter they received a link and code to access the system to review and assess applicant interviews against uploaded scoring rubrics live on the system. Each interviewer was allocated a number of applicant's video responses to a single question thereby aligning with the principles of an MMI. Also, emulating MMI methodology, a 'red flag' option could be ticked, and details populated in a text box if a cause for concern was raised in applicants' answers. Applicant's performance scores were downloaded at the end of the process to inform offer/reject decisions by university Admissions Officers.

University Admissions Officers conducted an internal resource evaluation to explore time and cost spent on the asynchronous MMI compared with face-to-face and Zoom-facilitated MMIs.

Design

This study was underpinned by Olsen and Eoyang's theory of 'Complex Adaptive Systems' [20]. This is characterised by an adaptive and iterative working style appropriate when developing and optimising

For peer review only - http://bmjopen.bmj.com/site/about/guidelines.xhtml

the system for fairness and reliability in the absence of any known precedent. We also grounded the system design in Gilliland's [21] justice-based model. According to their theory, a selection system's adherence to procedural and distributive justice rules promotes applicants' perceptions of fairness. Procedural justice rules relate to the approach used to derive decisions, in this case an asynchronous MMIs. It includes the formal process (opportunity to perform and administration), explanation (feedback, information, and transparency), and interpersonal communication throughout the selection process. Distributive justice rules encompass adherence to equity when determining selection outcomes [21].

Participant recruitment

All applicants (n=712 at the data collection point) to the UK university and all interviewers (n=96) who assessed applicants were invited to evaluate the system.

Patient and Public Involvement

The university's service user group were supportive of the move to online asynchronous interviews. One service user acted as an interviewer by videorecording an MMI question. A further three were interview assessors, thereby continuing an established model at this university of service user involvement in recruitment. The service user group were also involved in the MMI question writing by reviewing draft questions and providing feedback. In the seven-question circuit, one practice partner video recorded an MMI question and eight assessed applicants' videos.

Data Collection

Applicant interview performance data, routinely collected to inform offer/reject decisions, were used in the reliability analyses. Applicant interviews were scored against 10 question-specific criteria on a seven-point Likert-type scale with descriptive anchors for the seven questions. Criteria details are withheld for test security. Applicant interview scores were summed for each applicant.

For peer review only - http://bmjopen.bmj.com/site/about/guidelines.xhtml

To assess usability, applicants were invited to complete an online semi-structured evaluation questionnaire hosted on Qualtrics[22] once they had received their interview outcome decision.

To assess acceptability, interviewers were invited to evaluate the process at the end of the recruitment cycle through an online semi-structured questionnaire also hosted on Qualtrics [22].

Analysis

We used applicant scores across questions to explore reliability (internal consistency) for all users and a randomly selected sub-sample self-reporting key characteristic including gender, age (<20 or 20+), nationality (UK/non-UK) and disability/support needs (absence/presence) using Cronbach's alpha. (Stata, version 16.1, StataCorp LLC) [23]. Cronbach's alpha, as a conventional measure of internal-consistency (reliability) assumes that item responses are unidimensional, meaning that they are reflective of only one underlying construct or factor. However, this may not always be the case therefore scale dimensionality and was assessed using a parallel analysis and confirmatory factor analysis (CFA)[24,25]. The parallel analysis and reliability values were derived using the software package FACTOR [26]. The ordinal factor analyses were conducted in Mplus [27] version 8.8. Usability and acceptability were explored with descriptive statistics (closed questions) and conventional content analysis [28] (open questions).

Results

Sample Characteristics

Data were available from 712 applicants to Nursing, Midwifery and Paramedic Science programmes at the data collection point on 1st May 2022 and for key characteristics for the sub-sample (n= 284) (Table 1). Disabilities self-reported by interviewers were reduced hearing and visual acuity. Applicants reported neurodivergent challenges across the spectrum of dyslexia, dyspraxia, and attention deficit hyperactivity disorder (ADHD).

For peer review only - http://bmjopen.bmj.com/site/about/guidelines.xhtml

Table 1: Participant self-identified characteristics

Interview Assessors n= 65		N	%	Applicant Sub-sample n=284	N	%
Age (years)	25 and under	0	0	<20	193	68
	26-35	8	13			
	36-45	18	28	>20	91	32
	45+	35	53			
	Prefer not to say	4	6			
Gender	Female	55	84	Female	230	81
	Male	7	10	Male	54	19
	Other	0	0	Other	0	0
	Prefer not to say	3	6	Prefer not to say	0	0
Disability/additional support needs	No	60	93	No	228	80
	Yes	5	7	Yes (64% neurodiverse, 34% mental health, 2% hearing challenges)	58	20
	Prefer not to say	0	0	Prefer not to say	0	0
Ethnicity	White (English/Welsh/Scottish/Northern Irish, Irish, Gypsy, Irish Traveller, Other White background)	61	94	UK/Ireland	229	81
	Mixed/multiple Ethnic Groups (White and Black Caribbean, White and Black African, Any other Mixed Background)	2	3	Non-UK/Ireland	55	19

For peer review only

For peer review only - http://bmjopen.bmj.com/site/about/guidelines.xhtml

	Asian/Asian British (Indian, Pakistani, Bangladeshi, Chinese, Any other Asian background)	0	0		N/A	N/A
	Other (Arab, any other ethnic group)	0	0			
	Prefer not to say	2	3			
Role	University Health Sciences Staff	46	71		N/A	N/A
	Practice Partner	10	16			
	Service user	9	13			
	Prefer not to say	0	0			

Reliability and assessment of fairness

Reliability

Applicant data were shown to be normally distributed (Kurtosis 0.000, p 0.005) and symmetrical with skewness 1.0.

Internal consistency was good-excellent across scenario questions (for n=712 applicants) (mean Cronbach's α 0.72 (range 0.64-0.89). Sub-group analyses showed similarly positive results with mean α Female/Male: 0.74/0.87 (range 0.70-0.89); age: <20years/ >21 years 0.76/0.83 (range 0.72-0.86), disability/additional challenges/non-disability: 0.88/0.78 (range 0.74-0.89) and UK/non-UK 0.78/0.0.77 (range 0.72-0.83) (Table 2).

Dimensionality/construct validity

The results of the parallel analysis suggested a maximum of seven dimensions underlay the response pattern. A Schmid-Leiman [29] transformation can be used to understand if a factor analytic model is best understood as a hierarchical in nature. This partitions observed variance into that explained by one or more general ('G') factors underlying three or more specific factors (a hierarchical model will be mathematically 'just identified' by three factors) [29]. The results of the Schmid-Leiman

For peer review only

transformed factor analysis indicated a seven-factor solution, relating to the MMI scenarios, with an underlying general factor which substantially loaded on all the former seven factors. An ordinal Confirmatory Factor Analysis (CFA) to test this seven-factor hierarchical model showed an excellent fit to the data (Confirmatory Fit Index (CFI)=0.99, Tucker Lewis Index (TLI)=0.99, RMSE=0.034). In contrast, a one factor model showed a poor fit to the response data (CFI=0.70, TLI=0.69, RMSEA=0.19) (Appendix 2)

The results of the parallel analysis suggested a maximum number of seven plausible factors. The variance explained by additional postulated factors did not exceed that observed for the random data generated. (Appendix 3).

Table 2 MMI question reliability (internal consistency) N=712

Question (total mean)	Obs	Mean	St Dev	Min	Max	Item-test correlation	Item-rest correlation	Cronbach's alpha
1	765	48.6732	10.3966	10	70	0.6541	0.4677	0.68
2	795	51.57107	9.70229	10	70	0.6434	0.4532	0.67
3	790	51.08101	9.10597	10	70	0.599	0.421	0.69
4	809	50.23486	8.97655	10	70	0.5826	0.4001	0.70
5	762	50.27428	9.88047	10	70	0.6624	0.4941	0.67
6	765	51.79869	8.7486	20	70	0.6132	0.4484	0.69
7	712	50.10499	11.0865	14	70	0.6076	0.3871	0.70
Scale reliability coefficient								0.7216

For peer review only

Sub-group reliability n=284								
Question (total score)	Cronbach's alpha							
	Age		Self-identified gender		Self-declared disability		Country of birth	
	< 20 years	> 20 years	Female	Male	Yes	Non	UK/Ireland	Non-UK/Ireland
1	0.74	0.83	0.73	0.87	0.88	0.76	0.78	0.74
2	0.74	0.83	0.72	0.87	0.88	0.76	0.77	0.75
3	0.75	0.83	0.74	0.87	0.87	0.77	0.78	0.77
4	0.77	0.85	0.76	0.89	0.88	0.79	0.79	0.79
5	0.73	0.82	0.71	0.86	0.87	0.75	0.76	0.73
6	0.77	0.84	0.76	0.87	0.87	0.79	0.79	0.79
7	0.79	0.87	0.78	0.88	0.89	0.80	0.80	0.86
Mean	0.76	0.83	0.74	0.87	0.88	0.78	0.78	0.77

Usability (applicants)

For peer review only - http://bmjopen.bmj.com/site/about/guidelines.xhtml

The online evaluation was completed by 210 applicants (29% response rate). The majority were under 20 years of age, self-identified as white female and with representation from across programmes (Table 3).

Table 3 Applicant Characteristics and usability evaluation

N=210			
Demographics		N	%
Age	<20	120	57
	20+	90	43
Self-identified gender	Female	172	82
	Male	36	17
	Other/non-binary	2	1
Nationality	UK/Ireland	134	64
	Non-UK/ Ireland	68	32
	Prefer not to say	8	4
Programme representation			
Nursing	Adult	42	20
	Child	34	16
	Mental Health	52	25
Midwifery		32	15
Paramedic Science		50	24
Closed question responses summary data			
Had not taken an asynchronous MMI like this before			94%
Found the instructions helpful/very helpful			90%
Did not experience technical issues			79%

For peer review only - http://bmjopen.bmj.com/site/about/guidelines.xhtml

Said the one minute between questions was 'about right'	75%
Found the probe questions helpful/very helpful	66%
Four minutes was about the right amount of time	65%

Open question free text responses				
Question	Response %			
How do you feel about videorecording and uploading your MMI responses as part of a 'new look' interview process triggered by Covid-19 social distancing restrictions?	Happy/very happy	67	Not very happy/not at all happy	33
Please tell us about your views on online interviews generally	Accepting/very accepting	66	Partly accepting/ Not at all accepting	35
Do you see a future for online interviews like the one you have used with us?	Yes/definitely yes	68	Probably not/Definitely not	32

The majority of applicants had not undertaken an asynchronous MMI previously. Overall applicants found the instructions either helpful or very helpful and did not experience any technical issues. From a setup perspective, four minutes was considered the right amount of time by over half with one-third stating it was too short. We asked additional overarching open free text questions regarding applicants' views on online interviews (Table 3) as well as their overall 'top' positives and 'top' negatives of system (Table 4 summary, Supplementary Table 1).

For peer review only

We received 158 separate positive comments and 140 negative comments in relation to the questions asking for 'top' positives and 'top' negatives, Notably, 93% of the positive comments centred around three themes: Ease (40%), reduced stress (28%) and fair (25%). We received fewer negative comments overall and these were split into two main themes: limited direct communication (34%) and critique of the MMI process itself (32%). Nineteen percent of respondents raised technical issues as a potential negative issue. However, it appears that as around 80% did not experience these in reality. The twelve respondents that did experience technical issues cited: buffering (3), frozen screen (2), crash (2), video skipping (2), screen scaling (1), microphone (1) and upload (1). They all subsequently successfully completed their interview at a second attempt.

For peer review only

Table 4 Summary Applicant and interviewer top positives and negatives

Applicant				
**Positives**				
Theme	Sub theme detail	N=158 (%)	% of total comments	Illustrative quote
Ease	Access, intuitive, convenient, flexible, simple	63 (40)	21	*"Quick, not time consuming, simple"*
Reduced stress	More relaxed in own home, take my time, breaks available, start when wish	45 (28)	15	*"It's more relaxing to be in your own home instead of a new environment which for me is much less intimidating resulting in perhaps me performing better in the interview".*
Fairer	Reduced costs (travel), reduced time away from other responsibilities (caring), the practice portal and availability of question text helped neurodiverse applicants	39 (25)	13	*"It's much easier in regard to travel for those who live far away, don't have the funds or time or who have other commitments".* *"Reduces the possibility of bias during the interview process".* *"...I could do a practice question to get used to the layout of the interview, so I wasn't going into the interview completely blind".*
'Meet' staff	Able to see more of the university's community	9 (7)	3	*"I liked that there were 7 different people asking the questions..."*
Covid safe	Travel not required	2 (1)	1	*"...Has helped to continue the process of admissions in a positive way..."*
**Negatives**				
Limited direct communication	Less personal, no conversation	48 (34)	16	*"... Impersonal, I feel like I can't make a connection or read the interviewers body language in the interview..."*
MMI	Pressure felt due to the timed methodology, presence of the countdown timer	45 (32)	15	*"4 minutes per question was quite pressuring to fill".* *"Ticking down time was off-putting".*
Technical issues	Wi-Fi cut out	27 (19)	9	"My *screen froze, potential upload failure, loss of connection".*
Don't get staff vibe	Not directly meeting staff	2 (1)	1	*"Lack of interaction between the student-tutor, the dialogue"*
Cannot ask questions		18 (13)	6	*"Not personalised, not able to ask questions"*

For peer review only - http://bmjopen.bmj.com/site/about/guidelines.xhtml

Interviewer				
Positives				
Convenient	Can prioritise workload. Flexible. Quicker<br>Less stressful	82 (62%)	37	*"Efficient, less time consuming and able to prioritise your workload and complete interviews in chunks as opposed to one long stint"*
Fairer	Less unconscious bias<br>Reduced travel [costs]	18 (14%)	7	*"Reduced bias from different people asking the questions".*<br>*"Equity... no student gets help with prompts more than others"*
East to navigate	Simple	12 (9%)	5	*"Easy to use with clear instructions"*
Benefits applicants	Less stressed in own environment	12 (9%)	6	*"Separating the two events [interview/applicant day] will help manage anxiety and stress"*
Applicant assessment	Can get a [better] sense of the applicant	4 (3%)	2	*"I feel I can get a sense of the applicant through this process".*
Less worried about technical issues	Not one time/date dependent	3 (2%)	2	*"Much less stressful as I don't have to worry about the internet connection".*
Ability to rewatch	If cause for concern'	1 (1%)	1	*"Ability to rewatch for clarification".*
Negatives				
Less personal	Don't get feel for applicant	27 (30%)	12	*"I think it's better for applicants to speak to someone in person".*<br>*"I question whether it produces the same quality of response".*
Critique of MMI methodology	Timed circuit/countdown timer	22 (24%)	10	*"Encourages to me to make a snap decision".*<br>*"Can't ask follow-on questions...permits no probing".*
Communication/building rapport difficult	Less able to assess non-verbal communication, social skills, spontaneous cognitive ability.	21 (23%)	9	*"Little in the way of holism".*<br>*"Disconnect perhaps for applicants".*
Limited support for nervous applicants	Cannot help if upset or stressed	8 (9%)	4	*"No opportunity to provide any support if the candidate appears upset.. if they are struggling mentally or emotionally"..*
Tech related process concerns	Tech issues<br>Stress of Zoom/Teams online mode	4 (5%)	2	*"Robotic process"*<br>*"Repetitive and boring watching multiple videos"*<br>*"Technical issues for some student may give them a disadvantage".*
Did not have any	Stated 'none'	5 (6%)	2	*"Don't have any"*

For peer review only - http://bmjopen.bmj.com/site/about/guidelines.xhtml

Acceptability (Interviewers)

Sixty-five interviewers took part in the online evaluation representing a 71% response rate. The majority were white British female university staff, over the age of 45 years with no declared disability (Table 1). This is representative of the University Faculty staff profile which is located in the Southeast of the UK.

Ninety-six percent (n=62) of interviewers found the system intuitive, easy to use and reported a perceived reduction in their stress. They primarily attributed this to increased convenience and flexibility. A 70%-time reduction was independently reported by our Admissions Officers. They estimated this based on the time spent on other interview approaches (face-to-face MMIs, Zoom-facilitated MMIs compared with our asynchronous MMIs; categorised into pre-interview communications, set up, staff recruitment (including covering sick time), interview facilitation and post-interview communications. The majority of the time saving was ascribed directly to the asynchronous modality which removed the need for staff to either facilitate face-to-face or online live interviews. Additionally, the asynchronous approach alleviated the pressures of last-minute non-availability of interviewers particularly practice partners as they were not tied to one scheduled day/time but had a time period (1 week) within which they could assess the interview recordings.

None of the interviewer assessors stated that they had used an asynchronous online MMI previously. Almost all (96%) found it easy to use and the user interface intuitive (92%). Less than 10% reported technical issues other than download issues which were resolved. Five percent were 'not accepting' of using the asynchronous MMI in the future.

We were interested to better understand whether interviewers felt communication could be assessed in an asynchronous modality. Thirty-three percent said 'yes', 54% 'somewhat' with 13% of respondents (n=6) responded 'no'. To generate more in depth insights we asked interviewers their top positives and negatives of the system. These are presented in Table 4. We received 132 positive and 90 negative comments. The majority of positive comments (94%) related to perceived

For peer review only - http://bmjopen.bmj.com/site/about/guidelines.xhtml

convenience (62%), fairness (14%) ease of navigation (9%) and benefits for the applicant (9%). Negative comments were split more evenly into perceptions of their being less personal (30%), critique of MMI methodology (24%), limited communication assessment and ability to build rapport (23%), and 5% had technology-related process concerns. Six percent cited 'none'.

Discussion

With ten principles for fairness designed-in these findings suggest the online interview is reliable, fair, time-efficient, and acceptable. The results of the factor analyses infer that there are scenario-level effects but that that these all relate to an underlying general factor indicative that the process is assessing different dimensions/constructs relevant to health care. These could be method effects or alternatively conceptualised as representing different aspects of the interpersonal procedural knowledge required to perform well on the MMI.

This platform is the only known custom built asynchronous online interview emulating the MMI methodology. Cognisant of Gilliland's [21] procedural and distributive justice rules, our aim was to optimise applicant accessibility through building principles for fairness into the MMI design and system set up. The reliability results and usability and acceptability evaluation signal this was largely achieved which is critical to applicants perception of fairness [30]. These data suggest the configuration of our asynchronous MMI resulted in an equitable process particularly with the familiarisation enabled through the practice portal [15]. We note the higher Cronbach's alpha for disability compared with non-disability. While this is reassuring, it merits further investigation with a larger sample size.

Rice [31] (p452) suggests that social presence or the "degree to which a medium is perceived as conveying the presence of the communicating participants" impacts on applicant acceptability. Social presence plays a central role in trust, enjoyment, and the perceived usefulness of the technological

For peer review only - http://bmjopen.bmj.com/site/about/guidelines.xhtml

medium [30]. There was by definition an absence of actual social presence in the asynchronous modality. However, we sought to mitigate this through softening the intersection between human and technology though design. The majority said they found the system intuitive/very intuitive and simple to use. This infers that the user interface design and inclusive language may have contributed towards a positive experience.

Applicants in this study (>66%) were either accepting or very accepting of the online asynchronous MMI with around one third (37%) agreeing we should 'definitely return' to face-to-face interviews. Notably, only 2% (n= 2) of applicants said they were 'not at all accepting' of the asynchronous MMI. The majority of applicants were 20 years or under. It could be suggested that a younger demographic are more familiar with and accepting of online technology and see it as part of their day-to-day lives. However, these data signal that there was no difference in reliability for those under 20 years compared to over 20 years old and that applicant performance was not impacted by their age.

Applicants' experiences, particularly perceptions of fairness, are of paramount concern for universities. The implications of fairness can extend to post-interview outcomes including offer/acceptance rates. It has been suggested that applicants who perceive that recruitment and selection processes are fair are more attracted to organisations [32,33,34]. Concurring with Brenner et al [12] applicants reported 'perceived fairness' as one of their top three positives of the asynchronous interview system. Their reasons include reduced travel costs and time away from caring responsibilities, as well as enhanced familiarisation of the process through the practice portal. MMI interviews were pre-recorded using inclusive language by diverse staff, representative of the University community. Additional time and an intuitive system user interface appeared to help meet the needs of neurodiverse applicants.

Incorporating the ten fairness principles were not difficult as many were low-cost design features that appeared to be impactful and generically applicable. We suggest these should become a default

For peer review only - http://bmjopen.bmj.com/site/about/guidelines.xhtml

approach for online interviews used in health professional selection to enable applicant performance optimisation. In view of the paucity of published evidence [15] these novel insights are informative as we inevitably move toward a technology-augmented future where asynchronous video interviews are considered a modality that is here to stay [15].

We received a 60:40 ratio of positive to negative comments from interview assessors. The largest contributor (62%) to the overall feedback related to positivity around convenience including ability to prioritise workload, flexibility, speed, and reduced stress. This was followed by ease of navigation and reduced bias. These findings corroborate evidence garnered outside the field of healthcare where asynchronous interviews have been found to be faster, cheaper, require less employee time and open the applicant funnel to allow more people to be interviewed than would otherwise have had the opportunity[15]. Nevertheless, communication skills are central to the role of a health professional and are assessed as a generic skill/attribute in each MMI question at this university. One sixth of interview assessors said they did not feel communication skills could be assessed while over a third stated they could in the asynchronous modality. Further research is warranted to better understand the intersection between humans and technology including barriers and enablers to effective communication and communication assessment.

The largest contributing negative comment (30%) focused on a perception that the process was less personal. We might have anticipated this to be higher. Steps being considered to enhance personal connection include live chats and Q&A sessions as well as increasing the number of 'offer holder-days' provided by the university where applicants are invited onto campus to engage with staff without the stress of an interview clouding the experience. Further research evaluating the effectiveness of this strategy is suggested.

Strengths and limitations of this study

For peer review only - http://bmjopen.bmj.com/site/about/guidelines.xhtml

Invitations to evaluate the system were sent out after applicants received notification of their interview outcome. We understand that the interview outcome may have impacted on applicants' perception of the process. In this study, we were required to adhere to the university's preference. This will be reviewed in the design of any future study to minimise sampling and response bias.

The study was a theoretically driven mixed-methods approach with multi-programme perspectives offering important insights. However, we acknowledge the generalisability limitations of a single site design, but this was an essential step ahead of a planned large multi-site international evaluation.

While the sample size is large, the low response rate from applicants is a potential limitation and may infer selection bias. Users' views of such a technology can be impacted by many factors outside the scope of this research for example past experiences for which we were unable to account for.

It was not possible to conduct a comparison study 'pre/post system optimisation'. Covid necessitated a move to online interviews in unprecedented times. Data were not collected on applicant or interviewer views at the time given the burdens they were already facing. During that time however, we explored how fairness could be optimised through a review of published literature with findings embedded in our system (Appendix 1). In a high stakes admission process, it would be ethically wrong to conduct a prospective study now to compare with and without the 10 principles for fairness given the apparent benefits. Data is not available retrospectively due to the circumstances around Covid.

Collapsing applicant data into UK/Ireland and Non-UK Ireland was a necessary pragmatic decision based on lack of consistent reporting of ethnicity between the university (who did not routinely retain applicant ethnicity data until enrolment) and the UK University Central Admissions System (UCAS).

Rigour

In spite of reassurances in all communications, we were mindful that applicants might be concerned that their review of the interview process might impact on their interview outcome hence the invitation to evaluate once offer/reject decisions had been communicated to applicants.

For peer review only - http://bmjopen.bmj.com/site/about/guidelines.xhtml

All data in this evaluation were independently analysed and peer reviewed by multiple authors (AC, JH, SR, PT). In the qualitative content analysis, two authors (AC, SR) undertook the analysis independently. A <5% difference was noted between authors' findings. A compromise was mutually agreed in instances where this occurred.

Conclusion

With ten principles for fairness designed in, these findings suggest the asynchronous online interview is reliable, equitable, time-efficient, and acceptable. It is a moral imperative that healthcare workforces represent the societies which they serve, however unintended bias can influence selection decisions. In the absence of generically available consensus guidance on how fairness can be optimised in online interviews, these novel insights are applicable internationally across selection to health professions. Embedding fairness into the design of online interviews is relatively straightforward and low cost to implement. These data advance our understanding which is vital as we inevitably more towards a technology augmented future in the context of global workforce pressures.

Figure 1: Asynchronous MMI infographic.

For peer review only

Contributor statement

Author AC is guarantor. Authors AC, LG and Ach contributed to the technical development. Authors AC, LG, Ach, JH, PT, AK, SR contributed to the study design, data collection, drafting and revisions. Authors AC, JH, SR, PT contributed to the data analysis. Author Ach is no longer directly affiliated to the university but was at the commencement of this study.

Competing interests statement

Authors AC and LG are co-founders and Ach is an employee of Sammi-Select Lt, funded by UKRI as a spinout company from the University of Surrey to build the platform.

Funding

This work was supported with funding from Innovate UK grant number 37043.

Data sharing statement

Data sets used and/or analysed are available on reasonable request. Detailed technical information is withheld due to commercial sensitivity.

Ethics statement

This study received a favourable ethical opinion (FEO) from the University Research Ethics committee (UEC/2017/111/FHMS). Participants gave informed consent where personal data was included.

Acknowledgements

Grateful thanks to staff, students, practice partners and service users without whom this work would not be possible.

For peer review only

References

1. Council of Deans of Health Equality, Diversity, and Inclusion: https://www.councilofdeans.org.uk/category/policy/equality-diversity-and-inclusion/. Accessed 06.03.23.
2. United Nations Department of Economic and Social Affairs 17 Sustainable Development Goals: https://sdgs.un.org/goals. Accessed 06.03.23.
3. https://www.oecd.org/publications/the-post-covid-19-rise-in-labour-shortages-e60c2d1c-en.htm
4. Sabesan V, Kapur N, Zemanek K, Levitt D, Vu T, Erp A. Implementation and evaluation of virtual multiple mini-interviews as a selection tool for entry into paediatric postgraduate training: A Queensland experience. Medical Teacher Published online: 30 Aug 2021. https://doi.org/10.1080/0142159X.2021.1967906
5. Cleland J, Chu J, Lim S, Low J, Lowe-Beer N, Kwek T. COVID-19: designing and conducting an on-line mini-multiple interview (MMI) in a dynamic landscape. Medical Teacher. 2020. May: 1-5. https://doi.org/10.1080/0142159X.2020.1762851.
6. Kok K, Chen L, Idris F, Mumin N, Ghani H, Zulkipli. Conducting multiple mini-interviews in the midst of COVID-19 pandemic. Korean J Med Educ. 2020 Dec; 32(4): 281–289. Published online 2020 Oct 28. doi: 10.3946/kjme.2020.175.
7. Fuller, R., Goddard, V., Nadarajah, V., Treasure-Jones, T. Yeates, P., Scott, K., Webb, A., Valter, K., Pyorala, E. 2022. Technology enhanced assessment: Ottawa consensus statement and Recommendations. Medical Teacher Pages 836-850 https://doi.org/10.1080/0142159X.2022.2083489
8. Patterson, F, Roberts, C, Hanson, M, Hampe, W, Eva, K, Ponnamperuma, G, Magzoub, M, Tekianhand, A, Cleland, J. Ottawa consensus statement: Selection and recruitment to the

For peer review only

healthcare professions. Medical Teacher. 2018;40(11):1-11. 10.1080/0142159X.2018.1498589

9. Eva K, Rosenfeld J, Reiter H, Norman G. An admissions OSCE: the multiple mini-interview. *Medical Education*. 2004; 38: 314-326.
10. Yusoff J. Multiple Mini Interview as an admission tool in higher education: Insights from a systematic review. J Taibah Univ Med Sci. 2019 Jun; 14(3): 203–240. doi: 10.1016/j.jtumed.2019.03.006
11. Selvam R, Hu R, Musselman R, Raiche I, Mclsaac, D, Moloo H (2021) Video-based interviewing in Medicine: A Scoping Review. Doi: https://doi.org/10.21203/rs.3.rs-468538/v1
12. Brenner, F. S., Ortner, T. M., & Fay, D. (2016). Asynchronous video interviewing as a new technology in personnel selection: The applicant's point of view. *Frontiers in Psychology, 7*, 863.
13. Huppert L A, Hsiao E C, Cho K C *et al.* Virtual interviews at graduate medical education training programs determining evidence-based best practices. *Acad Med* 2020. doi: 10.1097/ACM.0000000000003868.
14. Vining CC, Eng OS, Hogg ME, et al. Virtual Surgical Fellowship Recruitment During COVID-19 and Its Implications for Resident/Fellow Recruitment in the Future. Ann Surg Oncol. 2020;27(Suppl 3):911- 915. doi:10.1245/s10434-020-08623-2
15. Lukacik, E., Bourdage, J., Roulin, N. 2022. Into the void: A conceptual model and research agenda for the design and use of asynchronous video interviews. Human Resources Management Review 32 , 100789
16. Linos E, Reinhard J, Ruda S. Levelling the playing field in police recruitment: Evidence from a field experiment on test performance. *Public Administration*. 2017;95(4):943-956.
17. https://www.linkedin.com/business/talent/blog/future-of-recruiting

For peer review only - http://bmjopen.bmj.com/site/about/guidelines.xhtml

18. Bambra, C., Joyce, K., Bellis, M., Greatley, A., Greengross, S., Hughes, S., Lincoln, P. Lobstein, T., Naylor, C., Salay, R. Wiseman, M., Maryon-Davis, A. 2010 Reducing health inequalities in priority public health conditions: using rapid review to develop proposals for evidence-based policy. *Journal of Public Health*, Volume 32, Issue 4, December 2010, Pages 496–505, https://doi.org/10.1093/pubmed/fdq028

19. http://mixedmethodsappraisaltoolpublic.pbworks.com/w/file/fetch/127916259/MMAT_2018_criteria-manual_2018-08-01_ENG.pdf

20. Olsen E, Eoyang G. Facilitating organisational change: lessons from complexity science. San Francisco: Jossey-Bass; 2001.

21. Gilliland, S. 1983 The perceived fairness of selection systems: an organisational justice perspctive. Academy of Management Review Vol 18, No 4, pp 694-734

22. Qualtrics accesible at: https://www.qualtrics.com/uk/

23. Stata accessible at: https://www.stata.com/

24. Horn, J. L. (1965). A Rationale and Test for the Number of Factors in Factor Analysis. Psychometrika, 30, 179-185. https://doi.org/10.1007/BF02289447

25. Timmerman, M. E., & Lorenzo-Seva, U. (2011). Dimensionality assessment of ordered polytomous items with parallel analysis. *Psychological Methods, 16*(2), 209–220. https://doi.org/10.1037/a0023353

26. Lorenzo-Seva, U., Ferrando, P.J., 2006. FACTOR: a computer program to fit the exploratory factor analysis model. Behavior Research Methods 38, 88–91.

27. MPlus version 8. Los Angeles, CA: Muthén & Muthén, 2022

For peer review only - http://bmjopen.bmj.com/site/about/guidelines.xhtml

28. Hsieh H, Shannon S. Three Approaches to Qualitative Content Analysis. Qualitative Health Research. November.2005. http:// doi.org/10.1177/1049732305276687

29. Schmid, J., & Leiman, J. M. (1957). The development of hierarchical factor solutions. *Psychometrika*, *22*(1), 53–61.

30. Schinkel, S., van Vianen, A., & Van Dierendonck, D. (2013). Selection fairness and outcomes: A field study of interactive effects on applicant reactions. *International Journal of Selection and Assessment, 21*(1), 22–31.

31. Rice, R. E. (1993). Media appropriateness: Using social presence theory to compare traditional and new organizational media. *Human Communication Research, 19*(4), 451–484.

32. Oh, C. S., Bailenson, J. N., & Welch, G. F. (2018). A systematic review of social presence: Definition, antecedents, and implications. *Frontiers in Robotics and AI, 5*, 114.

33. Chapman, D. S., Uggerslev, K. L., Carroll, S. A., Piasentin, K. A., & Jones, D. A. (2005). Applicant attraction to organizations and job choice: A meta-analytic review of the correlates of recruiting outcomes. *Journal of Applied Psychology, 90*(5), 928–944. Chapman, D. S., Uggerslev, K. L., & Webster, J. (2003). Applicant reactions to face-to-face and technology

34. Truxillo, D. M., Bauer, T. N., Campion, M. A., & Paronto, M. E. (2002). Selection fairness information and applicant reactions: A longitudinal field study. *Journal of Applied Psychology, 87*(6), 1020–1031.

For peer review only

For peer review only

For peer review only - http://bmjopen.bmj.com/site/about/guidelines.xhtml

Figure 1: Asynchronous MMI infographic.

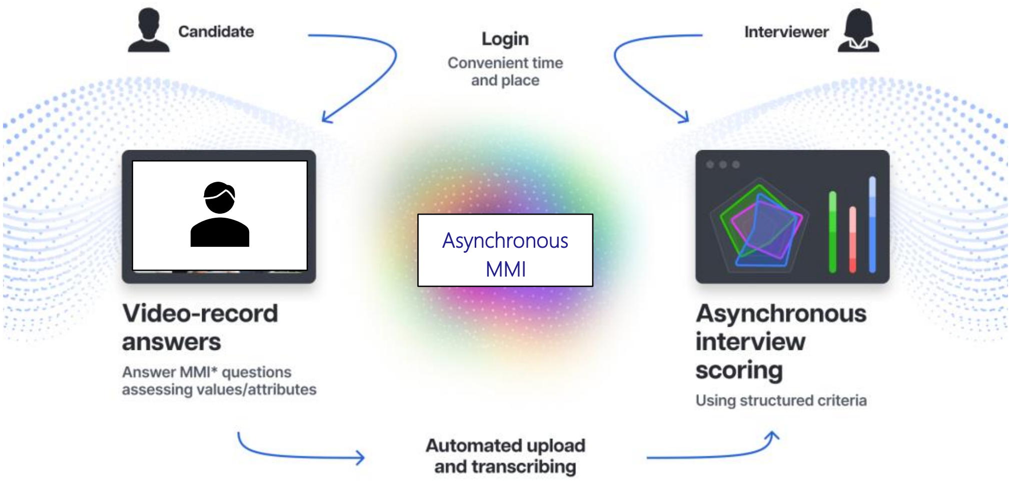

For peer review only

Appendix 1 Rapid Review Key References in Health Professions Selection

Rapid review of published literature

To inform the design, we undertook a rapid review of literature published between 2011-2021, guided by a five-stage process[18] to help us understand how video-based interviews compare with face-to-face in terms of implementation and fairness including what makes for an optimised experience.

The databases searched included: Medline, PsycInfo, Embase, ProQuest (BNI) and Google Scholar/Google (grey literature). We included original articles published in English that reported studies involving candidates interviewed using video with/without face-to-face methods in health professions selection. Articles focusing on psychometric tests and situational judgement tests were excluded. For quality appraisal we used the Mixed Methods Appraisal Tool (MMAT)[19] combined with independent reviewer screening.

After review and removal of irrelevant and duplicates, 49 articles were included, 27 published pre-Covid (before Jan 2020) and 22 between Jan 2020 – Oct 2021 (Appendix 1). Of these, >90% originated in the US, the rest from Australia, Korea, and the UK. The majority were conducted in admissions to Medicine with two from Pharmacy and one Veterinary Medicine. Study methodologies included single site cross-sectional/cohort or quasi-experimental studies. Meta-analysis/meta-synthesis were not possible due to study heterogeneity. Each article was read by up to two of the research team looking for insights and recommendations to optimise accessibility and fairness in online interviews.

Key References

35. Ballejos, M. P., Oglesbee, S., Hettema, J., Sapien, R. An equivalence study of interview platform: Does videoconference technology impact medical school acceptance rates of different groups?. Advances in Health Sciences Education: Theory and Practice. 2018;23(3):601–610. https://doi.org/http://dx.doi.org/10.1007/s10459-018-9817-2 Page 16/21

36. Bird, SB, Hern, G, Blomkalns, A, Deiorio NM, Haywood Y, Hiller KM, et al. Innovation in Residency Selection: The AAMC Standardized Video Interview. Academic Medicine: Journal of the Association of American Medical Colleges. 2019;94(10):1489–1497. https://doi.org/https://dx.doi.org/10.1097/ACM.0000000000002705

For peer review only

37. Bowers, K., Comp, G., Kalnow, A., Casey, J., Fraser, W., Lloyd, C., Little, A. Are Standardized Video Interview Scores Predictive of Interview Performance? Western Journal of Emergency Medicine. 2018;S18

38. Breitkopf, DM, Green, IC, Hopkins, MR, Torbenson, VE, Camp, CL, Turner, NS. Use of Asynchronous Video Interviews for Selecting Obstetrics and Gynaecology Residents. Obstetrics and Gynecology. 2019;134(4):9S-15S. https://doi.org/https://dx.doi.org/10.1097/AOG.0000000000003432

39. Chandler, N. M., Litz, C. N., Chang, H. L., Danielson, P. D., N.M., C., C.N., L., & H.L., C. Efficacy of Videoconference Interviews in the Paediatric Surgery Match. Journal of Surgical Education. 2019;76(2):420–426. https://doi.org/http://dx.doi.org/10.1016/j.jsurg.2018.08.010

40. Chung AS, Shah KH, Bond M, Ardolic B, Husain A, Li I, Cygan L, Caputo W, Shoenberger J, van Dermark J, Bronner J, Weizberg M. How Well Does the Standardized Video Interview Score Correlate with Traditional Interview Performance?. The Western Journal of Emergency Medicine. 2019;20(5):726–730. https://doi.org/https://dx.doi.org/10.5811/westjem.2019.7.42731

41. Cleland J, Chu J, Lim S, Low J, Lowe-Beer N, Kwek T. COVID-19: designing and conducting an on-line mini-multiple interview (MMI) in a dynamic landscape. Medical Teacher. 2020. May: 1-5. https://doi.org/10.1080/0142159X.2020.1762851

42. Daram, S. R., Wu, R., Tang, S.-J. J., S.R., D., R., W., Daram, S. R., … Tang, S.-J. J. Interview from anywhere: Feasibility and utility of web-based videoconference interviews in the gastroenterology fellowship selection process. American Journal of Gastroenterology. 2014;109(2):155–159. https://doi.org/10.1038/ajg.2013.278

43. Davis M, Haas M, Gottlieb M *et al.* Zooming In Versus Flying Out: Virtual Residency Interviews in the Era of COVID-19. *AEM Educ Train* 2020; 4: 443-446.

44. Egan, D. J., Husain, A., Bond, M. C., Caputo, W., Cygan, L., Van Dermark, J., et al. Standardized video interviews do not correlate to United States medical licensing examination step 1 and step 2 scores. Page 18/21 The Western Journal of Emergency Medicine. 2019;20(1):87–91. https://doi.org/http://dx.doi.org/10.5811/westjem.2018.11.39730

45. Edje, L., Miller, C., Kiefer, J., & Oram, D. Using Skype as an Alternative for Residency Selection Interviews. Journal of Graduate Medical Education. 2013;5(3):503–505. https://doi.org/10.4300/jgme-d-12-00152.1

46. Gallahue, F. E., Hiller, K. M., Bird, S. B., Calderone Haas, M. R., Deiorio, N. M., Hern, H. G., et al. The AAMC Standardized Video Interview: Reactions and Use by Residency Programs During the 2018 Application Cycle. Academic Medicine: Journal of the Association of American Medical Colleges. 2019;94(10):1506–1512. https://doi.org/https://dx.doi.org/10.1097/ACM.0000000000002714

47. Grova MM, Donohue SJ, Meyers MO, Kim HJ, Ollila DW. Direct Comparison of In-Person Versus Virtual Interviews for Complex General Surgical Oncology Fellowship in the COVID-19 Era. Ann Surg Oncol. 2021;28(4):1908-1915. doi:10.1245/s10434-020-09398-2

48. Hakes, E., Schnapp, B., Ritter, D., Kraut, A., Fallon, S., Brown, K., Westergaard, M. Communication and Professionalism: Comparing Standardized Video Interview Scores to Faculty Gestalt. Society for Academic Emergency Medicine. 2018;S276.

49. Hall, MM, Lewis, JJ, Joseph, JW, Ketterer, AR, Rosen, CL, Dubosh, NM. Standardized Video Interview Scores Correlate Poorly with Faculty and Patient Ratings. The Western Journal of Emergency Medicine. 2019;21(1):145–148. https://doi.org/https://dx.doi.org/10.5811/westjem.2019.11.44054

50. Healy, W. L., Bedair, H., & W.L., H. Videoconference Interviews for an Adult Reconstruction Fellowship: Lessons Learned. The Journal of Bone and Joint Surgery. American Volume. 2017;99(21):e114. https://doi.org/https://dx.doi.org/10.2106/JBJS.17.00322

51. Hopson, L. R., Dorfsman, M. L., Branzetti, J., Gisondi, M. A., Hart, D., Jordan, J., et al. Comparison of the Standardized Video Interview and Interview Assessments of Professionalism and Interpersonal Communication Skills in Emergency Medicine. AEM Education and Training. 2019;3(3):259–268. https://doi.org/https://dx.doi.org/10.1002/aet2.10346

52. Humbert, A., Pettit, K., Mugele, J., Turner, J., Morgan, Z., & Palmer, M. Correlation of the Standard Video Interview Score With an Established Application Review Process. Society for Academic Emergency Medicine. 2018;S98.

53. Huppert L A, Hsiao E C, Cho K C *et al.* Virtual interviews at graduate medical education training programs determining evidence-based best practices. *Acad Med* 2020. doi: 10.1097/ACM.0000000000003868.

---

54. Husain, A., Li, I., Ardolic, B., Bond, M. C., Shoenberger, J., Shah, K. H., et al. The Standardized Video Interview: How Does It Affect the Likelihood to Invite for a Residency Interview?. AEM Education and Training. 2019;3(3):226–232. https://doi.org/https://dx.doi.org/10.1002/aet2.10331

Inzana, K, Vanderstichel, R, Newman, S. Virtual Multiple Mini-Interviews for Veterinary Admissions. https://doi.org/10.3138/jvme-2020-0107 J Of Veterinary Medical Education

56. Joshi, A., Bloom, D. A., Spencer, A., Gaetke-Udager, K., & Cohan, R. H. Video Interviewing: A Review and Recommendations for Implementation in the Era of COVID-19 and Beyond. Academic radiology. 2020;27(9):1316–1322. https://doi.org/10.1016/j.acra.2020.05.020

57. Kok K, Chen L, Idris F, Mumin N, Ghani H, Zulkipli. Conducting multiple mini-interviews in the midst of COVID-19 pandemic. Korean J Med Educ. 2020 Dec; 32(4): 281–289. Published online 2020 Oct 28. doi: 10.3946/kjme.2020.175.

58. Krauss, W., Egan, D., Bond, M., Husain, A., White, M., Taylor, T., et al. Correlation Between Emergency Medicine Residency Applicant’s Standardized Video Interview Scores and United States Medical Licensing Examination Results. Society for Academic Emergency Medicine. 2018;S83

For peer review only

Kyong-Jee Kim, Nam Young Lee & Bum Sun Kwon. Benefits and Feasibility of Using Videos to Assess Medical School Applicants' Empathetic Abilities in Multiple Mini Interviews. *Medical Science Educator* volume 31, pages175–181 (2021)C

60. Lewis, J., Hall, M., Joseph, J., Dubosh, N., Ullman, E. Standardized Video Interview Scores Do Not Correlate With Attending Evaluations. Society for Academic Emergency Medicine, 2018;S229.

61. M., S.R., E., L.M., B., M.M., A., F.M., D., J.C., E., … H., F. Initial Experience with a Virtual Platform for Advanced Gastrointestinal Minimally Invasive Surgery Fellowship Interviews. Journal of the American College of Surgeons. 2020;231(6):670–678. https://doi.org/http://dx.doi.org/10.1016/j.jamcollsurg.2020.08.768

62. McAteer, R., Sundaram, S., Harkisoon, S., & Miller, J. Videoconference Interviews: A Timely Primary Care Residency Selection Approach. Journal of Graduate Medical Education. 2020;12(6):737–744. https://doi.org/https://dx.doi.org/10.4300/JGME-D-20-00248.1

63. McHugh, M., Kulstad, C., Van Dermark, J., Bischof, J. Do Standardized or Traditional Interview Questions Correlate With the Standardized Video Interview? Society for Academic Emergency Medicine. 2019;S216

64. Molina, G., Mehtsun, W. T., Qadan, M., Hause, K. C., Raut, C. P., & Fairweather, M. Virtual Interviews for the Complex General Surgical Oncology Fellowship: The Dana-Farber/Partners Experience. Annals of Surgical Oncology. 2020;27(9):3103–3106. https://doi.org/https://dx.doi.org/10.1245/s10434-020- 08778-y

65. Nutter, A., La Rosa, M., Olson, G. Perception of Candidates and Faculty on Maternal Fetal Medicine Fellowship Videoconference Interviewing. Obstetrics and Gynecology. 2020;S75.

Nwora C, M.D.[a] Allred, D, M.D.[b] Verduzco-Gutierrez, M, M.D.[c] Mitigating Bias in Virtual Interviews for Applicants Who are Underrepresented in Medicine. Journal of the National Medical Association Volume 113, Issue 1, February 2021, Pages 74-76

67. Patel T, Bedi H, Deitte L, Lewis P *et al.* Brave New World: Challenges and Opportunities in the COVID-19 virtual interview season. *Acad Radiol* 2020; 27: 1456-1460.

68. Proost, K., Germeys, F., & Vanderstukken, A. Applicants' pre-test reactions towards video interviews: the role of expected chances to demonstrate potential and to use nonverbal cues. European Journal of Work and Organizational Psychology. 2021;30(2):265-273. DOI: 10.1080/1359432X.2020.1817975

69. Sabesan V, Kapur N, Zemanek K, Levitt D, Vu T, Erp A. Implementation and evaluation of virtual multiple mini-interviews as a selection tool for entry into paediatric postgraduate training: A Queensland experience. Medical Teacher Published online: 30 Aug 2021.

https://doi.org/10.1080/0142159X.2021.1967906

70. Schnapp, B. H., Ritter, D., Kraut, A. S., Fallon, S., Westergaard, M. C. Assessing residency applicants' communication and professionalism: Standardized video interview scores compared to faculty gestalt. Western Journal of Emergency Medicine. 2019;20(1):132–137. https://doi.org/http://dx.di.org/10.5811/westjem.2018.10.39709

71. Shah SK, Arora, S, Skipper B, Kalishman S, Timm TC, Smith AY. Randomized evaluation of a web based interview process for urology resident selection. Journal of Urology. 2012;187(4):1380–1384. https://doi.org/http://dx.doi.org/10.1016/j.juro.2011.11.108

72. Singh, N D, DeMesa, DO, MPH, Pritzlaff, S, MD, Jung, M MD, MBA, Green, C MA, LMFT. Implementation of Virtual Multiple Mini-Interviews for Fellowship Recruitment . *Pain Medicine*, Volume 22, Issue 8, August 2021, Pages 1717–1721, https://doi.org/10.1093/pm/pnab141

73. Sripad, A. Videoconference Interviews for Female Pelvic Medicine and Reconstructive Surgery Fellowship During a Pandemic: The Candidate Experience. Female Pelvic Medicine & Reconstructive Surgery. 2020;S181.

74. Staicu, M., Hamby, C., Wychowski, M., Reiss, B. Facetime faceoff: evaluation of video conferencing as a novel pre-interview screen for a PGY-1 pharmacy residency. 2015;E182.

75. Temple, M., Lagzdins, M. Streamlining the residency interview process using Web-based teleconferencing. American Journal of Health-System Pharmacy. 2014;71:697-701. Doi: 10.2145/ajhp130406

76. Tiller D, O'Mara D, Rothnie I, Dunn S, Lee L, Roberts C. Internet-based multiple mini-interviews for candidate selection for graduate entry programmes. Med Educ. 2013;47(8):801-810. doi:10.1111/medu.12224

77. Turpin C, Steele K, Matuk-Villazon O, Rowland, K, Dayton, Horn, K. Rapid Transition to a Virtual Multiple Mini-Interview Admissions Process: A New Medical School's Experience During the COVID-19 Pandemic. Academic Medicine: August 2021 - Volume 96 - Issue 8 - p 1152-1155. doi: 10.1097/ACM.0000000000004179.

78. Ungtrakul T, Lamlertthon W, Boonchoo B, ,Auewarakul C. Virtual Multiple Mini-Interview during the COVID-19 Pandemic. Medical Education Adaptation. **https://doi.org/10.1111/medu.14207**

79. Vadi, M. G., Malkin, M. R., Lenart, J., Stier, G. R., Gatling, J. W., & Applegate, R. L. Comparison of web-based and face-to-face interviews for application to an anaesthesiology training program: a pilot study. International Journal of Medical Education. 2016;7:102–108. https://doi.org/10.5116/ijme.56e5.491a

80. Vining CC, Eng OS, Hogg ME, et al. Virtual Surgical Fellowship Recruitment During COVID-19 and Its Implications for Resident/Fellow Recruitment in the Future. Ann Surg Oncol. 2020;27(Suppl 3):911- 915. doi:10.1245/s10434-020-08623-2

81. Willis, J., Surles, T., Silverberg, M., Kendall, S., LoCascio, H., Gernsheimer, J., Schechter, J., Regan, A., Smith, T. Are Standardized Video Interview Scores Predictive of Interview Performance? Western Journal of Emergency Medicine. 2018;S5-S6.

82. Winfield-Dial, A., Chhabra, N., Schindlbeck, M., & Bowman, S. Demographic Differences Between High and Low Scorers on the Standardized Video Interview. Western Journal of Emergency Medicine. 2018;S48. https://escholarship.org/uc/item/68c0x4pf

For peer review only

83. Winfield-Dial, A., Chhabra, N., Schindlbeck, M., & Bowman, S. Applicant Attitudes Towards the Standardized Video Interview - An Interim Analysis. Western Journal of Emergency Medicine. 2018;S4. https://escholarship.org/uc/item/5xp7f587 Page 17/21

84. Yolanda,S, Wisnu, W, Wahjudi, J and Findyartini A. Adaptation of internet-based multiple mini-interviews in a limited-resource medical school during the coronavirus disease 2019 pandemic. Korean J Med Educ. 2020 Dec; 32(4): 281–289. doi: 10.3946/kjme.2020.175

For peer review only

Appendix 2. Estimated hierarchical confirmatory factor analytic (CFA) model for the SAMMI responses, with a general factor (g) and seven specific factors (f1 to f7), the latter representing the seven questions in the interview. Standardised factor loading are shown.

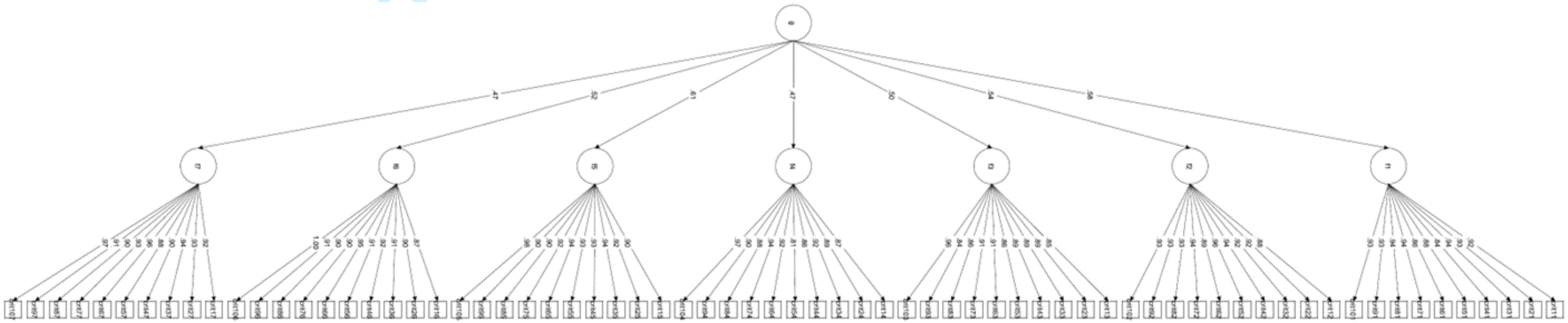

Appendix 3. Results of a parallel analysis using Pearson correlations on the response data to the SAMMI MMI.

Number of postulated factors	Mean of real data % of variance	Mean of random % of variance	95 percentile of random % of variance
1	29.2*	2.9	3.1
2	11.1*	2.8	3.0
3	9.5*	2.8	2.9
4	8.3*	2.7	2.8
5	7.6*	2.6	2.7
6	7.4*	2.6	2.7
7	6.2*	2.5	2.6
8	1.1	2.5	2.5
9	1.0	2.4	2.5

*Percentage of variance in the responses explained by the number of factors in the real data exceeded that for the random data.

For peer review only

Supplementary Table 1: Applicant and interviewer top positives and negatives

Applicant				
Positives				
Theme	Sub theme detail	N=158 (%)	% of total comments	Illustrative quote
Ease	Access, intuitive, convenient, flexible, simple	63 (40)	21	*"Quick, not time consuming, simple"*<br>*"Flexible, you can do it at your own time. It reduces stage fright. It is convenient, you can do it in your own home…"*
Reduced stress	More relaxed in own home, take my time, breaks available, start when wish	45 (28)	15	*"Its more relaxing to be in your own home instead of a new environment which for me is much less intimidating resulting in perhaps me performing better in the interview".*<br>*"… there is no external party criticising you in the moment, something which personally takes away a fair amount of nerves and stress from the process…"*
Fairer	Reduced costs (travel), reduced time away from other responsibilities	39 (25)	13	*"Its much easier in regard to travel for those who live far away, don't have the funds or time or who have other commitments".*

For peer review only

	(caring), the practice portal and availability of question text helped neurodiverse applicants			*"Reduces the possibility of bias during the interview process".* *"More inclusive".* *"Another great thing about this was I could do a practice question to get used to the layout of the interview, so I wasn't going into the interview completely blind".*
'Meet' staff	Able to see more of the university's community	9 (7)	3	*"I liked that there were 7 different people asking the questions…"*
Covid safe	Travel not required	2 (1)	1	*"Considering the situation with the pandemic, the online interview has helped to continue the process of admissions in a positive way…"*
Negatives				
Theme	Sub theme detail	N=140 (%)	% of total comments	Illustrative quote
Limited direct communication	Less personal, no conversation	48 (34)	16	*"… Impersonal, I feel like I cant make a connection or read the interviewers body language in the interview which I feel doesn't allow me to act how I usually do, and it felt unnatural".*

For peer review only

MMI	Pressure felt due to the timed methodology, presence of the countdown timer, lack of question face validity.	45 (32)	15	*"4 minutes per question was quite pressuring to fill"* *"Ticking down time was off-putting".* *"Could have asked more personal questions such as why this university and tell us about yourself, there was too many scenario questions which quite put us on the spot".*
Anticipated technical issues	Wi-Fi cut out	27 (19)	9	*"Technical issue (my screen froze) potential upload failure, potential loss of internet connection".*
Don't get vibe of staff	Not directly meeting staff	2 (1)	1	*"Lack of interaction between the student-tutor, the dialogue"*
Cannot ask questions		18 (13)	6	*"Not personalised, not able to ask questions"*
Interviewer				
Positives				
Theme	Sub theme detail	N=132 (%)	% of total comments	Illustrative quote
Convenient	Can prioritise workload. Flexible Quicker	82 (62%)	37	*"Efficient, less time consuming and able to prioritise your workload accordingly and complete interviews in chunks as opposed to one long stint"*

For peer review only

	Less stressful			*Don't have the issue of trying to find someone to cover like when another meeting comes up at the same time…"*
Fairer	Less unconscious bias<br>Reduced travel [costs]	18 (14%)	7	*"Reduced bias from different people asking the questions".*<br>*"Equity... no student gets help with prompts more than others"*
East to navigate	Simple	12 (9%)	5	*"Easy to use with clear instructions"*
Benefits applicants	Less stressed in own environment	12 (9%)	6	*"Separating the two events [interview/applicant day] will help manage anxiety and stress"*
Applicant assessment	Can get a [better] sense of the applicant	4 (3%)	2	*"I feel I can get a sense of the applicant through this process".*
Less worried about technical issues	Not one time/date dependent	3 (2%)	2	*"Much less stressful as I don't have to worry about the internet connection".*
Ability to rewatch	If cause for concern'	1 (1%)	1	*"Ability to rewatch for clarification".*
Negatives				
Theme	Sub theme detail	N=90 (%)	% of total comments	Illustrative quote
Less personal	Don't get feel for applicant	27 (30%)	12	*"I think it's better for applicants to speak to someone in person"*

				"I question whether it produces the same quality and depth of response".
Critique of MMI methodology	Timed circuit/countdown timer	22 (24%)	10	*"Encourages to me to make a snap decision"*<br>*"Cant ask follow-on questions…permits no probing".*
Communication assessment and build rapport difficult	Less able to assess non-verbal communication, social skills, spontaneous cognitive ability.	21 (23%)	9	*"Little in the way of holism".*<br>*"Disconnect perhaps for applicants".*<br>*"Does not allow markers to assess a candidates ability to respond and adapt to others".*
Limited support for nervous/stressed applicant	Can not help if upset or stressed	8 (9%)	4	*"No opportunity to provide any support if the candidate appears upset.. if they are struggling mentally or emotionally"..*
Tech related process concerns	Tech issues<br>Stress of Zoom/Teams online mode<br>Repetitive watching videos<br>Disadvantages those not familiar with tech	4 (5%)	2	*"Robotic process"*<br>*"Repetitive and boring watching multiple videos"*<br>*"Some people find Teams/Zoom very stressful and therefore we dont see the best of the candidate".*<br>*"Technical issues for some student may give them a disadvantage".*
Did not have any	Stated 'none'	5 (6%)	2	*"Don't have any"*

For peer review only

				"None to mention".
University marketing	Does not show university as warm and welcoming	3 (3%)	1	*"We are very nice and they do not see that"* *"Current students often state they chose Surrey due to the friendliness of the staff when first meeting them, my concern is if this is the first impression".*

For peer review only

STROBE Statement—checklist of items that should be included in reports of observational studies

	Item No	Recommendation	Page No
**Title and abstract**	1	(*a*) Indicate the study's design with a commonly used term in the title or the abstract	1
		(*b*) Provide in the abstract an informative and balanced summary of what was done and what was found	3-4
**Introduction**			
Background/rationale	2	Explain the scientific background and rationale for the investigation being reported	5-9
Objectives	3	State specific objectives, including any prespecified hypotheses	9
**Methods**			
Study design	4	Present key elements of study design early in the paper	9-10
Setting	5	Describe the setting, locations, and relevant dates, including periods of recruitment, exposure, follow-up, and data collection	10
Participants	6	(*a*) *Cohort study*—Give the eligibility criteria, and the sources and methods of selection of participants. Describe methods of follow-up<br>*Case-control study*—Give the eligibility criteria, and the sources and methods of case ascertainment and control selection. Give the rationale for the choice of cases and controls<br>*Cross-sectional study*—Give the eligibility criteria, and the sources and methods of selection of participants	11
		(*b*) *Cohort study*—For matched studies, give matching criteria and number of exposed and unexposed<br>*Case-control study*—For matched studies, give matching criteria and the number of controls per case	
Variables	7	Clearly define all outcomes, exposures, predictors, potential confounders, and effect modifiers. Give diagnostic criteria, if applicable	12
Data sources/ measurement	8*	For each variable of interest, give sources of data and details of methods of assessment (measurement). Describe comparability of assessment methods if there is more than one group	*12*
Bias	9	Describe any efforts to address potential sources of bias	30
Study size	10	Explain how the study size was arrived at	11
Quantitative variables	11	Explain how quantitative variables were handled in the analyses. If applicable, describe which groupings were chosen and why	12
Statistical methods	12	(*a*) Describe all statistical methods, including those used to control for confounding	12
		(*b*) Describe any methods used to examine subgroups and interactions	12
		(*c*) Explain how missing data were addressed	12
		(*d*) *Cohort study*—If applicable, explain how loss to follow-up was addressed<br>*Case-control study*—If applicable, explain how matching of cases and controls was addressed<br>*Cross-sectional study*—If applicable, describe analytical methods taking account of sampling strategy	12
		(*e*) Describe any sensitivity analyses	

For peer review only - http://bmjopen.bmj.com/site/about/guidelines.xhtml

**Results**			
Participants	13*	(a) Report numbers of individuals at each stage of study—eg numbers potentially eligible, examined for eligibility, confirmed eligible, included in the study, completing follow-up, and analysed	12<br>16-17
		(b) Give reasons for non-participation at each stage	
		(c) Consider use of a flow diagram	
Descriptive data	14*	(a) Give characteristics of study participants (eg demographic, clinical, social) and information on exposures and potential confounders	13
		(b) Indicate number of participants with missing data for each variable of interest	
		(c) *Cohort study*—Summarise follow-up time (eg, average and total amount)	
Outcome data	15*	*Cohort study*—Report numbers of outcome events or summary measures over time	
		Case-control study—Report numbers in each exposure category, or summary measures of exposure	
		Cross-sectional study—Report numbers of outcome events or summary measures	*13-14*
Main results	16	(*a*) Give unadjusted estimates and, if applicable, confounder-adjusted estimates and their precision (eg, 95% confidence interval). Make clear which confounders were adjusted for and why they were included	14-27
		(*b*) Report category boundaries when continuous variables were categorized	
		(*c*) If relevant, consider translating estimates of relative risk into absolute risk for a meaningful time period	
Other analyses	17	Report other analyses done—eg analyses of subgroups and interactions, and sensitivity analyses	15-16
**Discussion**			
Key results	18	Summarise key results with reference to study objectives	27
Limitations	19	Discuss limitations of the study, taking into account sources of potential bias or imprecision. Discuss both direction and magnitude of any potential bias	30
Interpretation	20	Give a cautious overall interpretation of results considering objectives, limitations, multiplicity of analyses, results from similar studies, and other relevant evidence	28
Generalisability	21	Discuss the generalisability (external validity) of the study results	30
**Other information**			
Funding	22	Give the source of funding and the role of the funders for the present study and, if applicable, for the original study on which the present article is based	2

*Give information separately for cases and controls in case-control studies and, if applicable, for exposed and unexposed groups in cohort and cross-sectional studies.

For peer review only - http://bmjopen.bmj.com/site/about/guidelines.xhtml

For peer review only

BMJ Open

Cross-sectional evaluation of an asynchronous Multiple Mini Interview (MMI) in selection to health professions training programmes with ten principles for fairness built-in

Journal:	*BMJ Open*
Manuscript ID	bmjopen-2023-074440.R2
Article Type:	Original research
Date Submitted by the Author:	30-Aug-2023
Complete List of Authors:	Callwood, Alison; University of Surrey Faculty of Health and Medical Sciences, FHMS<br>Harris, Jenny; University of Surrey Faculty of Health and Medical Sciences<br>Gillam, Lee; University of Surrey,<br>Roberts, Sarah; University of Surrey Faculty of Health and Medical Sciences<br>Kubacki, Angela; St George's University of London, IMBE<br>Christidis, Angelos; University of Surrey Faculty of Engineering and Physical Sciences<br>Tiffin, Paul; University of York, Department of Health Sciences
**Primary Subject Heading**:	Medical education and training
Secondary Subject Heading:	Nursing
Keywords:	MEDICAL EDUCATION & TRAINING, Nursing Care, Health Education

SCHOLARONE™
Manuscripts

I, the Submitting Author has the right to grant and does grant on behalf of all authors of the Work (as defined in the below author licence), an exclusive licence and/or a non-exclusive licence for contributions from authors who are: i) UK Crown employees; ii) where BMJ has agreed a CC-BY licence shall apply, and/or iii) in accordance with the terms applicable for US Federal Government officers or employees acting as part of their official duties; on a worldwide, perpetual, irrevocable, royalty-free basis to BMJ Publishing Group Ltd ("BMJ") its licensees and where the relevant Journal is co-owned by BMJ to the co-owners of the Journal, to publish the Work in this journal and any other BMJ products and to exploit all rights, as set out in our licence.

The Submitting Author accepts and understands that any supply made under these terms is made by BMJ to the Submitting Author unless you are acting as an employee on behalf of your employer or a postgraduate student of an affiliated institution which is paying any applicable article publishing charge ("APC") for Open Access articles. Where the Submitting Author wishes to make the Work available on an Open Access basis (and intends to pay the relevant APC), the terms of reuse of such Open Access shall be governed by a Creative Commons licence – details of these licences and which Creative Commons licence will apply to this Work are set out in our licence referred to above.

Other than as permitted in any relevant BMJ Author's Self Archiving Policies, I confirm this Work has not been accepted for publication elsewhere, is not being considered for publication elsewhere and does not duplicate material already published. I confirm all authors consent to publication of this Work and authorise the granting of this licence.

For peer review only

Cross-sectional evaluation of an asynchronous Multiple Mini Interview (MMI) in selection to health professions training programmes with ten principles for fairness built-in

Running head: evaluation of an asynchronous MMI

Corresponding author: Dr Alison Callwood Faculty of Health and Medical Sciences[1], University of Surrey, 30 Priestley Road, Guildford, Surrey, GU2 7YH, UK a.callwood@surrey.ac.uk. +44 7796956143

Dr Jenny Harris[1] jen.harris@surrey.ac.uk

Dr Lee Gillam[2] Faculty of Engineering and Physical Sciences, University of Surrey, Guildford, GU2 7XH, UK l.gillam@surrey.ac.uk

Mrs Sarah Roberts[1] sarah.roberts@surrey.ac.uk

Angela Kubacki[3] St George's University of London, London, UK akubacki@sgul.ac.uk

Angelos Christidis[2] Angelos.Christidis@surrey.ac.uk

Paul A Tiffin[4] University of York, York, UK paul.tiffin@york.ac.uk

Key Words

Multiple Mini Interview, equity, diversity and inclusion, fairness, asynchronous, online interviews

Word count 4371

For peer review only

Abstract

Objectives

We aimed to explore psychometric properties of the first known online asynchronous Multiple Mini-Interview (MMI) designed for fairness with sub-group analysis by key characteristics, usability, and acceptability.

Design: Cross-discipline multi-method evaluation

Setting: One United Kingdom University

Participants: Applicants to Nursing, Midwifery and Paramedic Science under-graduate programmes during 2021-2022.

Primary, secondary outcome measures

Psychometric properties (internal consistency, construct validity, dimensionality) were assessed using Cronbach's alpha (α), parallel analysis (PA), Schmid-Leiman transformation and ordinal confirmatory factor analysis (CFA). Usability and acceptability were evaluated using descriptive statistics and conventional content analysis.

Methods

The system was configured in a seven question four-minute MMI. Applicants' video-recorded their answers which were later assessed by interviewers and scores summed. Applicants and interviewers completed online evaluation questionnaires.

Results

Performance data from 712 applicants determined good-excellent reliability for the asynchronous MMI (mean α 0.72) with similar results across sub-groups (gender, age, disability/support needs, UK/non-UK). Parallel analysis and factor analysis results suggested there were seven factors relating to the MMI questions with an underlying general factor that explained the variance in observed candidate responses. A confirmatory factor analysis testing a seven-factor hierarchical model showed an excellent fit to the data (Confirmatory Fit Index =0.99), Tucker Lewis Index =0.99, RMSE=0.034).

For peer review only - http://bmjopen.bmj.com/site/about/guidelines.xhtml

For peer review only

Applicants (n=210) viewed the flexibility, relaxed environment, and cost savings advantageous. Interviewers (n=65) reported the system to be intuitive, flexible with >70% time saved compared to face-to-face interviews. Reduced personal communication was cited as the principle disadvantage.

Conclusions

We found that the asynchronous MMI was reliable, time-efficient, fair, and acceptable. These novel, low-cost insights are applicable across health professions selection internationally informing the future configuration of online interviews to ensure workforces represent the societies they serve.

Study strengths and limitations of this study

- The underpinning theoretical approach aligned with an iterative process necessary to design a new technology to reduce bias.
- The large sample enabled us to assess psychometric properties with sub-group analysis for the first time in this context.
- The study provides perspectives from one large site; a necessary step to inform a planned international multi-site evaluation.
- The multi-method design provided insights necessary to embed fairness into online selection approaches in the absence of best practice guidance.

For peer review only

For peer review only - http://bmjopen.bmj.com/site/about/guidelines.xhtml

Introduction

Ensuring equity, inclusivity, and diversity in selection to health professions training programmes is recognised internationally as an ethical and practical imperative [1,2]. Globalisation and increased workforce pressures amplify this need [3]. Fulfilling our responsibility to ensure fair selection is complex due to unintended biases that are intrinsic to human assessment compounded by recent unprecedented change to online interviews in the absence of published evidence [4,5,6,7]. Historically, health professions' selection has been mainly face-to-face using unstructured or structured approaches including panel interviews, group interviews, assessment centres and multiple mini-interviews (MMIs) [8]. MMIs are a series of short, focused interactions with a number of different interviewers. The multi-question format featuring structured scoring proforma with interviewers who have no prior knowledge of applicants, is designed to mitigate the potential impact of interviewer bias [9]. MMIs have been shown to be a feasible, acceptable, valid, and reliable candidate selection approach across health professions. None-the-less, as a face-to-face method, MMIs can be costly, resource intensive and influenced by unintended bias [10].

Online interviews were a relatively uncommon occurrence in selection to health professions before the pandemic. Approaches included Skype-based MMIs, asynchronous MMIs and asynchronous panel interviews [11]. Research outside the field of healthcare has shown asynchronous video interviews to be faster, cheaper, and require less employee time, easing scheduling burden and allowing for more applicants to be screened [12]. This can potentially increase the number of applicants who would have otherwise not had the opportunity to be interviewed.

During the pandemic, it was vital to ensure the continuance of recruitment to health professions. This resulted in rapid adaption to using online interviews facilitated by videoconference technology [13,14]. Recent research suggests online interviews like MMIs are feasible and acceptable provided reliable high-speed internet connection is available. However, access to reliable Wi-Fi is not always

For peer review only - http://bmjopen.bmj.com/site/about/guidelines.xhtml

possible [11]. In an asynchronous approach, applicants record their interviews at a convenient time and place, alleviating potential technical issues.

In live synchronous interviews, nuanced inconsistencies in, for example, tone and intonation, can arise in the way interviewers ask questions. Consistency of questioning across applicants is assured in asynchronous interviews through the use of pre-recorded interview questions. Fairness is further ensured with the avoidance of nonadherence to set questions which can occur in live interviews when applicants and interviewers serendipitously find something in common and deviate to discuss this [12,15].

'Fairness' in this article is conceptualised as the quality of treating people equally or in a way that is right or reasonable. That encapsulates perceived fairness by participants as well as that borne out in data. Consensus on the design of online interviews to optimise applicant accessibility and usability and mitigate potential unfairness issues for people across demographics, abilities, and disabilities is not readily available [15]. To address this, we applied to Innovate UK, the United Kingdom's innovation agency (2020-2021) to build and evaluate what we believe was, the first (proof-of-concept, PoC) asynchronous videoconference facilitated interview and assessment system uniquely grounded in the MMI method (Figure 1).

Asynchronous MMI Platform Development Summary

The asynchronous MMI is an on-demand videoconference interview where applicants log onto an online portal to complete their interview. The interview is a remote experience, where the system records applicants' short video responses to MMI questions in a timed process emulating the face-to-face MMI method. There is no synchronous, bidirectional communication, and no interviewer to

For peer review only - http://bmjopen.bmj.com/site/about/guidelines.xhtml

interact with; instead, questions are pre-recorded by interviewers. Applicants' recorded responses are scored by an interview assessor at a convenient later date.

To inform the design, we undertook a rapid review of literature published between 2011-2021, guided by a five-stage process [16, 17] to help us understand how video-based interviews compare with face-to-face in terms of implementation and fairness including what makes for an optimising experience (details provided in Appendix 1).

Indicative themes were elicited and assimilated into the following ten key principles and the system was re-configured accordingly:

- Recognise potential issues with stereotype threats and belonging uncertainty that may impact on candidates' performance and use language that supports the affirmation of values e.g., "well done for getting this far".
- Incorporate encouraging words/phrases into the interview dialogue, as well as any communications circulated to applicants (e.g., "good luck").
- Soften the language of technical instructions e.g., "when you are ready …" or "when you have familiarised yourself with…".
- Reduce the verbal load of interview content particularly for neurodivergent applicants.
- Accommodate access and engagement for neurodivergent applicants with extra time, adjusted fonts and a tailored user-interface (UI) including background colours.
- Provide opportunities for candidates to familiarise themselves with the UI and format prior to their interview though a practice portal.
- Recommend generic, blank backgrounds for video or videoconference facilitated interviews or, if not possible, advise blurred backdrops.
- Ensure diversity of interviewers for pre-recorded videos and those assessing them including gender, age and ethnicity, experts-by-experience, and other stakeholders.

For peer review only - http://bmjopen.bmj.com/site/about/guidelines.xhtml

- Avoid culturally sensitive subject areas, language, age and ability bias in interview content.
- Ensure the use of inclusive, gender-neutral language with appropriate pronouns e.g., ‘they/them/their’.

The focus of the article is to detail the study undertaken to evaluate the psychometric properties of the asynchronous MMI. We aimed to explore reliability (internal consistency) for all users with sub-group analysis by key characteristics (age, gender, nationality, disability/additional support needs), construct validity, dimensionality, acceptability, and usability.

In this context disability refers to a person who self-identifies with physical or mental impairment, and the impairment has a substantial and long-term adverse effect on the person's ability to carry out normal day-to-day activities. Not all neurodivergent people consider themselves ‘disabled’ but instead neurological conditions are viewed a result of normal variations in the human genome but where additional support might be required. We therefore use the term ‘support need’ to include neurodiverse applicants.

Methods

The MMI questions were developed and tested by the university’s community emulating our previously established in-person processes. A diverse range of individuals including academic staff, service users and practice partners were individually videorecorded asking MMI questions. The video recordings were uploaded and the system, configured in a seven question, four-minute MMI with one minute between questions.

The asynchronous online MMI system was adopted for selection to Health Professions undergraduate programmes (Adult, Child, Mental Health Nursing, Midwifery, and Paramedic Science) at one UK university during 2021/22 recruitment cycle. All applicants to these programmes were invited to register for a one-week slot that was convenient for them. At the start of their selected slot, applicants

For peer review only - http://bmjopen.bmj.com/site/about/guidelines.xhtml

were emailed a link to the system. Here they could access the practice portal to check their Wi-Fi speed, familiarise themselves with the user interface/key functionality, become conversant with the process through a detailed instructional video, and practice a question. When they felt prepared, they recorded their interview answers. To mirror our previous in-person process, once they had begun, applicants could not stop their MMI and start again. Any technical issues including those without the necessary equipment and/or Wi-Fi connectivity were supported to enable their successful completion on an individual basis.

All individuals at the university who would usually interview applicants as part of their role took part in this new process. These included academic staff, service users and practice partners. They were invited to self-allocate to one-week interview assessment slots. Thereafter they received a link and code to access the system to review and assess applicant interviews against uploaded scoring rubrics live on the system. Each interviewer was allocated a number of applicant's video responses to a single question thereby aligning with the principles of an MMI. Also, emulating MMI methodology, a 'red flag' option could be ticked, and details populated in a text box if a cause for concern was raised in applicants' answers. Applicant's performance scores were downloaded at the end of the process to inform offer/reject decisions by university Admissions Officers.

University Admissions Officers conducted an internal resource evaluation to explore time and cost spent on the asynchronous MMI compared with face-to-face and Zoom-facilitated MMIs.

Design

This study was underpinned by Olsen and Eoyang's theory of 'Complex Adaptive Systems' [18]. This is characterised by an adaptive and iterative working style appropriate when developing and optimising the system for fairness and reliability in the absence of any known precedent. We also grounded the system design in Gilliland's [19] justice-based model. According to their theory, a selection system's

For peer review only - http://bmjopen.bmj.com/site/about/guidelines.xhtml

adherence to procedural and distributive justice rules promotes applicants' perceptions of fairness. Procedural justice rules relate to the approach used to derive decisions, in this case an asynchronous MMIs. It includes the formal process (opportunity to perform and administration), explanation (feedback, information, and transparency), and interpersonal communication throughout the selection process. Distributive justice rules encompass adherence to equity when determining selection outcomes [19].

Participant recruitment

All applicants (n=712 at the data collection point) to the UK university and all interviewers (n=96) who assessed applicants were invited to evaluate the system.

Patient and Public Involvement

The university's service user group were supportive of the move to online asynchronous interviews. One service user acted as an interviewer by videorecording an MMI question. A further three were interview assessors, thereby continuing an established model at this university of service user involvement in recruitment. The service user group were also involved in the MMI question writing by reviewing draft questions and providing feedback. In the seven-question circuit, one practice partner video recorded an MMI question and eight assessed applicants' videos.

Data Collection

Applicant interview performance data, routinely collected to inform offer/reject decisions, were used in the reliability analyses. Applicant interviews were scored against 10 question-specific criteria on a seven-point Likert-type scale with descriptive anchors for the seven questions. Criteria details are withheld for test security. Applicant interview scores were summed for each applicant.

To assess usability, applicants were invited to complete an online semi-structured evaluation questionnaire hosted on Qualtrics[22] once they had received their interview outcome decision.

For peer review only - http://bmjopen.bmj.com/site/about/guidelines.xhtml

To assess acceptability, interviewers were invited to evaluate the process at the end of the recruitment cycle through an online semi-structured questionnaire also hosted on Qualtrics [20].

Analysis

We used applicant scores across questions to explore reliability (internal consistency) for all users and a randomly selected sub-sample self-reporting key characteristic including gender, age (<20 or 20+), nationality (UK/non-UK) and disability/support needs (absence/presence) using Cronbach's alpha. (Stata, version 16.1, StataCorp LLC) [21]. Cronbach's alpha, as a conventional measure of internal consistency (reliability) assumes that item responses are unidimensional, meaning that they are reflective of only one underlying construct or factor. However, this may not always be the case therefore scale dimensionality and was assessed using a parallel analysis and confirmatory factor analysis (CFA)[22,23]. The parallel analysis and reliability values were derived using the software package FACTOR [24]. The ordinal factor analyses were conducted in Mplus [25] version 8.8. Usability and acceptability were explored with descriptive statistics (closed questions) and conventional content analysis [26] (open questions).

Results

Sample Characteristics

Data were available from 712 applicants to Nursing, Midwifery and Paramedic Science programmes at the data collection point on 1st May 2022 and for key characteristics for the sub-sample (n= 284) (Table 1). Disabilities self-reported by interviewers were reduced hearing and visual acuity. Applicants reported neurodivergent challenges across the spectrum of dyslexia, dyspraxia, and attention deficit hyperactivity disorder (ADHD).

Table 1: Participant self-identified characteristics

For peer review only

Interview Assessors n= 65				Applicant Sub-sample n=284		
		N	%		N	%
Age (years)	25 and under	0	0	<20	193	68
	26-35	8	13			
	36-45	18	28	>20	91	32
	45+	35	53			
	Prefer not to say	4	6			
Gender	Female	55	84	Female	230	81
	Male	7	10	Male	54	19
	Other	0	0	Other	0	0
	Prefer not to say	3	6	Prefer not to say	0	0
Disability/additional support needs	No	60	93	No	228	80
	Yes	5	7	Yes (64% neurodiverse, 34% mental health, 2% hearing challenges)	58	20
	Prefer not to say	0	0	Prefer not to say	0	0
Ethnicity	White (English/Welsh/Scottish/Northern Irish, Irish, Gypsy, Irish Traveller, Other White background)	61	94	UK/Ireland	229	81
	Mixed/multiple Ethnic Groups (White and Black Caribbean, White and Black African, Any other Mixed Background)	2	3	Non-UK/Ireland	55	19
	Asian/Asian British (Indian, Pakistani, Bangladeshi, Chinese, Any other Asian background)	0	0	N/A	N/A	
	Other (Arab, any other ethnic group)	0	0			

For peer review only

	Prefer not to say	2	3			
Role	University Health Sciences Staff	46	71		N/A	N/A
	Practice Partner	10	16			
	Service user	9	13			
	Prefer not to say	0	0			

Reliability and assessment of fairness

Reliability

Applicant data were shown to be normally distributed (Kurtosis 0.000, p 0.005) and symmetrical with skewness 1.0.

Internal consistency was good-excellent across scenario questions (for n=712 applicants) (mean Cronbach's α 0.72 (range 0.64-0.89). Sub-group analyses showed similarly positive results with mean α Female/Male: 0.74/0.87 (range 0.70-0.89); age: <20years/ >21 years 0.76/0.83 (range 0.72-0.86), disability/additional challenges/non-disability: 0.88/0.78 (range 0.74-0.89) and UK/non-UK 0.78/0.0.77 (range 0.72-0.83) (Table 2).

Dimensionality/construct validity

The results of the parallel analysis suggested a maximum of seven dimensions underlay the response pattern. A Schmid-Leiman [27] transformation can be used to understand if a factor analytic model is best understood as a hierarchical in nature. This partitions observed variance into that explained by one or more general ('G') factors underlying three or more specific factors (a hierarchical model will be mathematically 'just identified' by three factors) [27]. The results of the Schmid-Leiman transformed factor analysis indicated a seven-factor solution, relating to the MMI scenarios, with an underlying general factor which substantially loaded on all the former seven factors. An ordinal Confirmatory Factor Analysis (CFA) to test this seven-factor hierarchical model showed an excellent fit to the data (Confirmatory Fit Index (CFI)=0.99, Tucker Lewis Index (TLI)=0.99, RMSE=0.034). In

For peer review only

contrast, a one factor model showed a poor fit to the response data (CFI=0.70, TLI=0.69, RMSEA=0.19) (Appendix 2)

The results of the parallel analysis suggested a maximum number of seven plausible factors. The variance explained by additional postulated factors did not exceed that observed for the random data generated. (Appendix 3).

Table 2 MMI question reliability (internal consistency) N=712

Question (total mean)	Obs	Mean	St Dev	Min	Max	Item-test correlation	Item-rest correlation	Cronbach's alpha
1	765	48.6732	10.3966	10	70	0.6541	0.4677	0.68
2	795	51.57107	9.70229	10	70	0.6434	0.4532	0.67
3	790	51.08101	9.10597	10	70	0.599	0.421	0.69
4	809	50.23486	8.97655	10	70	0.5826	0.4001	0.70
5	762	50.27428	9.88047	10	70	0.6624	0.4941	0.67
6	765	51.79869	8.7486	20	70	0.6132	0.4484	0.69
7	712	50.10499	11.0865	14	70	0.6076	0.3871	0.70
Scale reliability coefficient								0.7216
Sub-group reliability n=284								
Question	Cronbach's alpha							

For peer review only - http://bmjopen.bmj.com/site/about/guidelines.xhtml

(total score)	Age		Self-identified gender		Self-declared disability		Country of birth	
	< 20 years	> 20 years	Female	Male	Yes	Non	UK/Ireland	Non-UK/Ireland
1	0.74	0.83	0.73	0.87	0.88	0.76	0.78	0.74
2	0.74	0.83	0.72	0.87	0.88	0.76	0.77	0.75
3	0.75	0.83	0.74	0.87	0.87	0.77	0.78	0.77
4	0.77	0.85	0.76	0.89	0.88	0.79	0.79	0.79
5	0.73	0.82	0.71	0.86	0.87	0.75	0.76	0.73
6	0.77	0.84	0.76	0.87	0.87	0.79	0.79	0.79
7	0.79	0.87	0.78	0.88	0.89	0.80	0.80	0.86
Mean	0.76	0.83	0.74	0.87	0.88	0.78	0.78	0.77

Usability (applicants)

The online evaluation was completed by 210 applicants (29% response rate). The majority were under 20 years of age, self-identified as white female and with representation from across programmes (Table 3).

Table 3 Applicant Characteristics and usability evaluation

N=210

For peer review only

Demographics		N	%
Age	<20	120	57
	20+	90	43
Self-identified gender	Female	172	82
	Male	36	17
	Other/non-binary	2	1
Nationality	UK/Ireland	134	64
	Non-UK/ Ireland	68	32
	Prefer not to say	8	4
Programme representation			
Nursing	Adult	42	20
	Child	34	16
	Mental Health	52	25
Midwifery		32	15
Paramedic Science		50	24
Closed question responses summary data			
Had not taken an asynchronous MMI like this before			94%
Found the instructions helpful/very helpful			90%
Did not experience technical issues			79%
Said the one minute between questions was ‘about right’			75%
Found the probe questions helpful/very helpful			66%
Four minutes was about the right amount of time			65%
Open question free text responses			
Question		Response %	

For peer review only

How do you feel about videorecording and uploading your MMI responses as part of a 'new look' interview process triggered by Covid-19 social distancing restrictions?	Happy/very happy	67	Not very happy/not at all happy	33
Please tell us about your views on online interviews generally	Accepting/very accepting	66	Partly accepting/ Not at all accepting	35
Do you see a future for online interviews like the one you have used with us?	Yes/definitely yes	68	Probably not/Definitely not	32

The majority of applicants had not undertaken an asynchronous MMI previously. Overall applicants found the instructions either helpful or very helpful and did not experience any technical issues. From a setup perspective, four minutes was considered the right amount of time by over half with one-third stating it was too short. We asked additional overarching open free text questions regarding applicants' views on online interviews (Table 3) as well as their overall 'top' positives and 'top' negatives of system (Table 4 summary, Supplementary Table 1).

We received 158 separate positive comments and 140 negative comments in relation to the questions asking for 'top' positives and 'top' negatives, Notably, 93% of the positive comments centred around three themes: Ease (40%), reduced stress (28%) and fair (25%). We received fewer negative comments overall and these were split into two main themes: limited direct communication (34%) and critique of the MMI process itself (32%). Nineteen percent of respondents raised technical issues as a potential negative issue. However, it appears that as around 80% did not experience these in reality. The twelve

For peer review only - http://bmjopen.bmj.com/site/about/guidelines.xhtml

respondents that did experience technical issues cited: buffering (3), frozen screen (2), crash (2), video skipping (2), screen scaling (1), microphone (1) and upload (1). They all subsequently successfully completed their interview at a second attempt.

For peer review only

For peer review only - http://bmjopen.bmj.com/site/about/guidelines.xhtml

Table 4 Summary Applicant and interviewer top positives and negatives

Applicant				
Positives				
Theme	Sub theme detail	N=158 (%)	% of total comments	Illustrative quote
Ease	Access, intuitive, convenient, flexible, simple	63 (40)	21	*"Quick, not time consuming, simple"*
Reduced stress	More relaxed in own home, take my time, breaks available, start when wish	45 (28)	15	*"It's more relaxing to be in your own home instead of a new environment which for me is much less intimidating resulting in perhaps me performing better in the interview".*
Fairer	Reduced costs (travel), reduced time away from other responsibilities (caring), the practice portal and availability of question text helped neurodiverse applicants	39 (25)	13	*"It's much easier in regard to travel for those who live far away, don't have the funds or time or who have other commitments".* *"Reduces the possibility of bias during the interview process".* *"…I could do a practice question to get used to the layout of the interview, so I wasn't going into the interview completely blind".*
'Meet' staff	Able to see more of the university's community	9 (7)	3	*"I liked that there were 7 different people asking the questions…"*
Covid safe	Travel not required	2 (1)	1	*"…Has helped to continue the process of admissions in a positive way…"*
Negatives				
Limited direct communication	Less personal, no conversation	48 (34)	16	*"… Impersonal, I feel like I can't make a connection or read the interviewers body language in the interview…"*
MMI	Pressure felt due to the timed methodology, presence of the countdown timer	45 (32)	15	*"4 minutes per question was quite pressuring to fill".* *"Ticking down time was off-putting".*
Technical issues	Wi-Fi cut out	27 (19)	9	"*My screen froze, potential upload failure, loss of connection".*
Don't get staff vibe	Not directly meeting staff	2 (1)	1	*"Lack of interaction between the student-tutor, the dialogue"*
Cannot ask questions		18 (13)	6	*"Not personalised, not able to ask questions"*

For peer review only - http://bmjopen.bmj.com/site/about/guidelines.xhtml

Interviewer				
Positives				
Convenient	Can prioritise workload. Flexible. Quicker<br>Less stressful	82 (62%)	37	*"Efficient, less time consuming and able to prioritise your workload and complete interviews in chunks as opposed to one long stint"*
Fairer	Less unconscious bias<br>Reduced travel [costs]	18 (14%)	7	*"Reduced bias from different people asking the questions".*<br>*"Equity... no student gets help with prompts more than others"*
East to navigate	Simple	12 (9%)	5	*"Easy to use with clear instructions"*
Benefits applicants	Less stressed in own environment	12 (9%)	6	*"Separating the two events [interview/applicant day] will help manage anxiety and stress"*
Applicant assessment	Can get a [better] sense of the applicant	4 (3%)	2	*"I feel I can get a sense of the applicant through this process".*
Less worried about technical issues	Not one time/date dependent	3 (2%)	2	*"Much less stressful as I don't have to worry about the internet connection".*
Ability to rewatch	If cause for concern'	1 (1%)	1	*"Ability to rewatch for clarification".*
Negatives				
Less personal	Don't get feel for applicant	27 (30%)	12	*"I think it's better for applicants to speak to someone in person".*<br>*"I question whether it produces the same quality of response".*
Critique of MMI methodology	Timed circuit/countdown timer	22 (24%)	10	*"Encourages to me to make a snap decision".*<br>*"Can't ask follow-on questions...permits no probing".*
Communication/building rapport difficult	Less able to assess non-verbal communication, social skills, spontaneous cognitive ability.	21 (23%)	9	*"Little in the way of holism".*<br>*"Disconnect perhaps for applicants".*
Limited support for nervous applicants	Cannot help if upset or stressed	8 (9%)	4	*"No opportunity to provide any support if the candidate appears upset.. if they are struggling mentally or emotionally"..*
Tech related process concerns	Tech issues<br>Stress of Zoom/Teams online mode	4 (5%)	2	*"Robotic process"*<br>*"Repetitive and boring watching multiple videos"*<br>*"Technical issues for some student may give them a disadvantage".*
Did not have any	Stated 'none'	5 (6%)	2	*"Don't have any"*

For peer review only - http://bmjopen.bmj.com/site/about/guidelines.xhtml

Acceptability (Interviewers)

Sixty-five interviewers took part in the online evaluation representing a 71% response rate. The majority were white British female university staff, over the age of 45 years with no declared disability (Table 1). This is representative of the University Faculty staff profile which is located in the Southeast of the UK.

Ninety-six percent (n=62) of interviewers found the system intuitive, easy to use and reported a perceived reduction in stress. They primarily attributed this to increased convenience and flexibility. A 70%-time reduction was independently reported by our Admissions Officers. They estimated this based on the time spent on other interview approaches (face-to-face MMIs, Zoom-facilitated MMIs compared with our asynchronous MMIs; categorised into pre-interview communications, set up, staff recruitment (including covering sick time), interview facilitation and post-interview communications (Appendix 4). The majority of the time saving was ascribed directly to the asynchronous modality which removed the need for staff to either facilitate face-to-face or online live interviews. Additionally, the asynchronous approach alleviated the pressures of last-minute non-availability of interviewers particularly practice partners as they were not tied to one scheduled day/time but had a time period (1 week) within which they could assess the interview recordings.

None of the interviewer assessors stated that they had used an asynchronous online MMI previously. Almost all (96%) found it easy to use and the user interface intuitive (92%). Less than 10% reported technical issues other than download issues which were resolved. Five percent were 'not accepting' of using the asynchronous MMI in the future.

We were interested to better understand whether interviewers felt communication could be assessed in an asynchronous modality. Thirty-three percent said 'yes', 54% 'somewhat' with 13% of respondents (n=6) responded 'no'. To generate more in depth insights we asked interviewers their top positives and negatives of the system. These are presented in Table 4. We received 132 positive and 90 negative comments. The majority of positive comments (94%) related to perceived

For peer review only - http://bmjopen.bmj.com/site/about/guidelines.xhtml

convenience (62%), fairness (14%) ease of navigation (9%) and benefits for the applicant (9%). Negative comments were split more evenly into perceptions of their being less personal (30%), critique of MMI methodology (24%), limited communication assessment and ability to build rapport (23%), and 5% had technology-related process concerns. Six percent cited 'none'.

Discussion

These findings suggest the online asynchronous MMI is reliable, fair, time-efficient, and acceptable. The results of the factor analyses infer that there are scenario-level effects but that that these all relate to an underlying general factor indicative that the process is assessing different dimensions/constructs relevant to health care. These could be method effects or alternatively conceptualised as representing different aspects of the interpersonal procedural knowledge required to perform well on the MMI.

This platform is the only known custom built asynchronous online interview emulating the MMI methodology. Cognisant of Gilliland's [19] procedural and distributive justice rules, our aim was to optimise applicant accessibility through building principles for fairness into the MMI design and system set up. The reliability results and usability and acceptability evaluation signal this was largely achieved which is critical to applicants perception of fairness [28]. These data suggest the configuration of our asynchronous MMI resulted in an equitable process particularly with the familiarisation enabled through the practise portal [15]. We note the higher Cronbach's alpha for disability compared with non-disability. While this is reassuring, it merits further investigation with a larger sample size.

Rice [29] (p452) suggests that social presence or the "degree to which a medium is perceived as conveying the presence of the communicating participants" impacts on applicant acceptability. Social presence plays a central role in trust, enjoyment, and the perceived usefulness of the technological medium [28]. There was by definition an absence of actual social presence in the asynchronous

For peer review only - http://bmjopen.bmj.com/site/about/guidelines.xhtml

modality. However, we sought to mitigate this through softening the intersection between human and technology though design. The majority said they found the system intuitive/very intuitive and simple to use. This infers that the user interface design and inclusive language may have contributed towards a positive experience.

Applicants in this study (>66%) were either accepting or very accepting of the online asynchronous MMI with around one third (37%) agreeing we should 'definitely return' to face-to-face interviews. Notably, only 2% (n= 2) of applicants said they were 'not at all accepting' of the asynchronous MMI. The majority of applicants were 20 years or under. It could be suggested that a younger demographic are more familiar with and accepting of online technology and see it as part of their day-to-day lives. However, these data signal that there was no difference in reliability for those under 20 years compared to over 20 years old and that applicant performance was not impacted by their age.

Applicants' experiences, particularly perceptions of fairness, are of paramount concern for universities. The implications of fairness can extend to post-interview outcomes including offer/acceptance rates. It has been suggested that applicants who perceive that recruitment and selection processes are fair are more attracted to organisations [30,31,32]. Concurring with Brenner et al [12] applicants reported 'perceived fairness' as one of their top three positives of the asynchronous interview system. Their reasons include reduced travel costs and time away from caring responsibilities, as well as enhanced familiarisation of the process through the practice portal. MMI interviews were pre-recorded using inclusive language by diverse staff, representative of the University community. Additional time and an intuitive system user interface appeared to help meet the needs of neurodiverse applicants.

Incorporating the ten fairness principles were not difficult as many were low-cost design features that appeared to be impactful and generically applicable. We suggest these should become a default approach for online interviews used in health professional selection to enable applicant performance

For peer review only

optimisation. In view of the paucity of published evidence [15] these novel insights are informative as we inevitably move toward a technology-augmented future where asynchronous video interviews are considered a modality that is here to stay [15].

We received a 60:40 ratio of positive to negative comments from interview assessors. The largest contributor (62%) to the overall feedback related to positivity around convenience including ability to prioritise workload, flexibility, speed, and reduced stress. This was followed by ease of navigation and reduced bias. These findings corroborate evidence garnered outside the field of healthcare where asynchronous interviews have been found to be faster, cheaper, require less employee time and open the applicant funnel to allow more people to be interviewed than would otherwise have had the opportunity [15]. Nevertheless, communication skills are central to the role of a health professional and are assessed as a generic skill/attribute in each MMI question at this university. One sixth of interview assessors said they did not feel communication skills could be assessed while over a third stated they could in the asynchronous modality. Further research is warranted to better understand the intersection between humans and technology including barriers and enablers to effective communication and communication assessment.

The largest contributing negative comment (30%) focused on a perception that the process was less personal. We might have anticipated this to be higher. Steps being considered to enhance personal connection include live chats and Q&A sessions as well as increasing the number of 'offer holder-days' provided by the university where applicants are invited onto campus to engage with staff without the stress of an interview clouding the experience. Further research evaluating the effectiveness of this strategy is suggested.

Strengths and limitations of this study

For peer review only - http://bmjopen.bmj.com/site/about/guidelines.xhtml

Invitations to evaluate the system were sent out after applicants received notification of their interview outcome. We understand this may have impacted on their perception of the process however, we were required to adhere to the university's policy.

The study was a theoretically driven mixed-methods cross-discipline approach. However, we acknowledge the generalisability limitations of a single site design, but this was an essential step ahead of a planned large multi-site international evaluation.

While the sample size is large, the low response rate from applicants is a potential limitation and may infer selection bias. Users' views could be impacted by many factors outside the scope of this research for example past experiences for which we were unable to account for.

It was not possible to conduct a comparison study 'pre/post system optimisation'. Covid necessitated a move to online interviews in unprecedented times. Data were not collected on applicant or interviewer views at the time given the burdens they were already facing. During that time however, we explored how fairness could be optimised through a review of published literature with findings embedded in our system (Appendix 1). In a high stakes admission process, it would be ethically wrong to conduct a prospective study now to compare with and without the 10 principles for fairness given the apparent benefits.

Collapsing applicant data into UK/Ireland and Non-UK Ireland was a necessary pragmatic decision based on lack of consistent reporting of ethnicity between the university (who did not routinely retain applicant ethnicity data until enrolment) and the UK University Central Admissions System (UCAS).

Rigour

In spite of reassurances in all communications, we were mindful that applicants might be concerned that their evaluation could impact on their interview outcome hence the invitations were sent once offer/reject decisions had been communicated to applicants.

All data in this evaluation were independently analysed and peer reviewed by multiple authors (AC, JH, SR, PT). The qualitative content analysis, was undertaken by two authors independeltly (AC, SR). A

For peer review only

<5% difference was noted between authors' findings. A compromise was mutually agreed in instances where this occurred.

Conclusion

With ten principles for fairness designed in, these findings suggest the asynchronous online interview is reliable, equitable, time-efficient, and acceptable. It is a moral imperative that healthcare workforces represent the societies which they serve, however unintended bias can influence selection decisions. In the absence of generically available consensus guidance on how fairness can be optimised in online interviews, these novel insights are applicable internationally across selection to health professions. Embedding fairness into the design of online interviews is relatively straightforward and low cost to implement. These data advance our understanding which is vital as we inevitably more towards a technology augmented future in the context of global workforce pressures.

Figure 1: Asynchronous MMI infographic.

For peer review only

For peer review only - http://bmjopen.bmj.com/site/about/guidelines.xhtml

Contributor statement

Author AC is guarantor. Authors AC, LG and Ach contributed to the technical development. Authors AC, LG, Ach, JH, PT, AK, SR contributed to the study design, data collection, drafting and revisions. Authors AC, JH, SR, PT contributed to the data analysis. Author Ach is no longer directly affiliated to the university but was at the commencement of this study.

Competing interests statement

Authors AC and LG are co-founders and Ach is an employee of Sammi-Select Lt, funded by UKRI as a spinout company from the University of Surrey to build the platform.

Funding

This work was supported with funding from Innovate UK grant number 37043.

Data sharing statement

Data sets used and/or analysed are available on reasonable request. Detailed technical information is withheld due to commercial sensitivity.

Ethics statement

This study received a favourable ethical opinion (FEO) from the University Research Ethics committee (UEC/2017/111/FHMS). Participants gave informed consent where personal data was included.

Acknowledgements

Grateful thanks to staff, students, practice partners and service users without whom this work would not be possible.

For peer review only

References

1. Council of Deans of Health Equality, Diversity, and Inclusion: https://www.councilofdeans.org.uk/category/policy/equality-diversity-and-inclusion/. Accessed 06.03.23.
2. United Nations Department of Economic and Social Affairs 17 Sustainable Development Goals: https://sdgs.un.org/goals. Accessed 06.03.23.
3. https://www.oecd.org/publications/the-post-covid-19-rise-in-labour-shortages-e60c2d1c-en.htm
4. Sabesan V, Kapur N, Zemanek K, Levitt D, Vu T, Erp A. Implementation and evaluation of virtual multiple mini-interviews as a selection tool for entry into paediatric postgraduate training: A Queensland experience. Medical Teacher Published online: 30 Aug 2021. https://doi.org/10.1080/0142159X.2021.1967906
5. Cleland J, Chu J, Lim S, Low J, Lowe-Beer N, Kwek T. COVID-19: designing and conducting an on-line mini-multiple interview (MMI) in a dynamic landscape. Medical Teacher. 2020. May: 1-5. https://doi.org/10.1080/0142159X.2020.1762851.
6. Kok K, Chen L, Idris F, Mumin N, Ghani H, Zulkipli. Conducting multiple mini-interviews in the midst of COVID-19 pandemic. Korean J Med Educ. 2020 Dec; 32(4): 281–289. Published online 2020 Oct 28. doi: 10.3946/kjme.2020.175.
7. Fuller, R., Goddard, V., Nadarajah, V., Treasure-Jones, T. Yeates, P., Scott, K., Webb, A., Valter, K., Pyorala, E. 2022. Technology enhanced assessment: Ottawa consensus statement and Recommendations. Medical Teacher Pages 836-850 https://doi.org/10.1080/0142159X.2022.2083489
8. Patterson, F, Roberts, C, Hanson, M, Hampe, W, Eva, K, Ponnamperuma, G, Magzoub, M, Tekianhand, A, Cleland, J. Ottawa consensus statement: Selection and recruitment to the

healthcare professions. Medical Teacher. 2018;40(11):1-11. 10.1080/0142159X.2018.1498589

9. Eva K, Rosenfeld J, Reiter H, Norman G. An admissions OSCE: the multiple mini-interview. *Medical Education*. 2004; 38: 314-326.

10. Yusoff J. Multiple Mini Interview as an admission tool in higher education: Insights from a systematic review. J Taibah Univ Med Sci. 2019 Jun; 14(3): 203–240. doi: 10.1016/j.jtumed.2019.03.006

11. Selvam R, Hu R, Musselman R, Raiche I, Mclsaac, D, Moloo H (2021) Video-based interviewing in Medicine: A Scoping Review. Doi: https://doi.org/10.21203/rs.3.rs-468538/v1

12. Brenner, F. S., Ortner, T. M., & Fay, D. (2016). Asynchronous video interviewing as a new technology in personnel selection: The applicant's point of view. *Frontiers in Psychology, 7*, 863.

13. Huppert L A, Hsiao E C, Cho K C *et al.* Virtual interviews at graduate medical education training programs determining evidence-based best practices. *Acad Med* 2020. doi: 10.1097/ACM.0000000000003868.

14. Vining CC, Eng OS, Hogg ME, et al. Virtual Surgical Fellowship Recruitment During COVID-19 and Its Implications for Resident/Fellow Recruitment in the Future. Ann Surg Oncol. 2020;27(Suppl 3):911- 915. doi:10.1245/s10434-020-08623-2

15. Lukacik, E., Bourdage, J., Roulin, N. 2022. Into the void: A conceptual model and research agenda for the design and use of asynchronous video interviews. Human Resources Management Review 32 , 100789

16. Bambra, C., Joyce, K., Bellis, M., Greatley, A., Greengross, S., Hughes, S., Lincoln, P. Lobstein, T., Naylor, C., Salay, R. Wiseman, M., Maryon-Davis, A. 2010 Reducing health inequalities in priority public health conditions: using rapid review to develop proposals for evidence-based

For peer review only - http://bmjopen.bmj.com/site/about/guidelines.xhtml

policy. *Journal of Public Health*, Volume 32, Issue 4, December 2010, Pages 496–505, https://doi.org/10.1093/pubmed/fdq028

17. http://mixedmethodsappraisaltoolpublic.pbworks.com/w/file/fetch/127916259/MMAT_2018_criteria-manual_2018-08-01_ENG.pdf

18. Olsen E, Eoyang G. Facilitating organisational change: lessons from complexity science. San Francisco: Jossey-Bass; 2001.

19. Gilliland, S. 1983 The perceived fairness of selection systems: an organisational justice perspctive. Academy of Management Review Vol 18, No 4, pp 694-734

20. Qualtrics accesible at: https://www.qualtrics.com/uk/

21. Stata accessible at: https://www.stata.com/

22. Horn, J. L. (1965). A Rationale and Test for the Number of Factors in Factor Analysis. Psychometrika, 30, 179-185. https://doi.org/10.1007/BF02289447

23. Timmerman, M. E., & Lorenzo-Seva, U. (2011). Dimensionality assessment of ordered polytomous items with parallel analysis. *Psychological Methods, 16*(2), 209–220. https://doi.org/10.1037/a0023353

24. Lorenzo-Seva, U., Ferrando, P.J., 2006. FACTOR: a computer program to fit the exploratory factor analysis model. Behavior Research Methods 38, 88–91.

25. MPlus version 8. Los Angeles, CA: Muthén & Muthén, 2022

26. Hsieh H, Shannon S. Three Approaches to Qualitative Content Analysis. Qualitative Health Research. November.2005. http:// doi.org/10.1177/1049732305276687

For peer review only - http://bmjopen.bmj.com/site/about/guidelines.xhtml

27. Schmid, J., & Leiman, J. M. (1957). The development of hierarchical factor solutions. *Psychometrika*, *22*(1), 53–61.

28. Schinkel, S., van Vianen, A., & Van Dierendonck, D. (2013). Selection fairness and outcomes: A field study of interactive effects on applicant reactions. *International Journal of Selection and Assessment, 21*(1), 22–31.

29. Rice, R. E. (1993). Media appropriateness: Using social presence theory to compare traditional and new organizational media. *Human Communication Research, 19*(4), 451–484.

30. Oh, C. S., Bailenson, J. N., & Welch, G. F. (2018). A systematic review of social presence: Definition, antecedents, and implications. *Frontiers in Robotics and AI, 5*, 114.

31. Chapman, D. S., Uggerslev, K. L., Carroll, S. A., Piasentin, K. A., & Jones, D. A. (2005). Applicant attraction to organizations and job choice: A meta-analytic review of the correlates of recruiting outcomes. *Journal of Applied Psychology, 90*(5), 928–944. Chapman, D. S., Uggerslev, K. L., & Webster, J. (2003). Applicant reactions to face-to-face and technology

32. Truxillo, D. M., Bauer, T. N., Campion, M. A., & Paronto, M. E. (2002). Selection fairness information and applicant reactions: A longitudinal field study. *Journal of Applied Psychology, 87*(6), 1020–1031.

For peer review only - http://bmjopen.bmj.com/site/about/guidelines.xhtml

For peer review only

For peer review only - http://bmjopen.bmj.com/site/about/guidelines.xhtml

Figure 1: Asynchronous MMI infographic.

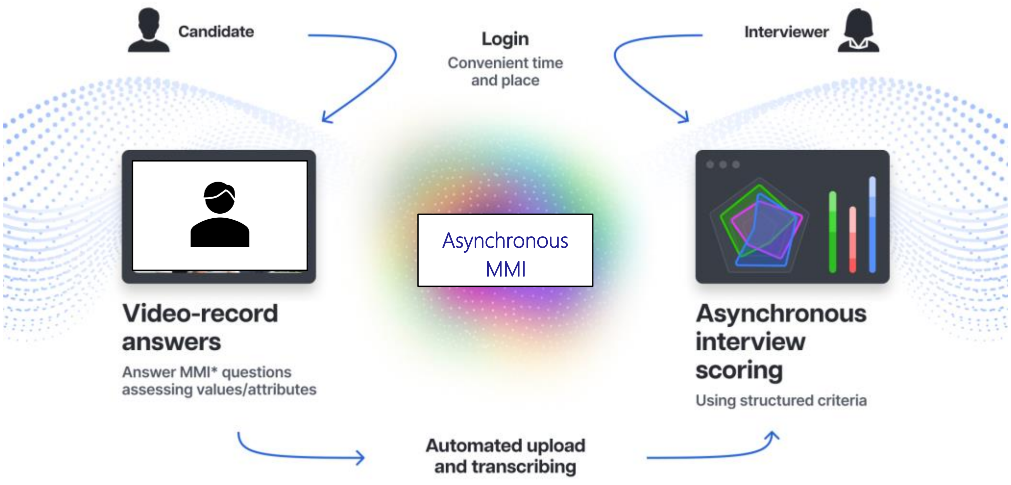

For peer review only

Appendix 1 Rapid Review Key References in Health Professions Selection

Rapid review of published literature

To inform the design, we undertook a rapid review of literature published between 2011-2021, guided by a five-stage process[18] to help us understand how video-based interviews compare with face-to-face in terms of implementation and fairness including what makes for an optimised experience.

The databases searched included: Medline, PsycInfo, Embase, ProQuest (BNI) and Google Scholar/Google (grey literature). We included original articles published in English that reported studies involving candidates interviewed using video with/without face-to-face methods in health professions selection. Articles focusing on psychometric tests and situational judgement tests were excluded. For quality appraisal we used the Mixed Methods Appraisal Tool (MMAT)[19] combined with independent reviewer screening.

After review and removal of irrelevant and duplicates, 49 articles were included, 27 published pre-Covid (before Jan 2020) and 22 between Jan 2020 – Oct 2021 (Appendix 1). Of these, >90% originated in the US, the rest from Australia, Korea, and the UK. The majority were conducted in admissions to Medicine with two from Pharmacy and one Veterinary Medicine. Study methodologies included single site cross-sectional/cohort or quasi-experimental studies. Meta-analysis/meta-synthesis were not possible due to study heterogeneity. Each article was read by up to two of the research team looking for insights and recommendations to optimise accessibility and fairness in online interviews.

Key References

35. Ballejos, M. P., Oglesbee, S., Hettema, J., Sapien, R. An equivalence study of interview platform: Does videoconference technology impact medical school acceptance rates of different groups?. Advances in Health Sciences Education: Theory and Practice. 2018;23(3):601–610. https://doi.org/http://dx.doi.org/10.1007/s10459-018-9817-2 Page 16/21

36. Bird, SB, Hern, G, Blomkalns, A, Deiorio NM, Haywood Y, Hiller KM, et al. Innovation in Residency Selection: The AAMC Standardized Video Interview. Academic Medicine: Journal of the Association of American Medical Colleges. 2019;94(10):1489–1497. https://doi.org/https://dx.doi.org/10.1097/ACM.0000000000002705

For peer review only - http://bmjopen.bmj.com/site/about/guidelines.xhtml

37. Bowers, K., Comp, G., Kalnow, A., Casey, J., Fraser, W., Lloyd, C., Little, A. Are Standardized Video Interview Scores Predictive of Interview Performance? Western Journal of Emergency Medicine. 2018;S18

38. Breitkopf, DM, Green, IC, Hopkins, MR, Torbenson, VE, Camp, CL, Turner, NS. Use of Asynchronous Video Interviews for Selecting Obstetrics and Gynaecology Residents. Obstetrics and Gynecology. 2019;134(4):9S-15S. https://doi.org/https://dx.doi.org/10.1097/AOG.0000000000003432

39. Chandler, N. M., Litz, C. N., Chang, H. L., Danielson, P. D., N.M., C., C.N., L., & H.L., C. Efficacy of Videoconference Interviews in the Paediatric Surgery Match. Journal of Surgical Education. 2019;76(2):420–426. https://doi.org/http://dx.doi.org/10.1016/j.jsurg.2018.08.010

40. Chung AS, Shah KH, Bond M, Ardolic B, Husain A, Li I, Cygan L, Caputo W, Shoenberger J, van Dermark J, Bronner J, Weizberg M. How Well Does the Standardized Video Interview Score Correlate with Traditional Interview Performance?. The Western Journal of Emergency Medicine. 2019;20(5):726–730. https://doi.org/https://dx.doi.org/10.5811/westjem.2019.7.42731

41. Cleland J, Chu J, Lim S, Low J, Lowe-Beer N, Kwek T. COVID-19: designing and conducting an on-line mini-multiple interview (MMI) in a dynamic landscape. Medical Teacher. 2020. May: 1-5. https://doi.org/10.1080/0142159X.2020.1762851

42. Daram, S. R., Wu, R., Tang, S.-J. J., S.R., D., R., W., Daram, S. R., … Tang, S.-J. J. Interview from anywhere: Feasibility and utility of web-based videoconference interviews in the gastroenterology fellowship selection process. American Journal of Gastroenterology. 2014;109(2):155–159. https://doi.org/10.1038/ajg.2013.278

43. Davis M, Haas M, Gottlieb M *et al.* Zooming In Versus Flying Out: Virtual Residency Interviews in the Era of COVID-19. *AEM Educ Train* 2020; 4: 443-446.

44. Egan, D. J., Husain, A., Bond, M. C., Caputo, W., Cygan, L., Van Dermark, J., et al. Standardized video interviews do not correlate to United States medical licensing examination step 1 and step 2 scores. Page 18/21 The Western Journal of Emergency Medicine. 2019;20(1):87–91. https://doi.org/http://dx.doi.org/10.5811/westjem.2018.11.39730

45. Edje, L., Miller, C., Kiefer, J., & Oram, D. Using Skype as an Alternative for Residency Selection Interviews. Journal of Graduate Medical Education. 2013;5(3):503–505. https://doi.org/10.4300/jgme-d-12-00152.1

46. Gallahue, F. E., Hiller, K. M., Bird, S. B., Calderone Haas, M. R., Deiorio, N. M., Hern, H. G., et al. The AAMC Standardized Video Interview: Reactions and Use by Residency Programs During the 2018 Application Cycle. Academic Medicine: Journal of the Association of American Medical Colleges. 2019;94(10):1506–1512. https://doi.org/https://dx.doi.org/10.1097/ACM.0000000000002714

47. Grova MM, Donohue SJ, Meyers MO, Kim HJ, Ollila DW. Direct Comparison of In-Person Versus Virtual Interviews for Complex General Surgical Oncology Fellowship in the COVID-19 Era. Ann Surg Oncol. 2021;28(4):1908-1915. doi:10.1245/s10434-020-09398-2

48. Hakes, E., Schnapp, B., Ritter, D., Kraut, A., Fallon, S., Brown, K., Westergaard, M. Communication and Professionalism: Comparing Standardized Video Interview Scores to Faculty Gestalt. Society for Academic Emergency Medicine. 2018;S276.

49. Hall, MM, Lewis, JJ, Joseph, JW, Ketterer, AR, Rosen, CL, Dubosh, NM. Standardized Video Interview Scores Correlate Poorly with Faculty and Patient Ratings. The Western Journal of Emergency Medicine. 2019;21(1):145–148. https://doi.org/https://dx.doi.org/10.5811/westjem.2019.11.44054

50. Healy, W. L., Bedair, H., & W.L., H. Videoconference Interviews for an Adult Reconstruction Fellowship: Lessons Learned. The Journal of Bone and Joint Surgery. American Volume. 2017;99(21):e114. https://doi.org/https://dx.doi.org/10.2106/JBJS.17.00322

51. Hopson, L. R., Dorfsman, M. L., Branzetti, J., Gisondi, M. A., Hart, D., Jordan, J., et al. Comparison of the Standardized Video Interview and Interview Assessments of Professionalism and Interpersonal Communication Skills in Emergency Medicine. AEM Education and Training. 2019;3(3):259–268. https://doi.org/https://dx.doi.org/10.1002/aet2.10346

52. Humbert, A., Pettit, K., Mugele, J., Turner, J., Morgan, Z., & Palmer, M. Correlation of the Standard Video Interview Score With an Established Application Review Process. Society for Academic Emergency Medicine. 2018;S98.

53. Huppert L A, Hsiao E C, Cho K C *et al.* Virtual interviews at graduate medical education training programs determining evidence-based best practices. *Acad Med* 2020. doi: 10.1097/ACM.0000000000003868.

---

54. Husain, A., Li, I., Ardolic, B., Bond, M. C., Shoenberger, J., Shah, K. H., et al. The Standardized Video Interview: How Does It Affect the Likelihood to Invite for a Residency Interview?. AEM Education and Training. 2019;3(3):226–232. https://doi.org/https://dx.doi.org/10.1002/aet2.10331

Inzana, K, Vanderstichel, R, Newman, S. Virtual Multiple Mini-Interviews for Veterinary Admissions. https://doi.org/10.3138/jvme-2020-0107 J Of Veterinary Medical Education

56. Joshi, A., Bloom, D. A., Spencer, A., Gaetke-Udager, K., & Cohan, R. H. Video Interviewing: A Review and Recommendations for Implementation in the Era of COVID-19 and Beyond. Academic radiology. 2020;27(9):1316–1322. https://doi.org/10.1016/j.acra.2020.05.020

57. Kok K, Chen L, Idris F, Mumin N, Ghani H, Zulkipli. Conducting multiple mini-interviews in the midst of COVID-19 pandemic. Korean J Med Educ. 2020 Dec; 32(4): 281–289. Published online 2020 Oct 28. doi: 10.3946/kjme.2020.175.

58. Krauss, W., Egan, D., Bond, M., Husain, A., White, M., Taylor, T., et al. Correlation Between Emergency Medicine Residency Applicant's Standardized Video Interview Scores and United States Medical Licensing Examination Results. Society for Academic Emergency Medicine. 2018;S83

For peer review only

Kyong-Jee Kim, Nam Young Lee & Bum Sun Kwon. Benefits and Feasibility of Using Videos to Assess Medical School Applicants' Empathetic Abilities in Multiple Mini Interviews. *Medical Science Educator* volume 31, pages175–181 (2021)C

60. Lewis, J., Hall, M., Joseph, J., Dubosh, N., Ullman, E. Standardized Video Interview Scores Do Not Correlate With Attending Evaluations. Society for Academic Emergency Medicine, 2018;S229.

61. M., S.R., E., L.M., B., M.M., A., F.M., D., J.C., E., … H., F. Initial Experience with a Virtual Platform for Advanced Gastrointestinal Minimally Invasive Surgery Fellowship Interviews. Journal of the American College of Surgeons. 2020;231(6):670–678. https://doi.org/http://dx.doi.org/10.1016/j.jamcollsurg.2020.08.768

62. McAteer, R., Sundaram, S., Harkisoon, S., & Miller, J. Videoconference Interviews: A Timely Primary Care Residency Selection Approach. Journal of Graduate Medical Education. 2020;12(6):737–744. https://doi.org/https://dx.doi.org/10.4300/JGME-D-20-00248.1

63. McHugh, M., Kulstad, C., Van Dermark, J., Bischof, J. Do Standardized or Traditional Interview Questions Correlate With the Standardized Video Interview? Society for Academic Emergency Medicine. 2019;S216

64. Molina, G., Mehtsun, W. T., Qadan, M., Hause, K. C., Raut, C. P., & Fairweather, M. Virtual Interviews for the Complex General Surgical Oncology Fellowship: The Dana-Farber/Partners Experience. Annals of Surgical Oncology. 2020;27(9):3103–3106. https://doi.org/https://dx.doi.org/10.1245/s10434-020- 08778-y

65. Nutter, A., La Rosa, M., Olson, G. Perception of Candidates and Faculty on Maternal Fetal Medicine Fellowship Videoconference Interviewing. Obstetrics and Gynecology. 2020;S75.

Nwora C, M.D.[a] Allred, D, M.D.[b] Verduzco-Gutierrez, M, M.D.[c] Mitigating Bias in Virtual Interviews for Applicants Who are Underrepresented in Medicine. Journal of the National Medical Association Volume 113, Issue 1, February 2021, Pages 74-76

67. Patel T, Bedi H, Deitte L, Lewis P *et al.* Brave New World: Challenges and Opportunities in the COVID-19 virtual interview season. *Acad Radiol* 2020; 27: 1456-1460.

68. Proost, K., Germeys, F., & Vanderstukken, A. Applicants' pre-test reactions towards video interviews: the role of expected chances to demonstrate potential and to use nonverbal cues. European Journal of Work and Organizational Psychology. 2021;30(2):265-273. DOI: 10.1080/1359432X.2020.1817975

69. Sabesan V, Kapur N, Zemanek K, Levitt D, Vu T, Erp A. Implementation and evaluation of virtual multiple mini-interviews as a selection tool for entry into paediatric postgraduate training: A Queensland experience. Medical Teacher Published online: 30 Aug 2021.

https://doi.org/10.1080/0142159X.2021.1967906

70. Schnapp, B. H., Ritter, D., Kraut, A. S., Fallon, S., Westergaard, M. C. Assessing residency applicants' communication and professionalism: Standardized video interview scores compared to faculty gestalt. Western Journal of Emergency Medicine. 2019;20(1):132–137. https://doi.org/http://dx.di.org/10.5811/westjem.2018.10.39709

For peer review only

71. Shah SK, Arora, S, Skipper B, Kalishman S, Timm TC, Smith AY. Randomized evaluation of a web based interview process for urology resident selection. Journal of Urology. 2012;187(4):1380–1384. https://doi.org/http://dx.doi.org/10.1016/j.juro.2011.11.108

72. Singh, N D, DeMesa, DO, MPH, Pritzlaff, S, MD, Jung, M MD, MBA, Green, C MA, LMFT. Implementation of Virtual Multiple Mini-Interviews for Fellowship Recruitment . *Pain Medicine*, Volume 22, Issue 8, August 2021, Pages 1717–1721, https://doi.org/10.1093/pm/pnab141

73. Sripad, A. Videoconference Interviews for Female Pelvic Medicine and Reconstructive Surgery Fellowship During a Pandemic: The Candidate Experience. Female Pelvic Medicine & Reconstructive Surgery. 2020;S181.

74. Staicu, M., Hamby, C., Wychowski, M., Reiss, B. Facetime faceoff: evaluation of video conferencing as a novel pre-interview screen for a PGY-1 pharmacy residency. 2015;E182.

75. Temple, M., Lagzdins, M. Streamlining the residency interview process using Web-based teleconferencing. American Journal of Health-System Pharmacy. 2014;71:697-701. Doi: 10.2145/ajhp130406

76. Tiller D, O'Mara D, Rothnie I, Dunn S, Lee L, Roberts C. Internet-based multiple mini-interviews for candidate selection for graduate entry programmes. Med Educ. 2013;47(8):801-810. doi:10.1111/medu.12224

77. Turpin C, Steele K, Matuk-Villazon O, Rowland, K, Dayton, Horn, K. Rapid Transition to a Virtual Multiple Mini-Interview Admissions Process: A New Medical School's Experience During the COVID-19 Pandemic. Academic Medicine: August 2021 - Volume 96 - Issue 8 - p 1152-1155. doi: 10.1097/ACM.0000000000004179.

78. Ungtrakul T, Lamlertthon W, Boonchoo B, ,Auewarakul C. Virtual Multiple Mini-Interview during the COVID-19 Pandemic. Medical Education Adaptation. **https://doi.org/10.1111/medu.14207**

79. Vadi, M. G., Malkin, M. R., Lenart, J., Stier, G. R., Gatling, J. W., & Applegate, R. L. Comparison of web-based and face-to-face interviews for application to an anaesthesiology training program: a pilot study. International Journal of Medical Education. 2016;7:102–108. https://doi.org/10.5116/ijme.56e5.491a

80. Vining CC, Eng OS, Hogg ME, et al. Virtual Surgical Fellowship Recruitment During COVID-19 and Its Implications for Resident/Fellow Recruitment in the Future. Ann Surg Oncol. 2020;27(Suppl 3):911- 915. doi:10.1245/s10434-020-08623-2

81. Willis, J., Surles, T., Silverberg, M., Kendall, S., LoCascio, H., Gernsheimer, J., Schechter, J., Regan, A., Smith, T. Are Standardized Video Interview Scores Predictive of Interview Performance? Western Journal of Emergency Medicine. 2018;S5-S6.

82. Winfield-Dial, A., Chhabra, N., Schindlbeck, M., & Bowman, S. Demographic Differences Between High and Low Scorers on the Standardized Video Interview. Western Journal of Emergency Medicine. 2018;S48. https://escholarship.org/uc/item/68c0x4pf

For peer review only

83. Winfield-Dial, A., Chhabra, N., Schindlbeck, M., & Bowman, S. Applicant Attitudes Towards the Standardized Video Interview - An Interim Analysis. Western Journal of Emergency Medicine. 2018;S4. https://escholarship.org/uc/item/5xp7f587 Page 17/21

84. Yolanda,S, Wisnu, W, Wahjudi, J and Findyartini A. Adaptation of internet-based multiple mini-interviews in a limited-resource medical school during the coronavirus disease 2019 pandemic. Korean J Med Educ. 2020 Dec; 32(4): 281–289. doi: 10.3946/kjme.2020.175

For peer review only

Appendix 2. Estimated hierarchical confirmatory factor analytic (CFA) model for the SAMMI responses, with a general factor (g) and seven specific factors (f1 to f7), the latter representing the seven questions in the interview. Standardised factor loading are shown.

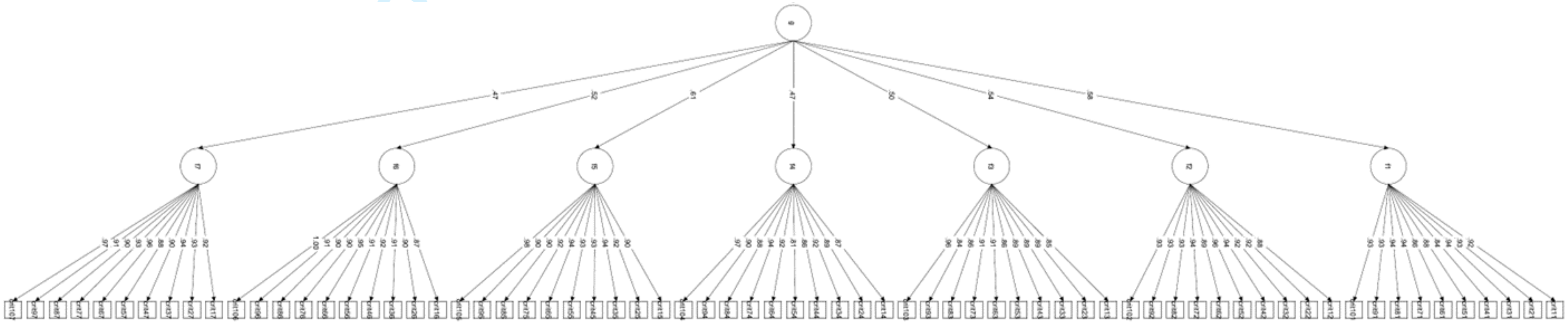

For peer review only - http://bmjopen.bmj.com/site/about/guidelines.xhtml

Appendix 3. Results of a parallel analysis using Pearson correlations on the response data to the SAMMI MMI.

Number of postulated factors	Mean of real data % of variance	Mean of random % of variance	95 percentile of random % of variance
1	29.2*	2.9	3.1
2	11.1*	2.8	3.0
3	9.5*	2.8	2.9
4	8.3*	2.7	2.8
5	7.6*	2.6	2.7
6	7.4*	2.6	2.7
7	6.2*	2.5	2.6
8	1.1	2.5	2.5
9	1.0	2.4	2.5

*Percentage of variance in the responses explained by the number of factors in the real data exceeded that for the random data.

For peer review only

Appendix 4: Cost Evaluation

These data represent an indicative example from this University based on 500 candidates over 7 days doing a seven station, four-minute MMI circuit.

Hours spent	F2F/ Videoconf.	SAMMI®
Pre-interview preparation	20	15
Set up: physical/Zoom rooms	30	0
Co-ordinator time	55	0
Student helpers/staff support	65	0
Post-interview support	10	10
Coordinator time overall	15	15
**Total hours**	**195**	**40**

For peer review only

Supplementary Table 1: Applicant and interviewer top positives and negatives

Applicant				
Positives				
Theme	Sub theme detail	N=158 (%)	% of total comments	Illustrative quote
Ease	Access, intuitive, convenient, flexible, simple	63 (40)	21	*"Quick, not time consuming, simple"*<br>*"Flexible, you can do it at your own time. It reduces stage fright. It is convenient, you can do it in your own home…"*
Reduced stress	More relaxed in own home, take my time, breaks available, start when wish	45 (28)	15	*"Its more relaxing to be in your own home instead of a new environment which for me is much less intimidating resulting in perhaps me performing better in the interview".*<br>*"… there is no external party criticising you in the moment, something which personally takes away a fair amount of nerves and stress from the process…"*
Fairer	Reduced costs (travel), reduced time away from other responsibilities	39 (25)	13	*"Its much easier in regard to travel for those who live far away, don't have the funds or time or who have other commitments".*

	(caring), the practice portal and availability of question text helped neurodiverse applicants			*"Reduces the possibility of bias during the interview process".* *"More inclusive".* *"Another great thing about this was I could do a practice question to get used to the layout of the interview, so I wasn't going into the interview completely blind".*
'Meet' staff	Able to see more of the university's community	9 (7)	3	*"I liked that there were 7 different people asking the questions…"*
Covid safe	Travel not required	2 (1)	1	*"Considering the situation with the pandemic, the online interview has helped to continue the process of admissions in a positive way…"*
Negatives				
Theme	Sub theme detail	N=140 (%)	% of total comments	Illustrative quote
Limited direct communication	Less personal, no conversation	48 (34)	16	*"… Impersonal, I feel like I cant make a connection or read the interviewers body language in the interview which I feel doesn't allow me to act how I usually do, and it felt unnatural".*

For peer review only

MMI	Pressure felt due to the timed methodology, presence of the countdown timer, lack of question face validity.	45 (32)	15	*"4 minutes per question was quite pressuring to fill"* *"Ticking down time was off-putting".* *"Could have asked more personal questions such as why this university and tell us about yourself, there was too many scenario questions which quite put us on the spot".*
Anticipated technical issues	Wi-Fi cut out	27 (19)	9	*"Technical issue (my screen froze) potential upload failure, potential loss of internet connection".*
Don't get vibe of staff	Not directly meeting staff	2 (1)	1	*"Lack of interaction between the student-tutor, the dialogue"*
Cannot ask questions		18 (13)	6	*"Not personalised, not able to ask questions"*
Interviewer				
Positives				
Theme	Sub theme detail	N=132 (%)	% of total comments	Illustrative quote
Convenient	Can prioritise workload. Flexible Quicker	82 (62%)	37	*"Efficient, less time consuming and able to prioritise your workload accordingly and complete interviews in chunks as opposed to one long stint"*

For peer review only

	Less stressful			*Don't have the issue of trying to find someone to cover like when another meeting comes up at the same time…"*
Fairer	Less unconscious bias<br>Reduced travel [costs]	18 (14%)	7	*"Reduced bias from different people asking the questions".*<br>*"Equity... no student gets help with prompts more than others"*
East to navigate	Simple	12 (9%)	5	*"Easy to use with clear instructions"*
Benefits applicants	Less stressed in own environment	12 (9%)	6	*"Separating the two events [interview/applicant day] will help manage anxiety and stress"*
Applicant assessment	Can get a [better] sense of the applicant	4 (3%)	2	*"I feel I can get a sense of the applicant through this process".*
Less worried about technical issues	Not one time/date dependent	3 (2%)	2	*"Much less stressful as I don't have to worry about the internet connection".*
Ability to rewatch	If cause for concern'	1 (1%)	1	*"Ability to rewatch for clarification".*
Negatives				
Theme	Sub theme detail	N=90 (%)	% of total comments	Illustrative quote
Less personal	Don't get feel for applicant	27 (30%)	12	*"I think it's better for applicants to speak to someone in person"*

For peer review only

				"I question whether it produces the same quality and depth of response".
Critique of MMI methodology	Timed circuit/countdown timer	22 (24%)	10	*"Encourages to me to make a snap decision"* *"Cant ask follow-on questions…permits no probing".*
Communication assessment and build rapport difficult	Less able to assess non-verbal communication, social skills, spontaneous cognitive ability.	21 (23%)	9	*"Little in the way of holism".* *"Disconnect perhaps for applicants".* *"Does not allow markers to assess a candidates ability to respond and adapt to others".*
Limited support for nervous/stressed applicant	Can not help if upset or stressed	8 (9%)	4	*"No opportunity to provide any support if the candidate appears upset.. if they are struggling mentally or emotionally"..*
Tech related process concerns	Tech issues Stress of Zoom/Teams online mode Repetitive watching videos Disadvantages those not familiar with tech	4 (5%)	2	*"Robotic process"* *"Repetitive and boring watching multiple videos"* *"Some people find Teams/Zoom very stressful and therefore we dont see the best of the candidate".* *"Technical issues for some student may give them a disadvantage".*
Did not have any	Stated 'none'	5 (6%)	2	*"Don't have any"*

For peer review only

				"None to mention".
University marketing	Does not show university as warm and welcoming	3 (3%)	1	*"We are very nice and they do not see that"* *"Current students often state they chose Surrey due to the friendliness of the staff when first meeting them, my concern is if this is the first impression".*

For peer review only

STROBE Statement—checklist of items that should be included in reports of observational studies

	Item No	Recommendation	Page No
**Title and abstract**	1	(*a*) Indicate the study's design with a commonly used term in the title or the abstract	1
		(*b*) Provide in the abstract an informative and balanced summary of what was done and what was found	3-4
**Introduction**			
Background/rationale	2	Explain the scientific background and rationale for the investigation being reported	5-9
Objectives	3	State specific objectives, including any prespecified hypotheses	9
**Methods**			
Study design	4	Present key elements of study design early in the paper	9-10
Setting	5	Describe the setting, locations, and relevant dates, including periods of recruitment, exposure, follow-up, and data collection	10
Participants	6	(*a*) *Cohort study*—Give the eligibility criteria, and the sources and methods of selection of participants. Describe methods of follow-up<br>*Case-control study*—Give the eligibility criteria, and the sources and methods of case ascertainment and control selection. Give the rationale for the choice of cases and controls<br>*Cross-sectional study*—Give the eligibility criteria, and the sources and methods of selection of participants	11
		(*b*) *Cohort study*—For matched studies, give matching criteria and number of exposed and unexposed<br>*Case-control study*—For matched studies, give matching criteria and the number of controls per case	
Variables	7	Clearly define all outcomes, exposures, predictors, potential confounders, and effect modifiers. Give diagnostic criteria, if applicable	12
Data sources/ measurement	8*	For each variable of interest, give sources of data and details of methods of assessment (measurement). Describe comparability of assessment methods if there is more than one group	*12*
Bias	9	Describe any efforts to address potential sources of bias	30
Study size	10	Explain how the study size was arrived at	11
Quantitative variables	11	Explain how quantitative variables were handled in the analyses. If applicable, describe which groupings were chosen and why	12
Statistical methods	12	(*a*) Describe all statistical methods, including those used to control for confounding	12
		(*b*) Describe any methods used to examine subgroups and interactions	12
		(*c*) Explain how missing data were addressed	12
		(*d*) *Cohort study*—If applicable, explain how loss to follow-up was addressed<br>*Case-control study*—If applicable, explain how matching of cases and controls was addressed<br>*Cross-sectional study*—If applicable, describe analytical methods taking account of sampling strategy	12
		(*e*) Describe any sensitivity analyses	

For peer review only - http://bmjopen.bmj.com/site/about/guidelines.xhtml

For peer review only

**Results**			
Participants	13*	(a) Report numbers of individuals at each stage of study—eg numbers potentially eligible, examined for eligibility, confirmed eligible, included in the study, completing follow-up, and analysed	12<br>16-17
		(b) Give reasons for non-participation at each stage	
		(c) Consider use of a flow diagram	
Descriptive data	14*	(a) Give characteristics of study participants (eg demographic, clinical, social) and information on exposures and potential confounders	13
		(b) Indicate number of participants with missing data for each variable of interest	
		(c) *Cohort study*—Summarise follow-up time (eg, average and total amount)	
Outcome data	15*	*Cohort study*—Report numbers of outcome events or summary measures over time	
		Case-control study—Report numbers in each exposure category, or summary measures of exposure	
		Cross-sectional study—Report numbers of outcome events or summary measures	*13-14*
Main results	16	(*a*) Give unadjusted estimates and, if applicable, confounder-adjusted estimates and their precision (eg, 95% confidence interval). Make clear which confounders were adjusted for and why they were included	14-27
		(*b*) Report category boundaries when continuous variables were categorized	
		(*c*) If relevant, consider translating estimates of relative risk into absolute risk for a meaningful time period	
Other analyses	17	Report other analyses done—eg analyses of subgroups and interactions, and sensitivity analyses	15-16
**Discussion**			
Key results	18	Summarise key results with reference to study objectives	27
Limitations	19	Discuss limitations of the study, taking into account sources of potential bias or imprecision. Discuss both direction and magnitude of any potential bias	30
Interpretation	20	Give a cautious overall interpretation of results considering objectives, limitations, multiplicity of analyses, results from similar studies, and other relevant evidence	28
Generalisability	21	Discuss the generalisability (external validity) of the study results	30
**Other information**			
Funding	22	Give the source of funding and the role of the funders for the present study and, if applicable, for the original study on which the present article is based	2

*Give information separately for cases and controls in case-control studies and, if applicable, for exposed and unexposed groups in cohort and cross-sectional studies.

For peer review only

For peer review only - http://bmjopen.bmj.com/site/about/guidelines.xhtml
